# Repetition-related reductions in neural activity support improved behavior through increases in oscillatory power

**DOI:** 10.1038/s42003-026-10668-0

**Published:** 2026-07-21

**Authors:** Adrian W. Gilmore, Leonardo Claudino, Cassandra M. Levesque, Anna M. Agron, Peter J. Molfese, Vinai Roopchansingh, Michael D. Rugg, Stephen J. Gotts, Alex Martin

**Affiliations:** 1 Section on Cognitive Neuropsychology, Laboratory of Brain and Cognition, National Institute of Mental Health, National Institutes of Health, Bethesda, MD USA; 2 Department of Psychological and Brain Sciences, University of Delaware, Newark, DE USA; 3 Center for Multimodal Neuroimaging, National Institute of Mental Health, National Institutes of Health, Bethesda, MD USA; 4 Functional MRI Facility, National Institute of Mental Health, National Institutes of Health, Bethesda, MD USA; 5 Center for Vital Longevity, University of Texas at Dallas, Dallas, TX USA

**Keywords:** Cognitive neuroscience, Learning and memory, Human behaviour

## Abstract

Repeatedly processing an object leads to subsequent behavioral improvements in its identification and reductions in associated neural activity, a form of neural processing efficiency that multiple theoretical models have attempted to explain. Using simultaneous EEG/fMRI in humans during object naming, we find that stimulus-driven oscillatory power from high (gamma) to low frequencies (theta) increases and resolves earlier with repetition in task-engaged frontal and occipitotemporal brain regions identified as showing repetition suppression in fMRI. Repetition-related increases and decreases in gamma oscillatory power in these regions were correlated with behavioral priming across subjects (|*r*s| ≥ 0.39) and additionally with fMRI repetition suppression in left frontal cortex (*r* = -0.37). Similar correlations were observed between behavioral priming and oscillatory power changes when effects were examined within subjects (|*r*s| ≥ 0.34). Collectively, these data provide multimodal support for a novel mixture of the previously proposed Synchrony and Facilitation models.

## Abstract (web summary)

Simultaneous EEG and fMRI recording suggests that repetition-related changes in behavior and neural activity may result from earlier, more rapid increases in synchronized neural activity in task-involved brain regions.

## Introduction

It is a foundational phenomenon in cognitive neuroscience that repeated experiences with a stimulus lead to behavioral improvements in the task being performed (repetition priming) while simultaneously leading to reductions in neural activity (repetition suppression; RS). This dual neural/behavioral phenomenon has been observed in humans^1,2^, non-human primates^3,4^, and rodents^5,6^. Across this range of species, repetition-related changes consistently appear to be stimulus-specific and have both short-term and long-term components^7^. The phenomenon is also notable in that it does not depend strongly on the hippocampus and other medial temporal lobe structures, as it is largely spared in amnesia^8–10^. Rather, it prominently involves the neocortical regions engaged by the task being performed. Left lateral prefrontal cortex appears to play a particularly critical role in mediating the relationship between neural and behavioral effects^11–14^, possibly through the mapping of behavioral responses to specific perceptual inputs^15,16^.

However, the co-occurrence of priming and RS poses a mechanistic puzzle. How might reducing neural activity support improvements in—rather than impairments of—the behavior in question? “Efficiency” is often invoked to describe the phenomenon, but it does not, by itself, imply any specific mechanism^17^. However, several theoretical models have been proposed to explain this coupling. The Facilitation model^18,19^ proposes that neural activity initiates and resolves more quickly for repeated stimuli, with rapid kinetics that are difficult to capture given the slow time course of BOLD fMRI responses when studied in humans. The Sharpening model^1,20^ holds that RS occurs primarily in cells that are poorly tuned to the repeated stimulus, thereby increasing neural selectivity despite an overall activity reduction. The Synchrony model^7,21^ holds that cells fire in a more synchronized manner with repetition, leading to larger amplitude population fluctuations that require fewer spikes for effective signal propagation. Finally, the Predictive Coding model^22^ holds that the brain is composed of generative neural networks that attempt to minimize differences over repetitions between top-down expectations and bottom-up sensory evidence, with observed neural activity reflecting residual prediction error. While all models have some experimental support, none has yielded strong direct empirical evidence that ties their proposed mechanisms to the magnitude of both RS and repetition priming^23^.

In humans, one of the main limitations to progress has been a lack of neuroimaging methods that simultaneously offer high spatial resolution, high temporal resolution, and whole-brain coverage—all of which seem required to better understand the relation between behavioral improvements and reductions in neural activity. In the current study, we address this limitation by utilizing simultaneous EEG/fMRI while participants name pictures of common objects that are either novel (presented a single time) or repeated during the experiment. fMRI provides a spatially precise means of identifying brain regions that exhibit RS. The simultaneously acquired EEG data can then be source localized to the cortex at the anatomical locations identified by fMRI, providing an estimate of rapid electrophysiological neural activity at those locations that cannot be obtained by BOLD fMRI alone.

Based on prior literature, electrophysiological effects might be expected to emerge in the gamma band or in the theta/alpha bands. Changes in induced power in the gamma range have previously been associated with stimulus repetition in both microelectrode recordings in animals^24–27^ and EEG in humans^28–30^. On the other hand, MEG data in humans have found changes in evoked power and phase locking in frontal and fusiform regions in theta, alpha, and low beta frequencies^21,31^. Although the current experiment will consider effects ranging from delta to gamma, there is a priori evidence to suggest that the above frequency bands, in particular, may speak to the relation between repetition suppression and repetition priming.

## Results

### Repetition priming, fMRI repetition suppression, and scalp ERP repetition effects are observed in object naming

**Fig. 1:**
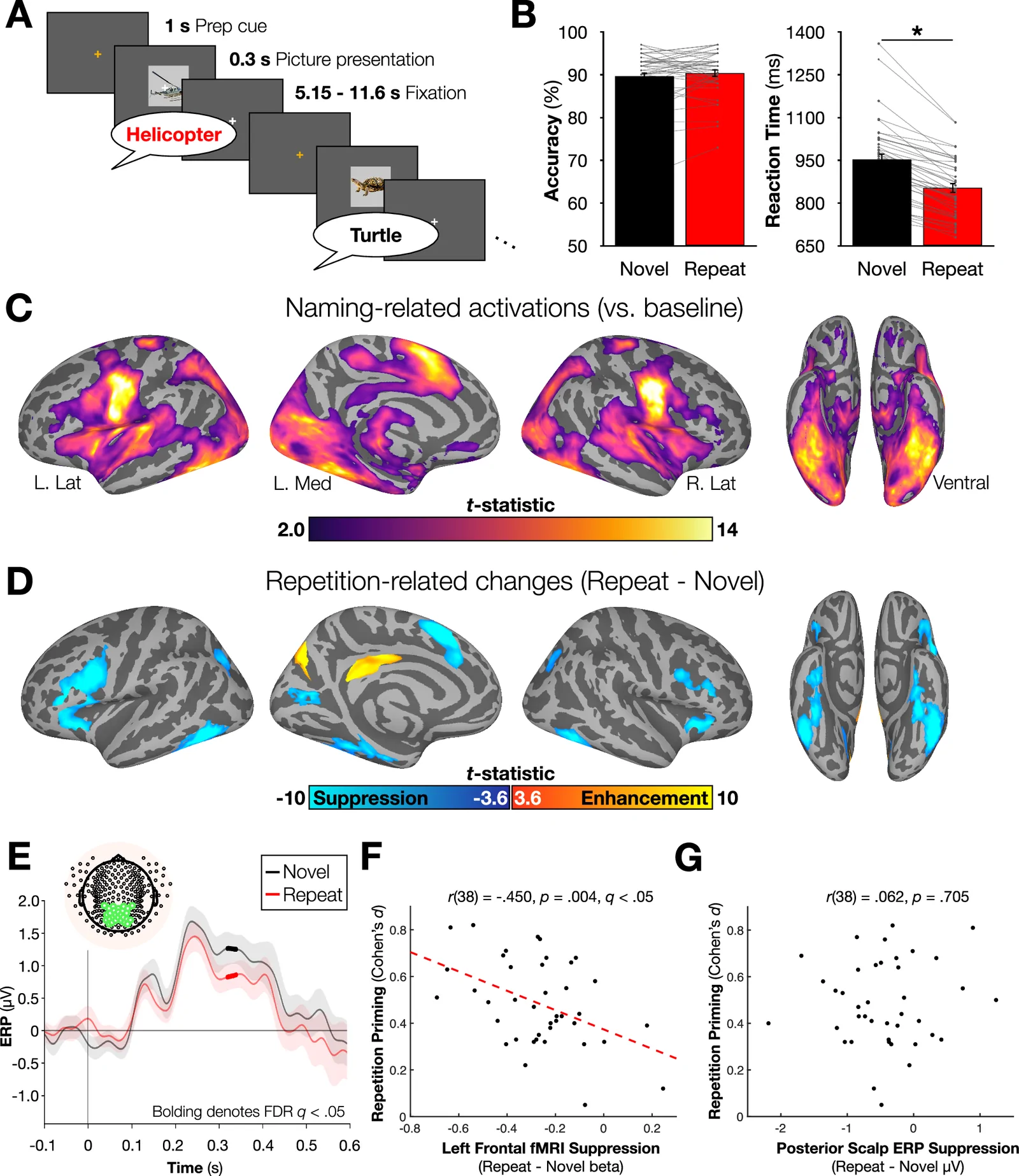
Behavioral, fMRI, and scalp ERP results associated with the object naming task. **A** The task utilized a slow event-related design. A preparatory (prep) cue was provided 1 s before each stimulus appeared. Stimuli appeared for 300 ms and were followed by a variable period of 5.15–11.6 s. Participants were instructed to speak the name of each image as quickly as possible. Novel and Repeat stimuli (items previously named approximately 75 min prior to brain imaging) were pseudorandomly intermixed. **B** Naming accuracy did not differ between the Novel and Repeat conditions, but participants demonstrated repetition priming by naming Repeat stimuli significantly faster than Novel stimuli. **C** fMRI identified significant activations in multiple regions across the cortex. **D** Within Naming-activated regions, significant repetition suppression was noted in multiple regions, including bilateral frontal and fusiform cortex. Repetition-related increases in activity (repetition enhancement) were observed in the precuneus and mid-cingulate/rostral posterior cingulate cortex. **E** Significant repetition suppression of scalp ERP signals was observed over an a priori-defined posterior electrode montage. **F** Repetition priming was significantly correlated with fMRI repetition suppression in left frontal cortex, but **G** not with ERP suppression over the posterior electrode montage. Statistics are based on *N* = 40 independent participants. * denotes *p* < .001. Error bars on bar plots and shaded curves around timeseries data reflect *SEM*.

Forty participants overtly named 100 pictures of common objects (living things; inanimate objects) twice each, approximately one hour prior to brain imaging. During EEG/fMRI, participants then named the same 100 pictures once more (Repeat condition), randomly intermixed with a set of 100 new objects (Novel condition) that were matched in lexical properties and name frequency to the repeated set (Fig. 1A). Accuracy and response times were recorded for each trial. As in prior studies, repeated pictures were named faster than novel pictures [Repeat $\bar{M}$ (SD) = 853.1 (16.6) ms, Novel $\bar{M}$ (SD) = 952.3 (21.3) ms; *t*(39) = 11.46, *p* < 0.001, Cohen’s *d* = 1.81; Fig. 1B]. Accuracy did not differ significantly between conditions (*p* = 0.234). Also consistent with prior studies^23,32^, picture naming led to above-baseline activity in fMRI in occipitotemporal and frontal brain regions (Fig. 1C), with RS prominently observed for repeated pictures in left lateral frontal cortex, anterior cingulate cortex (extending into the pre-supplementary motor area; hereafter “dACC/pre-SMA”), and the left and right fusiform gyri (all *p*s < 0.001, FDR corrected to *q* < 0.05; Fig. 1D). In the simultaneously acquired EEG, scalp event-related potentials (ERPs) exhibited a decreased amplitude over posterior electrodes for Repeat stimuli. Significant effects were observed during the late ERP component from 313 to 345 ms post-stimulus onset (all *p*s < 0.0028, *q* < 0.05; Fig. 1E). Similar ERP effects over posterior electrodes have been reported in prior EEG studies, although typically with much shorter delays between stimulus repetitions (e.g., refs. ^28,29,33,34^).

To establish the relation between priming and RS in the current study, we first converted the priming scores for each participant to Cohen’s *d* effect sizes (as in ref. ^14^) and then correlated priming magnitude with RS in fMRI and in the scalp ERPs. Across participants, priming magnitude was significantly correlated with the magnitude of RS in left frontal cortex in fMRI, as has been observed previously^23^ [*r*(38) = −0.450, *p* = 0.0036, *q* < 0.05; Fig. 1F], with non-significant correlations observed for the dACC/pre-SMA [*r*(38) = −0.294, *p* = 0.065] and the left fusiform gyrus [*r*(38) = −0.282, *p* = 0.078]. In contrast, RS in the scalp ERPs was not correlated with priming magnitude [*r*(38) = 0.062, *p* = 0.705; Fig. 1G], nor was it related to fMRI RS in any region (all |*r*s| < 0.175, *p*s > 0.28). Partialling out scalp ERP RS also had little impact on the relationship between left frontal RS and priming magnitude [partial *r*(37) = −0.447, *p* = 0.0043]. Taken together, these results suggest that left frontal RS in fMRI and RS in scalp ERPs index different aspects of neural activity, and it is the left frontal RS in fMRI that is most strongly associated with repetition priming.

### EEG oscillatory power rises and falls earlier with stimulus repetition and does so in a manner correlated with the magnitude of behavioral priming

**Fig. 2:**
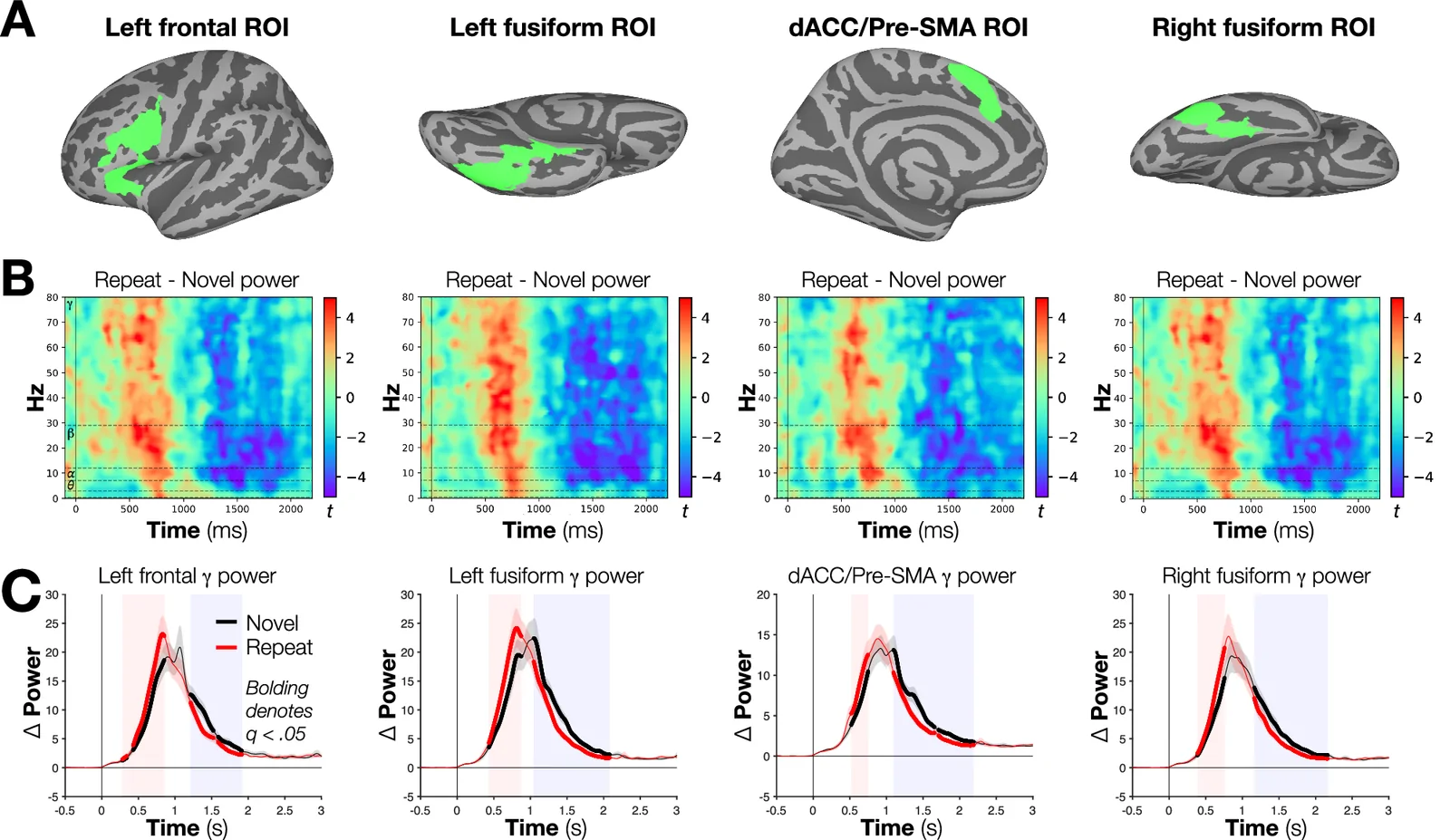
fMRI-identified regions of interest and induced power differences over time. **A** Regions of interest were identified in locations where masks of Naming-related activation (Fig. 1B) and Repetition Suppression (Fig. 1C) intersected at the group level. **B** After source estimation of the EEG data, differences in Novel and Repeat conditions were plotted as a function of time and frequency in each region of interest. In all cases, a significant “early” period of Repeat > Novel activity was observed, with a “late” period exhibiting significant Novel > Repeat effects. **C** “Early” and “late” period differences can be appreciated by examining average activity in the gamma band. Bolded sections indicate periods of significant differences in power (FDR corrected to *q* < 0.05). Statistics are based on *N* = 40 independent participants. Shaded curves around timeseries data reflect *SEM*.

Whereas the analysis of scalp ERP RS enabled comparisons with prior relevant literature, the simultaneous acquisition of fMRI data allowed for a more targeted investigation of electrophysiological effects by examining source-estimated fluctuations of the EEG signal within fMRI-defined regions of interest. Trial-level EEG signals for dipoles perpendicular to the cortical surface were estimated from the scalp data for the four main activated regions showing RS in fMRI (left frontal, dACC/pre-SMA, left and right fusiform gyri; Fig. 2A) and decomposed into wavelets for time-frequency analyses (see “Methods” section). Strong effects of stimulus repetition were observed in induced rather than evoked power (Supplementary Figs. 1 and 2) across the gamma (30–80 Hz), beta (13–29 Hz), alpha (8–12 Hz), and theta (4–7 Hz) frequency bands in all four fMRI RS regions [all *t*s(39) > 2.206, *p*s < 0.0334, *q*s < 0.05; Fig. 2B]. These followed a stereotypical pattern, with an early increase in induced power for the Repeat relative to the Novel condition around 300-500 ms post-stimulus (earliest onsets detected in the gamma frequency range at 280 ms, 431 ms, 519 ms, and 385 ms in the left frontal, left fusiform, dACC/pre-SMA and right fusiform regions, respectively) through approximately 800–900 ms post-stimulus (latest offsets in the alpha frequency range at 888 ms, 870 ms, 892 ms, and 917 ms in the left frontal, left fusiform, dACC/pre-SMA and right fusiform regions, respectively; Supplementary Figs. 1 and 2; Supplementary Table 1; see bolded segments backed by a light red background in Fig. 2C for visualization effects in the gamma band, specifically). This was followed by a more rapid decrease in induced power for the Repeat condition relative to the Novel condition around 1100–1200 ms post-stimulus (earliest onsets detected in the beta frequency range at 1171 ms, 1150 ms, 1081 ms, and 1112 ms in the left frontal, left fusiform, dACC/pre-SMA and right fusiform regions, respectively) through approximately 2100–2300 ms post-stimulus (latest offsets in the theta frequency range at 1888 ms, 2381 ms, 2138 ms, and 2609 ms in the left frontal, left fusiform, dACC/pre-SMA and right fusiform regions, respectively; Supplementary Figs. 1 and 2; Supplementary Table 1; see bolded segments backed by light blue background in Fig. 2C for visualization effects in the gamma band, specifically). However, no significant differences in the timing of effects were identified after correction for multiple comparisons. Additional effects in the delta frequency range (1–3 Hz) were also observed in the left fusiform [early: all *t*s(39) > 3.24, *p*s < 0.0025, *q*s < 0.05], dACC/pre-SMA [late: all *t*s(39) < −2.86, *p*s < 0.0068, *q*s < 0.05], and the right fusiform regions [late: all *t*s(39) < −3.05, *p*s < 0.0042, *q*s < 0.05] (Supplementary Figs. 1 and 2; Supplemental Table 1).

**Fig. 3:**
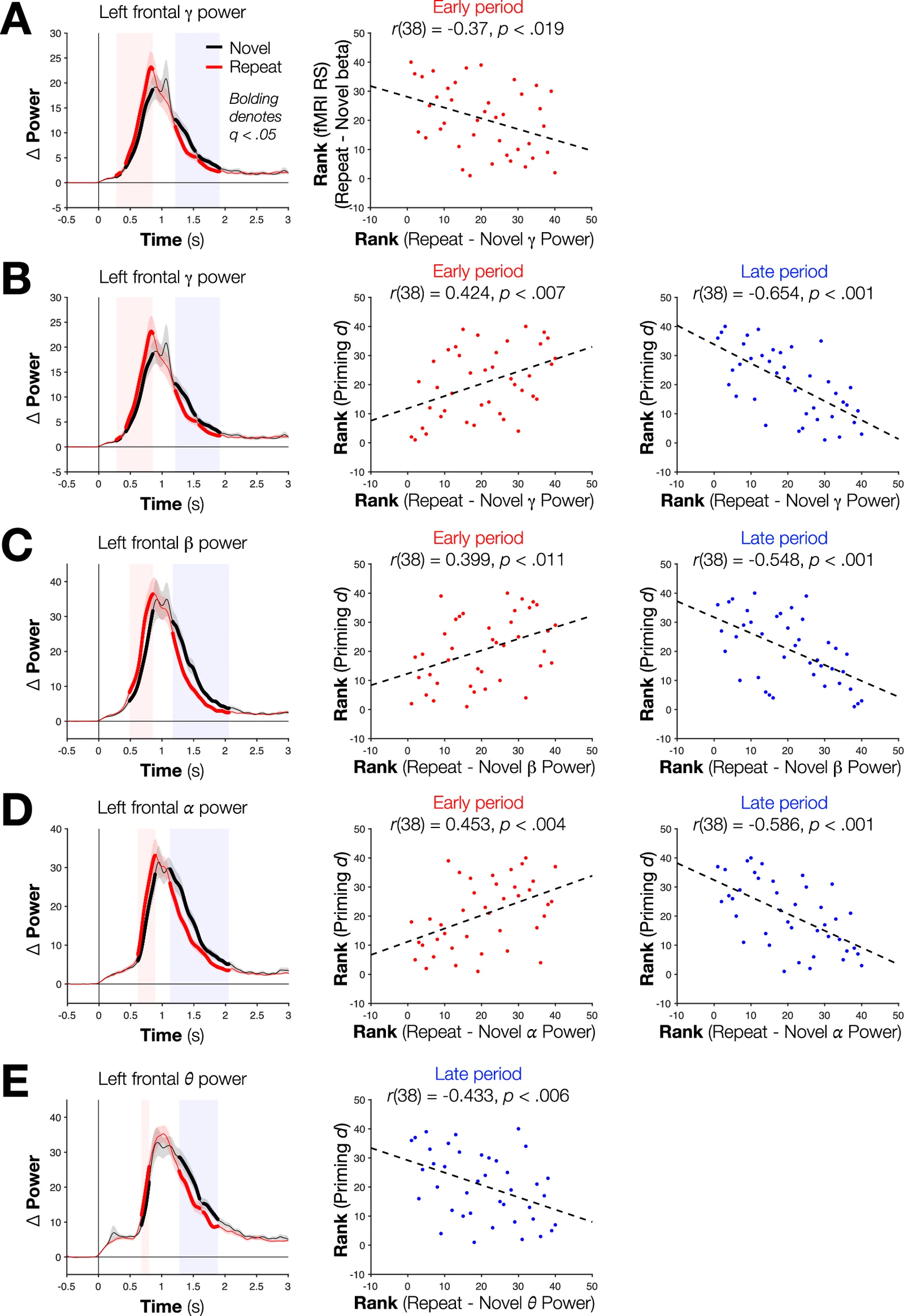
Left frontal power differences correlate across participants with fMRI repetition suppression in the same region and with behavioral priming. **A** Significant Repeat > Novel gamma power was observed in an early period, whereas significant Novel > Repeat power was observed in a late period. Across participants, EEG power differences during the early window and fMRI repetition suppression magnitudes were significantly (Spearman) correlated. **B** Within the gamma band, early periods of Repeat > Novel power differences, and later Novel > Repeat differences, both correlate (Spearman) with the magnitude of repetition priming. **C** This same pattern was observed in the beta band, **D** as well as in the alpha band. **E** “Late” window correlations were also observed in the theta band. Statistics are based on *N* = 40 independent participants. Shaded curves around timeseries data reflect *SEM*.

Having identified “early” repetition-related increases and “late” repetition-related decreases in induced EEG power, we then asked if the mean power differences during these periods correlated, across subjects, with either the magnitude of repetition priming or of BOLD RS in the four fMRI-defined regions of interest. A significant (Spearman) correlation was identified between differences in local EEG power and fMRI RS in the left frontal region for the early period (Repeat > Novel) in the gamma band [*r*(38) = −0.37, *p* < 0.0189, *q* < 0.05; Fig. 3A]. In contrast, correlations between differences in EEG power and priming magnitude across participants were observed broadly across the four regions in both the early and late periods for the gamma and beta bands [|*rs*(38)| > 0.3906, *p*s < 0.0128, *q*s < 0.05 for all] and in the late periods for the alpha and theta bands, as well [|*r*s(38)| > 0.4197, *p*s < 0.008, *q*s < 0.05 for all] (see Fig. 3B–E for left frontal correlations; see Supplementary Table 2 for all results by region and frequency band).

The pattern of power increases and decreases for Repeat versus Novel conditions shown in Figs. 2 and 3 suggested a leftward shift of the curves for the Repeat condition to earlier time points, rather than a more complex difference in overall magnitude of the curves. Importantly, such a temporal shift is predicted by the Facilitation model. To test this issue, we examined the sum of the areas under the power curves for the two conditions across participants. The curves did not significantly differ in any band or region following correction for multiple comparisons, although three uncorrected differences (Sign Test, all uncorrected *p*s < 0.039 for Novel > Repeat) were observed for beta power in the frontal and right fusiform regions and for theta power in the dACC/pre-SMA region. These results indicate that the large “early” and “late” differences between conditions predominantly reflect the shifting of the Repeat curves to earlier time points and not differences in the overall amount of power relative to a pre-stimulus baseline. This shift can also help to explain the change in signs between early and late effects—the former is due to the more rapid rise in power for Repeat items, and the latter is consistent with a more rapid fall for those same Repeat items.

In the left frontal region, the “early” power difference in the gamma frequency band was jointly correlated with both fMRI RS and priming. We therefore examined whether these effects share a common underlying pool of variance or reflect unique variances using partial correlation. The correlation between power differences and priming magnitude remained after partialling out left frontal fMRI RS magnitude [partial *r*(37) = 0.335, *p* = 0.037]. In contrast, the correlation between left frontal fMRI RS and priming magnitude—a well-replicated finding in this literature^11,13,23^—was no longer significant after partialling out power differences [partial *r*(37) = −0.24, *p* = 0.14]. EEG-induced power differences can therefore explain unique variance in priming magnitude beyond that explained by fMRI RS, but fMRI RS, in turn, fails to explain significant variance beyond what is shared with induced power differences.

### Oscillatory power differences and their correlations with behavior do not reflect the overt speech response

It was possible that the differences in power between Repeat and Novel conditions or their correlations with behavior could have been due to speech-related artifacts, such as an electromyographic (EMG) response in articulatory muscles. This possibility was investigated in several ways. First, results were compared with and without additional ICA-based EMG cleaning of the data. Although the amplitudes of the ERP waveforms were decreased by the ICA-based removal of the components during the time of the overt responses that explained the top 90% of timeseries variance, the correlations between the induced power differences and behavior marginally increased in magnitude when the cleaning was applied (*p* < 0.09, permutation test; Supplementary Figs. 3 and 4, Supplementary Table 3). Second, we examined whether correlations between power differences and priming magnitudes were strongly altered by controlling for mean response times (which are, necessarily, coupled to the speech/motor response) through partial correlation. All of the initially detected correlations remain significant when response time was controlled in this manner (see Supplementary Table 4). For example, the across-subjects correlation (Spearman) of the “early” power differences in the left frontal region with priming was *r*(38) = 0.4236, *p* = 0.0065 before and *r*(37) = 0.4239, *p* = 0.0072 after, partialling out mean response time across participants. Third, we checked whether our source-estimated power results at each node on the cortical surface were consistent with an EMG or motor response origin. Again, these analyses failed to indicate an artifactual basis to the observed results (see Supplementary Movies 1–4). We also back-projected the estimated cortical sources to the scalp to identify the most important scalp electrodes for each source region (Supplementary Fig. 5). This provided further evidence that the four cortical surface regions were not due to a common spatial artifact and permitted companion analyses on spatially distinct collections of scalp electrodes (Supplementary Figs. 6–9).

**Fig. 4:**
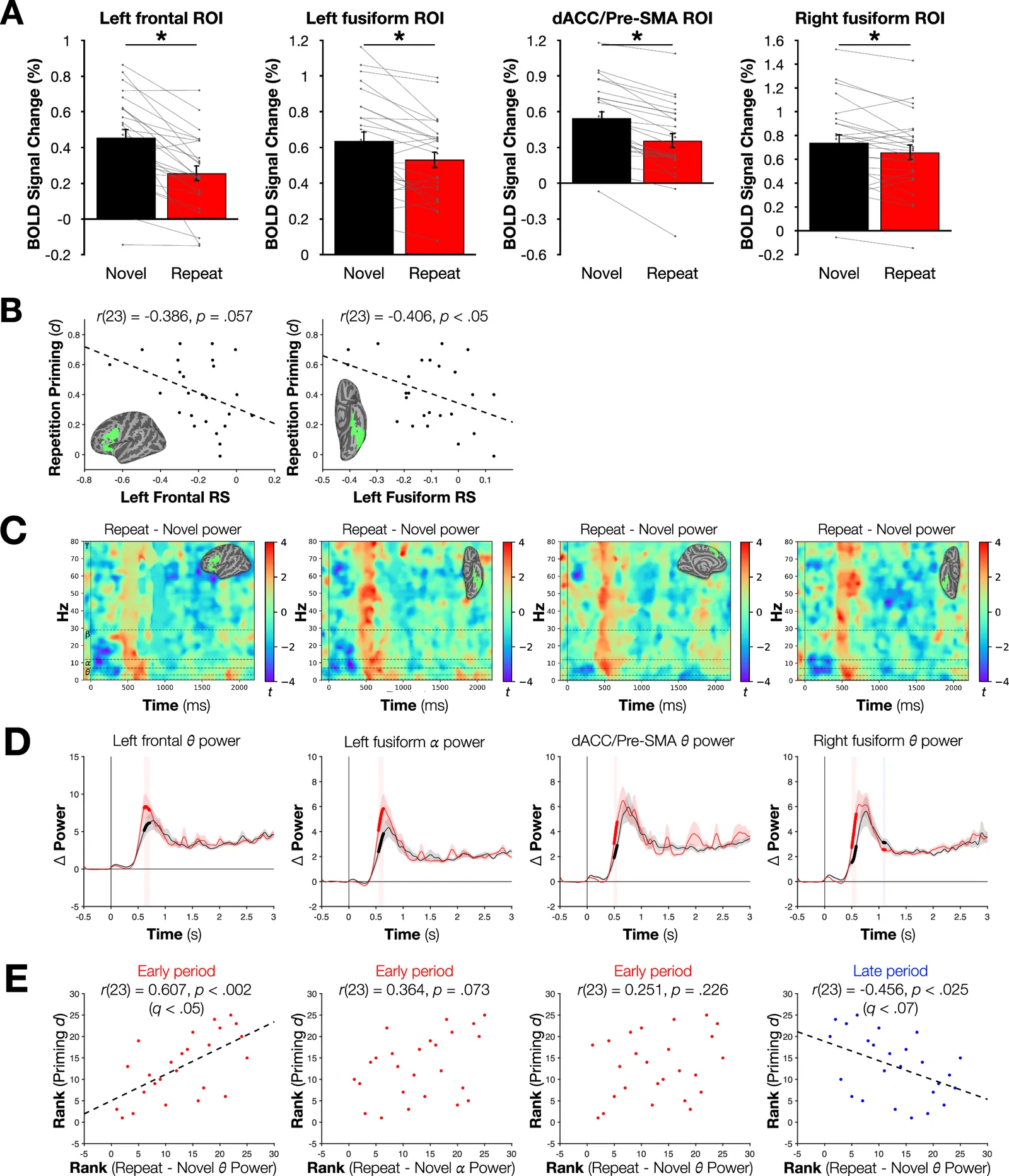
Results associating repetition priming with fMRI repetition suppression and differences in EEG power replicate when no spoken responses are required. **A** Within each of the 4 regions of interest defined in the overt naming data, significant repetition suppression effects were observed in a “covert naming” experiment. **B** The magnitude of repetition suppression effects was significantly correlated with repetition priming across subjects in the left fusiform region, with a similar but non-significant correlation observed in the left frontal cortex. **C** An early period of Repeat > Novel power was observed across frequencies in time-frequency analysis of EEG data in every region, **D** most commonly in the theta or alpha bands. **E** The magnitude of “early” Repeat > Novel theta power correlated (Spearman) significantly across participants with repetition priming in the left frontal cortex, as did “late” theta power differences in right fusiform cortex (albeit at an uncorrected level). Statistics are based on *N* = 25 independent participants. * denotes *p* ≤ 0.01. Error bars on bar plots and shaded curves around timeseries data reflect *SEM*.

Finally, we prospectively designed and conducted an additional experiment in which participants (*N* = 25) covertly named pictures during simultaneous EEG/fMRI, indicating when they had identified each object via a button press in their left (non-dominant) hand (see “Methods” section). We used the time windows of significance and fMRI regions of interest from the overt naming experiment to evaluate any power differences and correlations with priming magnitude. As expected from prior studies^23,31^, priming was somewhat attenuated during covert naming [Repeat $\bar{M}$ (SD) = 572.2 (74.9) ms, Novel $\bar{M}$ (SD) = 642.1 (107.5) ms; *t*(24) = 6.47, *p* < 0.001, *d* = 1.29]. However, fMRI RS was still observed in the same four regions of interest identified in overt naming [left frontal *t*(24) = 5.97, *p* < 0.001, left fusiform gyrus *t*(24) = 3.78, *p* < 0.001, dACC/pre-SMA *t*(24) = 8.12, *p* < 0.001, right fusiform gyrus *t*(24) = 2.78, *p* = 0.01; Fig. 4A, see also Supplementary Fig. 10]. A significant correlation between fMRI RS and priming magnitude was observed in the left fusiform region [*r*(23) = −0.405, *p* = 0.044], and a similar but non-significant correlation between fMRI RS and priming was observed in the left frontal region (*r* = −0.386, *p* = 0.057; Fig. 4B). In all four regions, time-frequency analysis identified “early” periods of greater power for Repeat than Novel stimuli in at least one frequency band (left frontal theta power: all *t*s > 2.64, *p*s < 0.0143, *q*s < 0.05; left fusiform gamma power: all *t*s > 2.55, *p*s < 0.0175, *q*s < 0.05; left fusiform alpha power: all *t*s > 2.77, *p*s < 0.0106, *q*s < 0.05; dACC/pre-SMA theta power: all *t*s > 2.96, *p*s < 0.0069, *q*s < 0.05; right fusiform theta power: all *t*s > 2.74, *p*s < 0.0115, *q*s < 0.05; Fig. 4C, D). One “late” period of greater power for Novel than Repeat stimuli was also detected for the right fusiform region in the theta frequency range (all *t*s < −2.74, *p*s < 0.0115, *q*s < 0.05; Fig. 4D, rightmost column). While effects were not equally detected in all frequencies, the overall pattern of time-frequency power differences appears qualitatively consistent with those seen in overt naming, with relatively broadband early and late windows at approximately the same time periods.

As in the overt naming experiment, we tested whether the corrected differences in induced power between the Repeat and Novel conditions were correlated with fMRI RS and priming. The early difference in theta frequencies in the left frontal region was significantly correlated (Spearman) with priming magnitude [*r*(23) = 0.607, *p* < 0.0014, *q* < 0.05], and the late difference in theta frequencies in the right fusiform region was correlated with priming magnitude at an uncorrected level [*r*(23) = −0.456, *p* < 0.0219, *q* < 0.07] (Fig. 4E). No significant associations with fMRI RS were detected. Taken together, these findings demonstrate that the early and late power differences seen in overt naming are also observed in covert naming and therefore cannot be due to overt speech artifacts. Furthermore, priming is also associated with induced power differences in covert naming, although with the largest effects seen in somewhat lower frequencies (theta) than in overt naming (most robust in gamma/beta). Future work can clarify the nature of this observed difference.

### Within-participants, items previously identified as “Strongly” or “Weakly” primeable show distinct correlation patterns with oscillatory power differences

The covert naming data ruled out an artifactual basis for the observed association between induced power and priming, while highlighting the consistency of the “early” window EEG power differences in this link. We therefore conducted a further set of analyses, focusing on within-participant priming and its association with induced EEG power differences during the early period to complement the across-participant analyses described previously. Stimuli were first split into equally-sized groups (Strongly primeable, Weakly primeable) based on the average magnitude of priming observed in a prior study with a different group of participants^23^. After ensuring that the independent estimate of primeability was replicated in the current participants, correlations between priming and induced power differences were then examined separately for Strongly and Weakly primeable stimuli. Based on the results depicted thus far in this report, one could expect greater power differences for Strongly than Weakly primeable items, and that Strongly primeable items should show a larger EEG-behavior correlation.

**Fig. 5:**
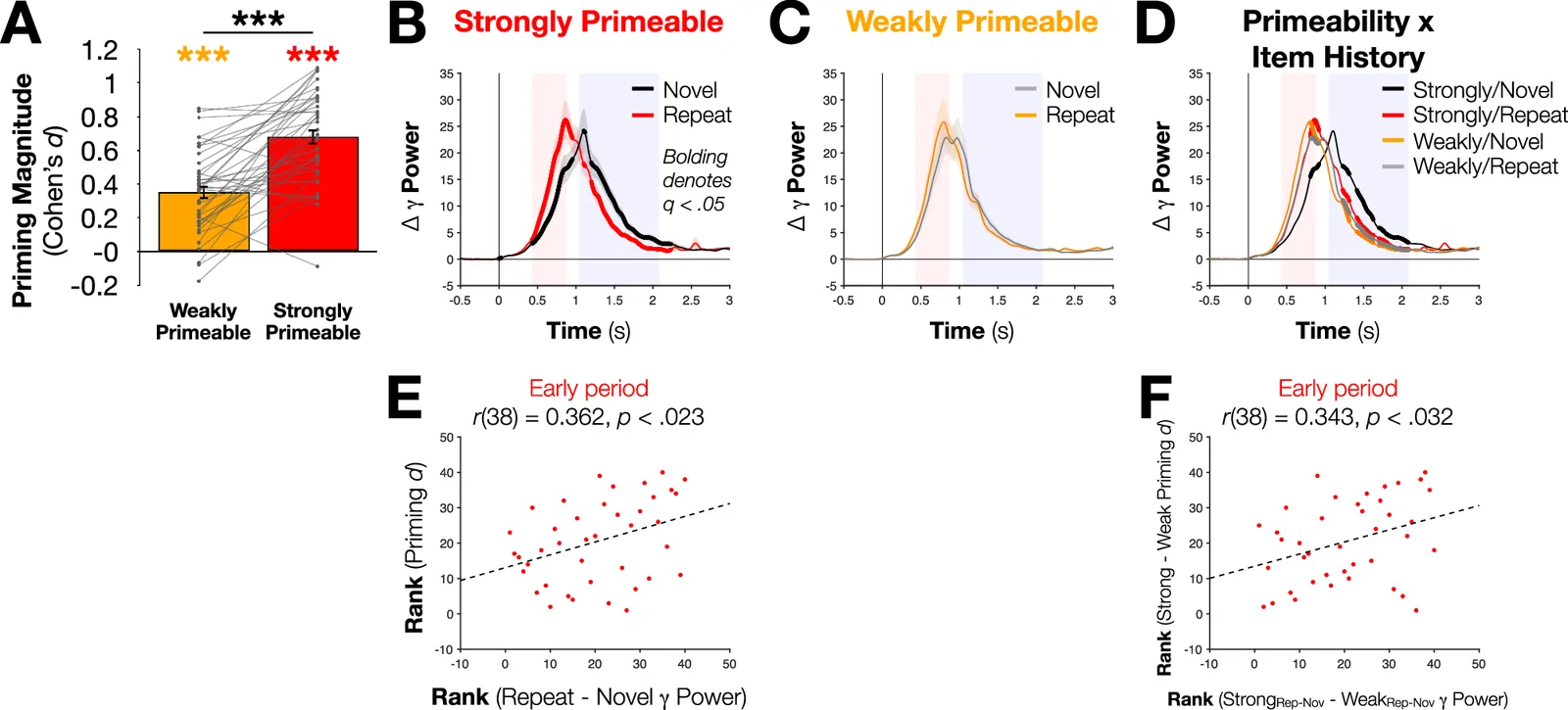
Results of within-participant comparisons of priming magnitude and their association with induced gamma-band EEG power differences in the left fusiform cortex. **A** Stimuli were separated into Strongly and Weakly primeable groups (100 items each) based on average primeability in an earlier study^23^. Although both levels of primeability were associated with significant repetition priming, Strongly primeable items were associated with significantly greater effect sizes. **B** Strongly primeable items were associated with induced gamma EEG power differences, but **C** Weakly primeable items did not show significant differences. Red and blue background colors reflect periods of significant Novel and Repeat power differences corresponding to those in Fig. 2C. **D** The interaction between Novel and Repeat item history for Strongly and Weakly primeable items was quantified, with significant effects emerging in early and late windows. **E** Subsequent analyses revealed that, across participants, a significant correlation was observed between priming magnitude and induced gamma power differences for Strongly primeable items. **F** The behavioral difference in priming magnitude between Strongly and Weakly primeable items also correlated, across participants, with the Repeat-Novel power differences in the Strongly versus the Weakly primeable conditions. Statistics are based on *N* = 40 independent participants. *** depicts *p* < 0.001. Colored asterisks reflect the results of one-sample *t*-tests, whereas the black asterisks reflect a paired-samples *t*-test. All *t*-tests were two-tailed. Error bars on bar plots and shaded curves around timeseries data reflect *SEM*.

One-sample tests of the within-participant effect sizes for repetition priming (Cohen’s *d*) confirmed that both categories elicited significant priming [Strongly primeable $\bar{M}$ (SD) = 0.679 (0.275), *t*(39) = 15.63, *p* < 0.001; Weakly primeable $\bar{M}$ (SD) = 0.351 (0.249), *t*(39) = 8.90, *p* < 0.001; Fig. 5A]. A direct comparison found that Strongly primeable items had larger within-participant priming effect sizes on average than did the Weakly primeable items [*t*(39) = 6.68, *p* < 0.001, *d* = 1.06].

Having demonstrated behavioral differences in the Strongly and Weakly primeable items, we then examined differences in induced EEG power between Novel and Repeat items, separately, in the Strongly and Weakly primeable conditions in the four fMRI-defined ROIs. Interactions between item history (Novel vs. Repeat) and Primeability (Strongly vs. Weakly primeable) were also calculated to inform the interpretation of the direct comparisons. In the left fusiform cortex, induced gamma power differences between Novel and Repeat stimuli were observed in early and late periods for Strongly Primeable, but not Weakly primeable, stimuli, and the interaction of these factors was significant (Fig. 5B–D). A significant correlation was observed across participants between priming magnitude and induced gamma power differences for Strongly primeable items [*r*(38) = 0.362, *p* < 0.023; Fig. 5E]. Furthermore, and uniquely across all investigated ROIs, the within-subject difference in behavioral priming magnitudes (Strongly versus Weakly primeable) correlated with the corresponding induced gamma power differences in EEG (Repeat-Novel Power in Strongly versus Weakly primeable items, a metric of the strength of the interaction) [*r*(38) = 0.343, *p* < 0.032; Fig. 5F]. Interactions were also assessed in other regions and frequencies (Supplementary Figs 11–18). A significant interaction of item history and Primeability was also observed during the early period for the left frontal ROI in alpha frequencies, and in the dACC/pre-SMA ROI for gamma and beta frequencies. These results indicate both significant across- and within-participant correlates of priming for induced power differences in the left frontal, left fusiform, and dACC/pre-SMA ROIs for at least some frequencies in each. However, the most internally consistent and extensive results were observed for the gamma frequency range in the Left Fusiform ROI.

## Discussion

In summary, we have found that repetition-related improvements in behavior (priming) and decreases in neural activity (repetition suppression) during object identification are accompanied by more rapid increases (and subsequent decreases) in oscillatory power in the repeated condition in concurrently acquired EEG. Power changes were robustly correlated with the magnitude of priming across participants, overlapping in variance explained with fMRI RS, while also explaining unique additional variance. Results, particularly in the left frontal cortex, were qualitatively replicated when using a covert naming task, ruling out an artifactual basis of the power and priming relationships and lending support to their robustness.

Our results suggest that priming may be related to changes in neural activity through a novel combination of the Synchrony and Facilitation models. The Synchrony model predicts enhanced population-level oscillatory power when processing repeated items, as was observed here in the “early” period. Facilitation predicts more rapid onsets and offsets, although the current data diverge from earlier articulations of Facilitation^18,19^ insofar as the differences were observed in the domain of oscillatory power rather than total neural activity per se. However, Facilitation also provides a means of accounting for both early and late periods of repetition-related differences in activity: given an earlier rise in and resolution of processing Repeat items, one would expect an early period of Repeat > Novel power differences due to the leftward shift of the power curve (Fig. 2). This same shift would then produce a later period in which an opposite pattern would necessarily emerge as the processing of Novel items is slower to resolve. It is therefore the early period that seems most relevant for understanding the relation between repetition priming and repetition suppression. Notably, although we have documented these changes across all four of the fMRI-defined regions exhibiting RS, prior work has highlighted a particularly critical role of left frontal cortex in mediating both repetition priming and repetition suppression across changes in task and stimulus form, implicating response learning mechanisms^13,15,35^. We do not perceive any incompatibility between these proposals and our current results. Indeed, one view is that this novel combination of Synchrony and Facilitation models reflects stimulus-to-response mapping processes.

Consistent with the present findings, prior EEG studies with repeated pictures have shown differences in induced gamma power^28,29^, although these have often utilized much shorter-term repetitions, sometimes without a requirement for overt responses to the stimuli of interest^28^. One prior study^30^ notably identified increased induced gamma power for well-known objects relative to novel objects in addition to the EEG RS effects that we also show, replicating this effect across two different lab settings. Similar results for increased gamma power and coherence with stimulus repetition have also been observed with microelectrode recordings in monkey V1 and V4 simultaneously with overall decreases in firing rate to grating and natural object stimuli^24–27^. Repetition-induced decreases in firing rate that coincide with increases in synchronous activity have even been observed in insects such as locusts^36^ and Drosophila^37^, suggesting a relatively general mechanism of neural processing efficiency that has been preserved across species.

The pattern of results seen in overt picture naming was replicated qualitatively in covert naming for “early” (Repeat > Novel power in all four regions showing fMRI RS) and “late” periods (Novel > Repeat power in the right fusiform region). We further detected a correlation between early power differences in the left frontal region and priming magnitude, with an uncorrected correlation for the late period in the right fusiform region. While the early/late pattern appeared to be relatively broadband, the correlations with priming were observed in theta rather than gamma frequencies. These differences may be due at least in part to the smaller effect sizes typically seen in covert naming^23^, as well as the smaller sample size utilized in the covert experiment (covert *N* = 25; overt *N* = 40). The covert naming experiment was designed primarily to evaluate the continued presence of the early/late power difference pattern in the absence of overt speech rather than to replicate the correlations with behavior, and future studies with this and other tasks should be examined with larger samples to assess any real task-specific differences in the neural correlates of priming.

Results from across-participant analyses in overt and covert naming datasets combined to suggest that the early period may be particularly fruitful to explore in more detail, and comparing priming magnitudes within-participants indeed found that this was the case. Here, support for within-participant correlates of priming was observed in the left frontal, left fusiform, and dACC/pre-SMA regions, although the left fusiform cortex especially stood out. This was because it exhibited both a significant interaction of primeability (Strongly/Weakly primeable) and item history (Novel/Repeat presentation) in its induced gamma-band power, and the magnitude of this interaction was also correlated with behavior across participants—indicating joint across-participant and within-participant correlates. It is notable that the left fusiform (along with left frontal cortex and dACC/Pre-SMA) emerged in earlier fMRI-based work that utilized a similar overt naming approach, as being correlated with priming effects at the within-participant level^23^. Taken together, these results suggest that the left fusiform gyrus should be strongly considered along with the left frontal cortex as an important neural locus contributing to priming.

Thus far, our discussion has emphasized the support of the current results for the Synchrony and Facilitation models. This should not, however, be taken to imply that the EEG/fMRI data presented here should be viewed as evidence *against* the Sharpening or Predictive Coding models. Rather, the current data, and especially the EEG component, cannot provide information about firing rate distribution differences between novel and repeat items that we view as critical for a direct test of the Sharpening account. On the other hand, tests of predictive coding require estimation of directional connectivity, and the volumetric spread of the EEG signal renders strong tests of this model difficult in the current dataset. Future work, focusing directly on Sharpening or Predictive Coding, would complement that presented here. In addition, although the power differences reported here are consistent with predictions of the Synchrony model, differences in aperiodic properties of the power spectrum across task conditions could, in principle, also contribute to such observations. Future work that is optimized for assessing cross-regional coupling while separately modeling oscillatory and aperiodic activity^38–40^ would directly address this possibility.

By linking the activity states predicted by the Synchrony and Facilitation models to the magnitude of both RS and priming, our findings provide a mechanistic explanation for the long-standing puzzle of how reduced neural activity can support behavioral improvements rather than behavioral impairments—a relationship that has been observed across humans, monkeys, and rodents. While the population-level measures of neural activity that are utilized in non-invasive human neuroimaging cannot yield direct evidence of the synchronization of neural firing, the family of effects shown using the current multimodal approach is consistent with earlier, enhanced neural synchronization, and it provides an important target for future work using more direct neural recording techniques in animals and human patients.

## Methods

### Participants

#### Overt naming

A total of 45 participants were recruited from the National Institutes of Health and the surrounding area. Five of these participants were excluded (1 following detection of a neurological abnormality, 1 for excessive motion in 75% of task scans, 2 due to equipment malfunctions, and 1 because their vision could not be corrected with MR-compatible lenses). The remaining 40 participants (18 male) had a mean age of 24.4 ± 2.9 years (range: 19–32). Participants had a mean of 16.2 ± 0.97 years of education (range: 14–19), were right-handed, had normal or corrected-to-normal vision, reported no history of psychiatric or neurologic illness, and were native speakers of English. Informed consent was obtained from all participants, and the experiment was approved by the NIH Institutional Review Board (Protocol 93-M-0170; clinical trials number NCT00001360). All ethical regulations relevant to human research participants were followed. Participants received monetary compensation for their participation.

#### Covert naming

Twenty-six participants were recruited from the National Institutes of Health and the surrounding area. One participant was excluded when sufficiently good contact could not be made between the EEG sensors and their scalp. The remaining 25 participants (10 male) had a mean age of 26.4 ± 4.3 years (range: 21–35) and had completed 16.6 ± 1.15 years of education (range: 14–19). Participants were right-handed, had normal or corrected-to-normal vision, reported no history of psychiatric or neurologic illness, and were native speakers of English. Informed consent was obtained from all participants, and the experiment was approved by the NIH Institutional Review Board (Protocol 93-M-0170; clinical trials number NCT00001360). All ethical regulations relevant to human research participants were followed. Participants received monetary compensation for their participation.

### Experimental stimuli

Stimuli consisted of 200 colored photographic images of animals, plants, and everyday objects, and have been used in prior laboratory studies (e.g., refs. ^23,32^). Images were split into 2 groups (lists) of 100 images each, which were matched in average lexical properties including log HAL frequency (means = 8.54 vs. 8.60, *t*(198) = .293, *p* = 0.770), name length (5.55 vs. 5.93 letters, *t*(198) = 1.31, *p* = 0.191), and average naming time based on prior studies in the laboratory using a similar paradigm (824.8 ms vs. 831.8 ms, *t*(198) = 0.496, *p* = 0.621). Experimental task scripts were written in PsychoPy2 software. In-scanner stimulus presentation was accomplished using a desktop running Windows 10 connected to a 120 Hz, 1920 × 1080 pixel MR-compatible monitor. Images subtended approximately 5° of visual angle and were centrally presented against a dark gray background. Participants viewed the monitor through a mirror attached to the head coil. A central white fixation cross was shown between stimulus presentations. Out-of-scanner stimulus presentation was conducted using a MacBook Pro laptop (Apple, Cupertino, CA) in a behavioral testing room.

### Task design

#### Pre-scan object naming

Prior to scanning and EEG cap placement, participants in both overt and covert naming tasks viewed and overtly named 100 images twice in a testing room. Images were from one of the predetermined lists described previously, and the list used during this portion of the task was counterbalanced across participants. All images were presented a single time before any were re-presented. 500 ms prior to each image presentation, the white fixation cross changed to an orange color to serve as an onset preparation cue. Images were then presented for 300 ms and followed by a white fixation cross for 1700 ms. Participants were instructed to name aloud each image as quickly, accurately, and clearly as possible, and their responses were logged by an experimenter. A schematic of this task structure is presented in Fig. 1A.

Following the pre-scan object naming phase, participants were fitted with an MRI-compatible EEG cap. Electrode locations and impedances were then recorded and adjusted as necessary (described in more detail under the “EEG data acquisition” section), and participants were then led into the scanner to complete the in-scanner object naming task. The average delay between the end of the pre-scan object naming period and the beginning of in-scanner object naming was approximately 75 min.

#### In-scanner object naming

Participants underwent simultaneous EEG/fMRI scanning while again naming objects. 200 items were now presented in total: 100 were repeated from the pre-scan naming phase (Repeat), and 100 were newly presented images (Novel). Repeat and Novel images were intermixed throughout each scan run. The basic trial structure remained similar but was modified to fit a slow event-related fMRI design with widely spaced trials (Fig. 1B). 1000 ms before each image appeared, the fixation cross changed to an orange color to serve as an onset preparation cue. Images were then presented for 300 ms and followed by a white fixation cross for a variable period of 5150–11600 ms (in steps of the TR = 2150 ms). The order of stimuli and the delay period following each stimulus were pseudorandomized to maximize design efficiency^41^. Overt naming participants were instructed to name aloud each image as quickly, accurately, and clearly as possible. Responses were recorded for subsequent scoring and response time (RT) calculation (see the “Behavioral data acquisition” section). Covert naming participants did not speak words aloud but instead pressed a button with their non-dominant hand (left) to indicate the beginning of their naming response (as in refs. ^23,31^).

#### Post-scan object naming (covert naming participants only)

Participants completing the covert naming task in the scanner also completed an additional post-scan session in which they named all 200 pictures immediately after completion of fMRI scanning and the removal of the EEG net. This allowed for overt naming of all types of stimuli, and any stimuli that were not successfully named during the post-scan session had their associated in-scanner trials marked as incorrect.

### Data acquisition

#### fMRI data acquisition

Scanning was conducted on a General Electric Discovery MR750 3.0 T scanner, using a 32-channel head coil. Separate high-resolution T1 structural images were obtained for each participant, both with the EEG cap and without it (TE = 3.47 ms, TR = 2.53 s, TI = 900 ms, flip angle = 7°, 172 slices with 1 × 1 × 1 mm voxels). For 19 subjects, T1 scans without the cap were not collected on the day of scanning but were instead obtained in separate sessions. Functional images were acquired using a BOLD-contrast-sensitive multi-echo echo-planar sequence (Array Spatial Sensitivity Encoding Technique or ASSET acceleration factor = 2; TEs = 13.5, 34.2, 54.8, and 75.5 ms; TR = 2150 ms, flip angle = 75°; 72 × 72 matrix, in-plane resolution = 3.0 mm). Whole-brain EPI volumes (MR frames) consisted of 42 interleaved, 3.0 mm-thick oblique axial slices, aligned manually to the AC-PC axis. One additional EPI scan, with identical parameters but an inverted phase encoding direction (P-A), was collected prior to task scans for purposes of distortion correction during preprocessing. Foam pillows helped stabilize head position for all participants, and foam earplugs attenuated scanner noise. A sensor was placed on each participant’s left middle finger to record heart rate, and a respiration belt monitored breathing for each participant.

#### EEG data acquisition

EEG data were collected using a 256-electrode (Ag/AgCl) HydroCel GSN 220MR Geodesic Sensor Net (Magstim EGI, Plymouth, MN, USA). Three different-sized nets were available to accommodate different head sizes, but all were structured identically. Participants' head circumferences were measured to identify the appropriate net size. Prior to application, each net was soaked in warm water containing a combination of potassium chloride and baby shampoo. Placement indicators were marked on each participant’s head with a watercolor pencil to signify the Cz point. Following placement of the net, the specific positions of each electrode were recorded using a handheld EGI GeoScan stereo camera and its accompanying software. Impedances were measured using EGI NetStation v5.4.2, and experimenters made adjustments until electrical impedances were below 40 kΩ. Once completed, a tubular elastic net was placed over the EEG net to stabilize the electrodes prior to participants entering the scanner.

EEG signals from all channels were digitized at a 1000 Hz sampling rate with a single Net Amps 400 amplifier and recorded onto a MacBook Pro laptop (Apple Inc., Cupertino, CA) running EGI NetStation. Data were collected unfiltered. Horizontal eye movements (i.e., saccades) and blinks were monitored using built-in electrodes on the sensor net^42^. EEG data were recorded using a Cz reference. For 20 overt naming participants, two additional sensors were placed on the chest for collecting EKG data (hardware to allow this for the first 20 participants was not available due to COVID-related supply chain disruptions). EKG was recorded with the same parameters as the EEG signal (unfiltered, digitized at 1000 Hz).

The EEG system clock was synchronized with and slaved to the MRI scanner clock (10 MHz polling rate), and synchronization was further improved by the collection of sync pulses that were sent from the scanner at the start of each TR. Orange fixation cross and object picture onset times were sent from the stimulus PC to the NetStation recording computer using PsychoPy2’s *egi* package (https://psychopy.org/api/hardware/egi.html). A 37 ms asynchrony (average delay) was measured with a photodiode between picture onsets on the monitor and the signal sent to NetStation; this was subsequently accounted for in EEG data analyses.

#### Behavioral data acquisition

Participants spoke all responses into a FOMRI-III NC MR-compatible microphone (OptoAcoustics Ltd., Mazor, Israel). Audio signals from this microphone were routed into an M-Audio FastTrack Ultra 8-R USB audio interface, which in turn was connected to a Dell Precision M4400 laptop. Responses were recorded using Adobe Audition. In conjunction with the spoken audio recording, the stimulus presentation computer sent out a square wave pulse at the onset of each stimulus that was captured on a parallel audio track by the recording laptop. Onset time differences between the square pulse onset and the voice response in each trial were used to calculate voice RTs^23,32^. Naming responses were recorded by the experimenter and subsequently marked as correct/incorrect.

### Data preprocessing

#### MRI data preprocessing

MRI data were preprocessed using AFNI^43^, FreeSurfer^44^ v7.2, and SUMA^45^. Dicoms were converted to 3- (for structural scans) or 4-dimensional (for functional scans) files using *dcm2niix_afni*. T1 structural images were then processed using FreeSurfer’s *recon-all* tool, skull-stripped using *@SSwarper*, and standard SUMA std.141 surface meshes for each participant were generated using *@SUMA_Make_Spec_FS*. The first 4 MR frames of each EPI scan run were excluded to allow for T1 equilibration. Blip-up/blip-down distortion correction was carried out using AFNI’s *unWarpEPI.py* script for each run using the scan run with an inverted phase encoding direction. EPI data were realigned to the first volume of each scan run via rigid body transformation and subsequently registered to the anatomical image. Motion was calculated using the second echo of each run, and motion estimates were retained for use as nuisance regressors during subsequent general linear model (GLM) analysis. Functional data then underwent multi-echo ICA analysis using *tedana.py* software^46,47^. This procedure initially calculates a weighted average of the different echo times within each voxel and then uses spatial ICA and the known properties of T2* signal decay over time (i.e., echoes) to separate and reject putatively artefactual components, such as thermal noise or head motion, while retaining components consistent with the Blood Oxygen Level Dependent (BOLD) response. Denoised timeseries data were mapped to each participant’s std.141 surface mesh, and a geodesic blur along the unfolded cortical surface was applied to achieve an FWHM of 8 mm. Data were then normalized and rescaled to percent signal change.

All task scan runs consisted of 237 MR frames (233 after initial frame discarding) and were approximately 8 min, 30 s in length. Four naming scans were completed for each participant. Average run-level motion estimates were derived using AFNI’s *@1dDiffMag* based on three translational and three rotational motion parameters; runs with values > 0.4 mm/TR were excluded. For the final overt naming sample, 2 participants had 2 runs excluded based on this requirement. For the covert naming sample, a single participant had 2 runs excluded for excessive motion. For all retained runs, single TRs were censored if frame-to-frame motion exceeded a Euclidian Norm of 0.3 mm or if ≥10% of voxels in that TR were flagged as outliers by AFNI’s *3dToutcount*. This resulted in an average of 6 ± 2% of TRs being censored for overt naming participants and 5 ± 1% for the covert naming participants.

#### EEG data preprocessing

EEG data were initially processed to remove MR-induced gradient artifacts and Ballistocardiogram (BCG) artifacts using standard NetStation functions. First, MR-induced gradient artifacts were removed from the EEG using an average artifact subtraction^48^ based on a sliding window of 10 TR pulse events. BCG artifacts were then removed in a subsequent step. For the participants for whom EKG sensors were not available, BCG effects were estimated from the EEG data using NetStation’s built-in BCG identification algorithm; for subjects for whom EKG was collected, QRS complexes of the heartbeat were defined in the EKG channel. In both cases, BCG removal was accomplished using an optimal basis set approach^49^.

Two pipelines were used to pre-process EEG data following removal of MR gradient and BCG artifacts (Supplementary Fig. 4A). Both interpolate bad channels and attenuate electrooculographic (EOG) and tongue-related electromyographic (EMG) artifacts. The second pipeline includes an extra step to attenuate speech-related EMG. Both pipelines were fully conducted using MNE-Python version 1.8 (stable)^50^.

#### Bandpass filtering and handling of bad channels

EEG data were re-referenced to the Cz channel (referred to as “VREF” by the EGI software) and bandpass-filtered from 0.1 to 100 Hz using *mne.Raw.filter* with default arguments. Next, the 60 Hz frequency band and its integer multiples were notched out using “mne.filter.notch filter” with default arguments, except for the phase argument, which was set to “zero-double”. The filtered data were then epoched (broken down into trials) ranging from −3 to 5 s relative to stimulus onset time. Finally, each individual trial was screened for “bad channels”, that is, channels with peak-to-peak amplitude > median + 1.5 interquartile range. Bad channels were interpolated using *mne.Epochs.interpolate bads* with default arguments.

#### Attenuation of EOG and tongue EMG

EEG activity that resulted from eye, eyelid and tongue movements was removed from each channel of each trial by regressing out activity measured at electrodes at the left and right superior orbicularis oculi (in the EGI-256 montage, channels E37 and E18, respectively), left and right inferior orbicularis oculi (E241 and E238) and those at the outer cantus left and right (E252 and E226)^42^. Activity in these channels was regressed out using *mne.preprocessing.EOGRegression* with the argument “picks_artifact” set to these electrodes. The outputted weights were then used (*weights.apply*) to generate the denoised EEG. To avoid regression overfitting, the coefficients of determination (*R*^2^’s) of all regressions/channels were inspected. Channels with an *R*^2^ < 0 were treated as bad channels and interpolated using *mne.Epochs.interpolate bads* with default arguments.

#### Attenuation of speech EMG

An additional denoising step was also applied to each channel for each trial. The purpose was to mitigate putative EMG artifacts due to contraction of articulatory muscles during speech preparation and production. First, for a given trial, a time window of −0.2 to 0.2 s was centered at the RT, and the corresponding electrode data were fed to Independent Component Analysis (ICA), yielding speech independent components (ICs). This was accomplished using *mne.preprocessing.ICA*, specifying “method” = picard and “n components” = 0.9. Note that, in MNE-Python, ICA is always preceded by Principal Component Analysis (PCA). By setting “n components” = 0.9, the algorithm constrained ICA to operate on the minimum number of principal components that spanned 90% of the variance. Next, *mne.preprocessing.EOGRegression* was applied using the argument “picks_artifact” set to the timeseries of speech ICs. The outputted weights were then used (*weights.apply*) to generate additionally denoised timeseries data. Note that while the ICA was conducted on the period around the RT (−0.2 to +0.2 s), the weights were obtained from and applied to a window from 0.5 s prior to the RT up until the RT, a period of the trial considered representative of speech preparation and production. As with attenuation of EOG and tongue EMG, regression overfitting was avoided by treating channels with an *R*^2^ < 0 as bad channels and interpolating using *mne.Epochs.interpolate bads* with default arguments.

### Data analysis

#### Statistics and reproducibility

Specific information on the statistical tests performed in this study is provided in the subsections below. Unless otherwise specified, the overt naming data used a full *N* = 40, and those for the covert naming data used an *N* = 25 (the latter of which served as a replication for the overt naming data). Behavioral analyses included paired-samples *t*-tests for within-subjects/repeated measures and Pearson or Spearman correlation analyses that involved brain-behavior measures. Group-level analyses of fMRI data were conducted using one-sample *t*-tests (vs. zero) for basic activation analyses and paired-samples *t*-tests for comparisons across conditions. Analyses of EEG data used *t*-tests, correlations, and ANOVAs, as described in more detail below.

#### Behavioral analysis

Repetition priming was defined as the mean of the naming response times (overt naming) or the mean of the button press times (covert naming) for the Novel condition minus the mean of the response times for the Repeat condition. Only correct trials were used for these calculations. Repetition priming magnitudes were then estimated for each participant using Cohen’s *d* (effect size) in order to adjust for different response time ranges for each participant (see refs. ^14,23^ for discussion). Accuracy (percentage of correct responses) in the Novel and Repeat conditions was also calculated for each participant. Statistical analyses were then carried out across participants using paired *t*-tests for accuracy and repetition priming in both overt naming and covert naming. Analyses were conducted using SPSS (29.0.2) and MATLAB (2021b).

#### fMRI data analysis

fMRI data were analyzed using a GLM approach. Responses for each condition were modeled using TENT functions, which are analogous to SPM’s finite impulse response (FIR) model. This approach assumes that all responses for a given condition share the same response shape but makes no assumption as to what the shape of that response might be. Ten time points, extending from 0 s to 19.35 s in steps of the TR (2.15 s), modeled each of 3 conditions of interest. These were Repeat trials (for images named during the pre-scan period as well as during fMRI scanning), Novel trials (images named only during the fMRI scanning period), and Error trials. Error trials included multiple types of errors, including non-responses, incorrect responses, responses for which responses changed between the pre-scan and in-scanner naming periods (Repeat trials only), or trials in which responses were provided after a 2150 ms (1 TR) delay (as in ref. ^23^). For the purposes of statistical testing, response magnitudes were estimated for each condition by averaging the 3rd and 4th time points of the TENT function (reflecting the expected peak of the BOLD response, 4.3–8.6 s following stimulus onset).

Two vertex-wise contrast maps were generated from the fMRI data. First, a contrast (paired-samples, two-tailed) of the regression coefficients (beta weights) for the Repeat-Novel conditions identified vertices for which significant repetition suppression (Repeat < Novel) or repetition enhancement (Repeat > Novel) effects were observed. Correction for vertex-wise multiple comparisons was accomplished using the *SurfClust* function in AFNI, which uses the smoothness of the data to generate null surface maps and empirically defines a minimum cluster size to achieve a given whole-brain correction at a given significance level. A vertex-wise threshold of *p* < 0.001 and a minimum cluster extent of 195 vertices achieved a corrected *p* < 0.05 in this “Repetition-related changes” map.

A second contrast (one-sample, two-tailed) was conducted to identify vertices generally activated by the viewing and naming of picture stimuli, starting from the moment of image onset (irrespective of sensitivity to repetition-related effects). For each vertex, the beta weights for Repeat and Novel conditions were first averaged and then tested vs. a baseline of 0. Significant vertices (FDR corrected to *q* < 0.05) were included in this “Naming-related activation” map.

The Naming-related activation map was then used as an inclusive mask to identify cortical regions that were both activated by naming and exhibited significant repetition-related changes in activity. Regions of interest (ROIs) were identified in the left frontal cortex, the left and right fusiform cortex, and the dACC/pre-SMA. Prior work using this paradigm and approach to fMRI data analysis^23^ identified clusters in these regions, and the largest clusters in the current masked data that corresponded to each location were manually identified and carried forward for further analysis. The magnitude of repetition suppression in these ROIs was then calculated by averaging the Repeat-Novel beta weights across all vertices associated with each region, respectively, for each participant.

#### EEG scalp analysis

Following prior findings of repetition-related EEG effects over posterior locations^30^, our scalp analyses focused on posterior electrodes. In the EGI montage, these consisted of electrodes E108, E109, E115, E116, E117, E123, E124, E125, E138, E139, E140, E149, E150, E158, E159, E87, E99, E110, E118, E126, E100, E119, E127, E101, E128, E129, E141, and E153. Repetition suppression was defined as the ERP difference between Novel vs. Repeat conditions. Posterior ERPs were calculated as an average timeseries across trial epochs (−0.5 to 3.0 s relative to stimulus onset) at the selected electrodes, with a separate ERP calculated for each condition (Novel, Repeat) and participant. The mean of the baseline period (−0.5 to 0 s relative to stimulus onset) was subtracted from each trial epoch prior to averaging. The mean voltage of the Novel – Repeat ERP waveforms was compared with paired *t*-tests across participants at each time sample, with multiple comparisons controlled by False Discovery Rate (FDR, *q* < 0.05). The time period used for FDR correction was 0.0–0.6 s to match the analysis window of prior related work^30^.

#### EEG source analysis

##### Forward solution

For each participant, we used FreeSurfer’s recon-all to derive 3-D surfaces from that participant’s T1 MRI images. Next, we used mne.setup source space with the spacing argument set to “oct6” to define a source mesh over the participant’s white matter, with sources spaced by 4.9 mm. To model current conduction, we set up boundary element models (BEMs) with 5120 dipoles per hemisphere. We first ran mne.make bem model with argument ico = 4 and default conductivity arguments to generate the surfaces used by mne.make bem solution. This function was run on the brain, inner skull and outer skull surfaces output by FreeSurfer’s mri watershed segmentation utility. The resulting BEM was used when generating each participant’s forward model. We obtained the forward model using mne.make forward solution. We set the head-fMRI transform argument “trans” to “fsaverage” to calculate the transformation between the EEG montage sensors and MNE’s fsaverage coordinates.

##### Inverse solution

Inverse solutions were obtained separately for Repeat and Novel epochs. All epochs were first baseline-corrected by subtracting the mean channel data of the baseline period (*t* = −0.5 s to *t* = 0 s from stimulus presentation) from all channels over the entire epoch duration, epoch-by-epoch. Each participant’s inverse operator was set up using a Minimum Norm Estimate (MNE) model given the forward model and with source orientation constrained to be orthogonal to the cortical mantle (*mne.minimum norm.make inverse operator*, setting the argument “fixed” = True). Next, inverse solutions (or source estimates) were calculated for each epoch. This required the computation of a noise covariance matrix, which was calculated from the baseline segments of all epochs using *mne.compute covariance* with the argument “keep sample mean” = False. The function *mne.SourceEstimate.estimate_snr* was used to estimate the regularization parameter “lambda” for each participant in the overt naming experiment, averaging the peak estimates across participants. The inverse operator and noise covariance matrix served as inputs to *mne.minimum norm.apply inverse epochs* (with the argument “method” = dSPM and the estimated “lambda” = 0.0004) to obtain induced source estimates for all epochs and all participants. The argument “pick_ori” was set to “None” so that the source estimates were signed.

#### ROI source timeseries, and time-frequency (t-f) power

ROIs defined in fMRI data, which used SUMA’s std.141 surface mesh, were registered to the fsaverage surface. To co-register our source estimates defined on the participant’s anatomically constrained mesh and the fMRI masks defined on fsaverage, we used *mne.compute source morph*. Next, for each ROI, we extracted vertex-averaged induced timeseries by combining the timeseries of all of their sources using *mne.extract label time course* with argument mode set to “pca flip”. The same function/arguments were used to extract timeseries from the evoked source estimates. Time-frequency power spectra were obtained from each ROI timeseries using Morlet wavelets (*mne.time frequency.tfr array morlet*). We used frequencies 0.5 Hz and 1…100 Hz separated by 1 Hz intervals (via the argument “freqs”). We set the number of cycles of each wavelet (argument “n cycles”) to be a third of its frequency (e.g., for 30 Hz, 10 cycles were used) and arguments “use fft”=True and “output”=power. Finally, we *z*-scored the t-f power spectra per frequency for each epoch with respect to the baseline period (*t* = −0.5 s to *t* = 0 s). Note that these were calculated for both “induced” (one t-f power spectrum per ROI per epoch after first removing the ERP from each epoch) and “evoked” sources (a single evoked power time spectrum per ROI calculated on the ERP). Comparison of the power spectra during the baseline period revealed no differences between Novel and Repeat trials that would complicate subsequent interpretation of the results in any of the ROIs (Supplementary Fig. 19). To calculate average ROI induced power spectra for each participant, we averaged the power spectra over all epochs. In the end, this resulted in one induced and one evoked power spectrum for each ROI, per condition (Novel, Repeat), per participant. The process described above was the same for both overt and covert EEG data. It was also the same used to generate t-f power series of Repeat minus Novel differences projected for each vertex on the cortical surface as Supplementary Movies 1–4. Banded power estimates as a function of time for each condition, ROI, and participant were formed by further averaging the average ROI t-f power spectrum in each condition over the frequencies defining the bands (delta = 0.5–3 Hz, theta = 4–7 Hz, alpha = 8–12 Hz, beta = 13–29 Hz, gamma = 30–80 Hz).

### Region of interest (ROI) power analyses and correlations with fMRI RS and priming

Statistical comparisons across participants utilized paired *t*-tests for each ROI of Repeat-Novel power at each frequency and time sample. Comparisons per frequency band were then corrected for multiple comparisons using FDR (*q* < 0.05). Contiguous periods of “early” (Repeat > Novel) and “late” (Novel > Repeat) power differences in each frequency band that survived FDR correction were then defined as any period during the overall group effect lasting at least 50 ms for which the direction of the effect was the same. These periods were then averaged for each participant for subsequent correlations with fMRI repetition suppression and repetition priming effects (one “early” and one “late” average power difference for each participant and frequency band). Correlations (Spearman rank-order) were then carried out across participants between early and late power differences (Repeat-Novel) in each frequency band and ROI, with fMRI repetition suppression (Repeat-Novel beta coefficients) estimated for the same ROI and the magnitude of repetition priming (Cohen’s *d* values). Multiple comparisons across early/late periods, frequency bands, and ROIs were corrected using FDR (*q* < 0.05). Spearman rank-order correlations (and partial correlations for follow-up analyses) were utilized to guard against any potential effects of strong leverage (outlying) data points or possible violations of normality, and scatterplots correspondingly utilized ranks rather than raw values. Scatterplots of raw power differences, fMRI RS differences, and priming effect sizes for the left frontal region during the early period are presented in Supplementary Fig. S20. For replication purposes, covert naming power analyses utilized the time windows of significance from overt naming for the same ROIs (earliest time point to latest time point across frequencies), performing further FDR correction (*q* < 0.05) across time points within these windows.

### Evaluating within-participant EEG power correlates of priming magnitude

Correlations between EEG power differences and priming magnitude in the main analyses were conducted across participants, as with most prior studies. However, it is also possible to evaluate the agreement of power differences and priming magnitude within-participant by sub-selecting stimuli with either larger or smaller priming magnitudes. The 200 experimental stimuli were evenly divided into Strongly and Weakly primeable sub-sets (100 stimuli each) based on normative behavioral data from 60 participants in a prior study using the same stimuli^23^. These conditions were then further divided into Repeated and Novel conditions for a total of 4 conditions (Strongly Primeable Novel, Strongly Primeable Repeat, Weakly Primeable Novel, Weakly Primeable Repeat). Behavioral priming (Cohen’s *d*) was then calculated separately for Strongly and Weakly Primeable conditions for each participant to replicate the prior results for the current data (Strongly Primeable > Weakly Primeable). The EEG-induced power results were then recalculated separately for each participant and for each of the 4 conditions. Changes in EEG power due to repetition were evaluated for Strongly Primed and Weakly Primed stimuli separately, and the 2 (Strongly vs. Weakly Primed) × 2 (Novel, Repeat) interaction of power was assessed over a time window that covered the original “early” and “late” effects for each ROI (from 400 ms to 2000 ms post-stimulus). Multiple comparisons were corrected by the False Discovery Rate. Greater EEG power differences for Strongly Primeable stimuli would then constitute evidence of within-participant correlates of priming. For ROIs and frequency bands showing both across- and within-participant correlates of priming, we further evaluated across-participant correlations of the within-participant metrics (e.g., Strong-Weak Priming with Strong-Weak EEG Power Differences).

### Reporting summary

Further information on research design is available in the Nature Portfolio Reporting Summary linked to this article.

## Supplementary information

Transparent Peer Review file

Supplementary Information

Description of Additional Supplementary files

Supplementary Movie 1

Supplementary Movie 2

Supplementary Movie 3

Supplementary Movie 4

Supplementary Data 1

Reporting summary

## Data Availability

Data associated with this research are available through the Harvard Dataverse, https://dataverse.harvard.edu/dataverse/NamingEEGfMRI. Numeric data associated with bar plots and scatterplots in Figs. 1–5 are additionally provided in a single file as Supplementary Data 1.

## Code availability

Code associated with this research is available through the Harvard Dataverse, https://dataverse.harvard.edu/dataverse/NamingEEGfMRI.
