# Supplementary material for: Repetition-related reductions in neural activity support improved behavior through increases in oscillatory power
Source: Commun Biol. 2026 Jul 21;9:1321. doi: 10.1038/s42003-026-10668-0 (PMC13652815; doi:10.1038/s42003-026-10668-0)
Supplement: Supplementary file 1 [file 42003_2026_10668_MOESM1_ESM.pdf]

nature portfolio

Peer Review File

Repetition-related reductions in neural activity support improved behavior through increases in oscillatory power

Corresponding Author: Dr Adrian Gilmore

This file contains all reviewer reports in order by version, followed by all author rebuttals in order by version.

Version 0:

Reviewer comments:

Reviewer #1

(Remarks to the Author)
The authors recorded concurrent EEG-fMRI in two experiments to test different models of repetition suppression and the relation between repetition suppression and behavioral priming. Requiring overt (Expt 1) or covert (Expt 2) naming, correlations across subjects were observed for differences in gamma/theta/alpha power for repeat minus novel stimuli and priming magnitude, with a critical finding being an early increase and an earlier offset of oscillatory power for repeated vs. novel. While both experiments provide support for the Synchrony and Facilitation models, the covert naming experiment shows weaker effects and does not replicate findings related to fMRI RS and gamma power (perhaps due to the differences between covert vs. overt naming, along with differences in statistical power between Expt 2 and Expt 1, as the authors raise in the Discussion). Overall, the paper is well-written and its findings provide valuable insights into the nature of repetition suppression and the neural underpinnings of priming.

Comments:

1. Given that the ranks are plotted for the between-subjects correlations, it would be informative to see the distribution of the raw RS-related effect in EEG power (Repeat — Novel) in each band. While on average participants in the overt experiment show faster and larger responses to repeated stimuli in the gamma range, 3 subjects show little to no left frontal fMRI suppression (Fig. 1f). Depicting how many subjects show the RS-related effect in gamma power would inform interpretation of the across-subject correlations.

2. The paper separately tests for RS-related effects in the gamma, alpha, and theta bands in EEG. Did the authors have a priori hypotheses about whether the observed effects would be broadband or would be dominant in the gamma band? If so, it would be helpful to discuss this in the Introduction as this would further motivate the analyses. If not, it would be useful for the authors to guide the reader in the Discussion on how to interpret the findings across these different bands as well as why the gamma effects may have washed out in the covert experiment. The broader literature on associations between EEG gamma power and fMRI BOLD signals could additionally warrant discussion.

3. A major advantage of concurrent EEG-fMRI is the ability to link neural measures from the same timepoint for each modality by conducting trial-/item-level analyses. At present, the findings in the current manuscript rest on condition-level differences within subjects with relationships between variables then explored through between-subject associations. The manuscript would be further strengthened if the between-subject findings are corroborated by item-level analyses that link the observed EEG and fMRI signals. A challenge for conducting item-level analyses, given the design, is the lack of neuroimaging data during the initial object naming task, which is understandable given the time that would be required and the amount of effort and technological expertise already expended to collect the current data. Nonetheless, would it be possible to conduct potentially informative item-level analyses using the following approach: For the behavioral metric of item-level priming magnitude, the authors could compute the difference in RT between the repeated and initial presentation of each item (even if the experimental contexts are different). To relate priming magnitude to item-level RS effects, BOLD or gamma power etc. for each repeated item could be compared to the corresponding mean response to all novel items. Such an analysis would further bolster the concludions by potentially showing that early electrophysiological responses and BOLD RS associations additionally explain variance within subjects.

4. While the present findings are consistent with the Facilitation and Synchrony models, the reader may wonder whether the

data bear on the Sharpening model. On first pass, the data appear to be neutral with respect to and thus do not provide evidence for or against an additional role of sharpening in RS. Of course, the authors' inferences may differ from this read; brief treatment of the implications for Sharpening can be added to the Discussion.

Reviewer #2

(Remarks to the Author)
I co-reviewed this manuscript with one of the reviewers who provided the listed reports. This is part of the Communications Biology initiative to facilitate training in peer review and to provide appropriate recognition for Early Career Researchers who co-review manuscripts.

Reviewer #3

(Remarks to the Author)
The study of repetition processing with multimodal EEG/fMRI is interesting and the results presented are worth publishing to clarify open questions in the literature. The work is robust and the results presented are sound. However, there are some minor points that need correction as I pointed here:

Key technical question - the authors report a left/right distinction but I doubt it because it can be due to how the EEG setup is constructed. For the 256 channel setup how many amplifiers are connecting it? If more than one or with split references, and if they collect distinct channels, might happen that the left/right is due to some difference on the amplifiers recording them (imagine the channels of the right are for one and the left for the other amplifier) this can justify the left/right results. Please make it clear and solid in the methods.

fMRI-EEG: please revise the full manuscript to 'EEG/fMRI'.

Abstract:
Changes in oscillatory power and correlations need more objective measures. Please include statistical details in the abstract, such as effect sizes, to strengthen the interpretation.

Novelty and Context:
The study of repetition processing using multimodal approaches is interesting; however, it is unclear how this work goes beyond the current state of the art, as similar multimodal studies already exist. Please provide a clear statement of novelty and contextualize it (e.g., is the simultaneous acquisition the key innovation?).

Figures:
Figure 1, Panel G: Remove the trend line since no correlation is reported there.
Figure 2, Panel B: Add a colorbar label for clarity.
Figure 2 vs. Text (Line 160): There is an inconsistency—Figure 2C shows gamma-related results, but the text describes also alpha and other bands, which are not plotted. Please either add to the figure or revise the paragraph to match the figure content. Ensure consistency between figure and text. This paragraph about many frequency bands results that are not on the described figure.

Textual Issues:
Line 133 (Subtitle): The sentence is confusing; please revise for clarity.

Technical Detail:
Is the sampling rate (SR) of EEG acquisition sufficient to reduce MRI-related artifacts? Please clarify or justify.

Reviewer #4

(Remarks to the Author)
This fMRI-EEG study (n=40) by Gilmore and colleagues aims to explain why repeated exposure to stimuli leads to improved behavioural performance but reduced neural activity. They found that the peak of induced power was shifted earlier in time in response to repeat vs novel stimuli. The manuscript is clearly written, the methods are mostly sound, and the conclusions are mostly justified by the data. I have two major comments that I think the authors should address.

Spatial specificity: the pattern of results looks the same across ROIs. Given the poor spatial resolution afforded by EEG source reconstruction, the sources of the EEG effects may not be spatially specific. As a control, the authors could look at the brain regions that showed repetition enhancement in the fMRI data. Presumably the same effect should not be observed there.
Alternatively, the authors could repeat the power analyses but on the sensor-level data, and show the topography of the effect.

Frequency specificity: the pattern looks the same across frequencies. This could be explained by a change in the slope or

intercept of the 1/f background of the power spectrum. The authors could either show that 1/f parameters were not different between conditions, or perform the analyses on 1/f-corrected data.

Version 1:

Reviewer comments:

Reviewer #1

(Remarks to the Author)
The authors have adequately addressed the points raised during initial review. Critically, the additions of the within-participant effects of priming and raw scatterplots facilitate interpretation of strengths of their observed effects. The revisions with respect to support for different models and a priori hypotheses also strengthen the manuscript.

Reviewer #2

(Remarks to the Author)
I co-reviewed this manuscript with one of the reviewers who provided the listed reports. This is part of the Communications Biology initiative to facilitate training in peer review and to provide appropriate recognition for Early Career Researchers who co-review manuscripts.

Reviewer #3

(Remarks to the Author)
The authors edited the manuscript according to my concerns and I can recommend the publication at the current stage. Just a minor point, please remove the trend lines of the plots where these are not significant.

Reviewer #4

(Remarks to the Author)
I am somewhat satisfied with the authors' response to my first comment about spatial specificity, but I think they misunderstood my second comment on frequency specificity.

The condition effects reported in the study included a wide range of frequencies. Therefore this might be better explained as a difference in aperiodic properties of the spectrum. This has nothing to do with baseline differences.

In the case of both comments, I am not necessarily asking the authors to conduct additional analyses, but I think they should at the very least acknowledge the issues in the manuscript.

Open Access This Peer Review File is licensed under a Creative Commons Attribution 4.0 International License, which

permits use, sharing, adaptation, distribution and reproduction in any medium or format, as long as you give appropriate credit to the original author(s) and the source, provide a link to the Creative Commons license, and indicate if changes were made.
In cases where reviewers are anonymous, credit should be given to 'Anonymous Referee' and the source.
The images or other third party material in this Peer Review File are included in the article's Creative Commons license, unless indicated otherwise in a credit line to the material. If material is not included in the article's Creative Commons license and your intended use is not permitted by statutory regulation or exceeds the permitted use, you will need to obtain permission directly from the copyright holder.
To view a copy of this license, visit https://creativecommons.org/licenses/by/4.0/

Response to Reviewers' comments:

Reviewer #1 (Remarks to the Author):

The authors recorded concurrent EEG-fMRI in two experiments to test different models of repetition suppression and the relation between repetition suppression and behavioral priming. Requiring overt (Expt 1) or covert (Expt 2) naming, correlations across subjects were observed for differences in gamma/theta/alpha power for repeat minus novel stimuli and priming magnitude, with a critical finding being an early increase and an earlier offset of oscillatory power for repeated vs. novel. While both experiments provide support for the Synchrony and Facilitation models, the covert naming experiment shows weaker effects and does not replicate findings related to fMRI RS and gamma power (perhaps due to the differences between covert vs. overt naming, along with differences in statistical power between Expt 2 and Expt 1, as the authors raise in the Discussion). Overall, the paper is well-written and its findings provide valuable insights into the nature of repetition suppression and the neural underpinnings of priming.

Comments:

1. Given that the ranks are plotted for the between-subjects correlations, it would be informative to see the distribution of the raw RS-related effect in EEG power (Repeat — Novel) in each band. While on average participants in the overt experiment show faster and larger responses to repeated stimuli in the gamma range, 3 subjects show little to no left frontal fMRI suppression (Fig. 1f). Depicting how many subjects show the RS-related effect in gamma power would inform interpretation of the across-subject correlations.

We appreciate the Reviewer's question about the distributions of the raw variables. It is not necessarily the case that each individual subject shows significant effects of each variable of interest (priming, differences in EEG power, fMRI RS) by trial. This would likely have required increasing the number of trials, perhaps substantially. One can therefore not make many strong conclusions based on the individual subject's values on these measures. However, we certainly understand the reviewer's overall concern about only plotting ranks, and we agree that showing raw values can be helpful. Given the large number of plots and results, we have focused on the significant relationships for the left frontal ROI, which is the only region to show significant relationships among priming, power, and fMRI RS (for the gamma frequency band). Scatterplots are shown below for the "early" period (during which power is greater for Repeat relative to the Novel condition), with zero lines plotted for both the x- and y-axes. The distributions of power differences are somewhat skewed across subjects, which is why we chose to plot ranks and use the non-parametric version of correlation (Spearman's *r*) in the analyses. For the left frontal ROI, significant correlations were observed in the gamma, beta, and alpha bands. For all three bands, most subjects have positive values (34/40 for gamma, 34/40 for beta, and 32/40 for alpha), no individual subject has significant results in the opposite direction (although this is underpowered in the individual subjects), and group medians are strongly different from zero for all 3 bands (Sign tests: gamma $p = 8.4\times10^{-6}$, beta $p = 8.4\times10^{-6}$, alpha $p = 1.8\times10^{-4}$). We now include this information in the supplementary materials of the revised manuscript (Supplemental Figure 20).

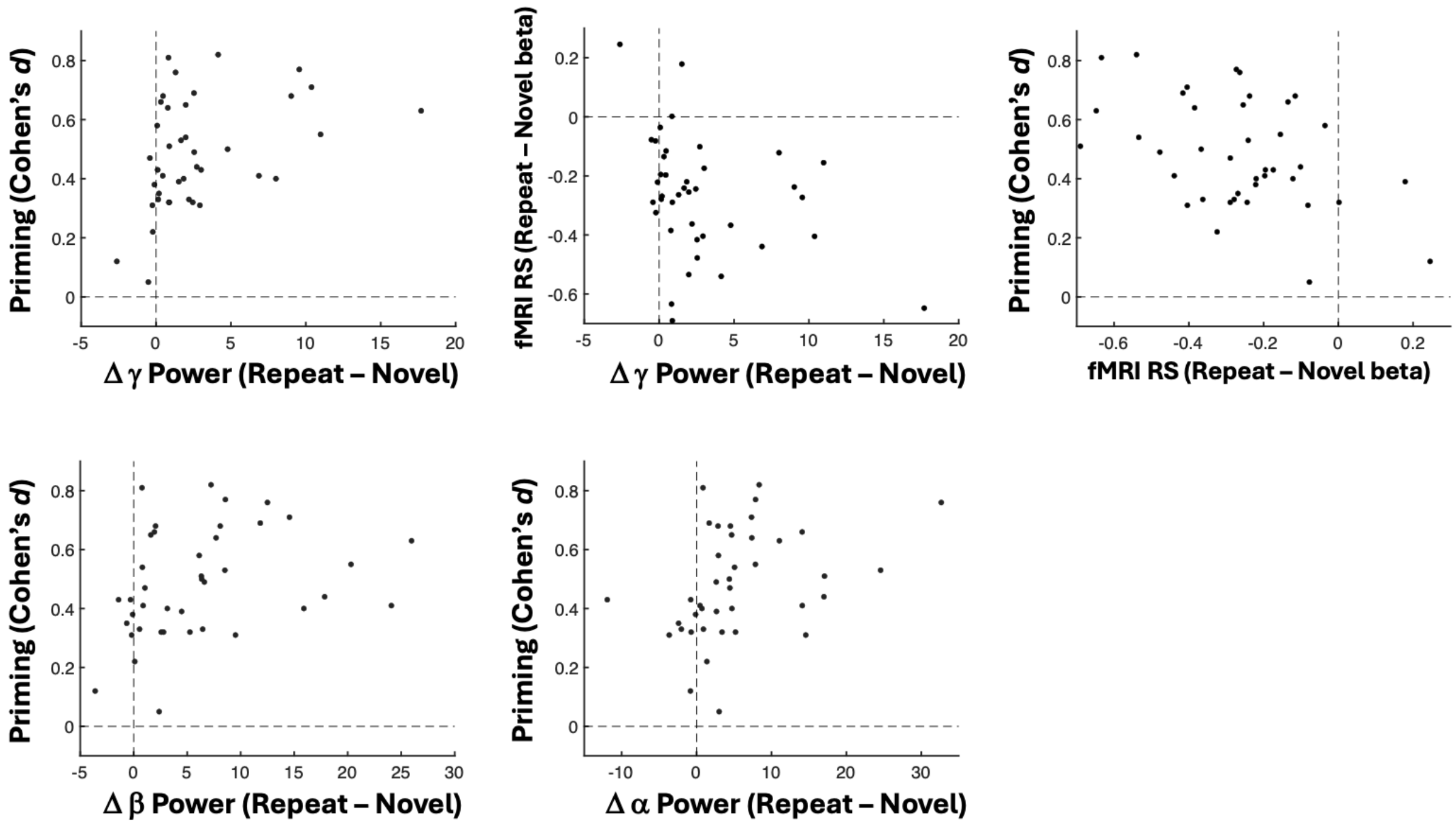

Supplemental Figure S20. Scatterplots of raw power differences, fMRI RS differences, and priming effect sizes for the left frontal region during the early period. Results are shown for all frequency bands and variables of interest with significant correlations (Spearman) during this period.

2. The paper separately tests for RS-related effects in the gamma, alpha, and theta bands in EEG. Did the authors have a priori hypotheses about whether the observed effects would be broadband or would be dominant in the gamma band? If so, it would be helpful to discuss this in the Introduction as this would further motivate the analyses. If not, it would be useful for the authors to guide the reader in the Discussion on how to interpret the findings across these different bands as well as why the gamma effects may have washed out in the covert experiment. The broader literature on associations between EEG gamma power and fMRI BOLD signals could additionally warrant discussion.

There are indeed good reasons for examining these three bands based on prior literature. Previous experiments of the effect of stimulus repetition on MEG power during object identification in humans have found effects in theta, alpha and low beta (ranging from 5 to 15 Hz; Ghuman et al., 2008, PNAS; Gilbert et al., 2010, Front Human Neurosci). Studies in animals (cats, monkeys) have found effects of stimulus repetition in alpha (von Stein et al.,2000, PNAS) and gamma frequencies (Brunet et al., 2014, PNAS; Hansen & Dragoi, 2011, PNAS; Peter et al., 2021, Cell Reports; Wang et al., 2011, J Neurosci). Previous EEG studies in humans have also found effects in gamma (e.g. Gruber & Müller, 2002, Cogn Brain Res; Gruber, 2005, Cereb Cortex; Busch et al., 2006, NeuroImage). Whereas we referenced these literatures in our discussion, we now also bring them up in the Introduction, as suggested by the Reviewer. This new text is copied below.

Based on prior literature, electrophysiological effects might be expected to emerge in the gamma band or in the theta/alpha bands. Changes in induced power in the gamma range have previously been associated with stimulus repetition in both microelectrode recordings in animals[24–27] and EEG in humans[28–30]. On the other hand, MEG data in humans have found changes in evoked power and phase locking in frontal and fusiform regions in theta, alpha and low beta frequencies[21,31]. Although the current experiment will consider effects ranging from delta to gamma, there is a priori evidence to suggest that these frequency bands, in particular, may speak to the relation between repetition suppression and repetition priming.

3. A major advantage of concurrent EEG-fMRI is the ability to link neural measures from the same timepoint for each modality by conducting trial-/item-level analyses. At present, the findings in the current manuscript rest on condition-level differences within subjects with relationships between variables then explored through between-subject associations. The manuscript would be further strengthened if the between-subject findings are corroborated by item-level analyses that link the observed EEG and fMRI signals. A challenge for conducting item-level analyses, given the design, is the lack of neuroimaging data during the initial object naming task, which is understandable given the time that would be required and the amount of effort and technological expertise already expended to collect the current data. Nonetheless, would it be possible to conduct potentially informative item-level analyses using the following approach: For the behavioral metric of item-level priming magnitude, the authors could compute the difference in RT between the repeated and initial presentation of each item (even if the experimental contexts are different). To relate priming magnitude to item-level RS effects, BOLD or gamma power etc. for each repeated item could be compared to the corresponding mean response to all novel items. Such an analysis would further bolster the conclusions by potentially showing that early electrophysiological responses and BOLD RS associations additionally explain variance within subjects.

We thank the Reviewer for their excellent suggestion to examine within-subject effects more deeply, as this has enabled us to further strengthen the overall case we have argued in the earlier version of the manuscript.

This was also a consideration a subset of us tackled in an earlier, conceptually-related paper (Gotts et al., 2021). In those earlier analyses, it became apparent that the test-retest reliability of RTs is fairly low (~.15), and so we developed an approach in which we normed each stimulus for its average amount of priming. We then divided our stimuli into 2 groups—those in which priming is relatively strong (hereafter, "Strongly" primeable), and those in which it was relatively weaker ("Weakly" primeable). We were able to use these previously derived norms on our current data, and in so doing we were able to divide the stimuli in our current EEG/fMRI experiment into equally sized Strongly and Weakly primeable set of stimuli (100 of each type, divided further into Repeat and Novel conditions within each).

Comparisons of the stimulus categories within our current participants replicated the prior separation: Strongly primeable items exhibit larger magnitudes of priming (quantified using Cohen's *d*) than did Weakly primeable items, yet both were associated with significant priming effects in general (panel A of inset figure, below). With the trials thus split, we again examined

induced EEG power effects for Novel and Repeat stimuli, separately for Strongly and Weakly primeable items in each of our ROIs. In many, Strongly primeable had qualitatively greater differences than did Weakly primeable, and this was confirmed by the interaction tests comparing Novel/Repeat and Strongly/Weakly primeable status (see Supplemental Figures S11-S18).

We also looked for correlations between the Strongly and Weakly primeable items using the same across-participant approach applied previously. The left fusiform ROI stood out here because we observed significant correlations within it between priming magnitude and induced gamma power for Strongly primeable items, but more importantly because we also identified a correlation between the difference in priming between Strongly and Weakly primeable items, and between Novel and Repeat items in the Strongly and Weakly primeable conditions. This was the only ROI to show this complete combination of associations. Others showed some, but not all, of these differences. As such, we have highlighted the left fusiform as a potentially critical region in our analysis, and include a figure (inset below, along with its caption) in which our analyses of it are summarized. Similar figures for other ROIs/frequency bands are now included as supplementary materials. Although we typically include a full copy of the modified text for our revisions in response letter, we refer the Reviewer to pages 18-20, and the top of p. 23, for the relevant updates due to their overall length.

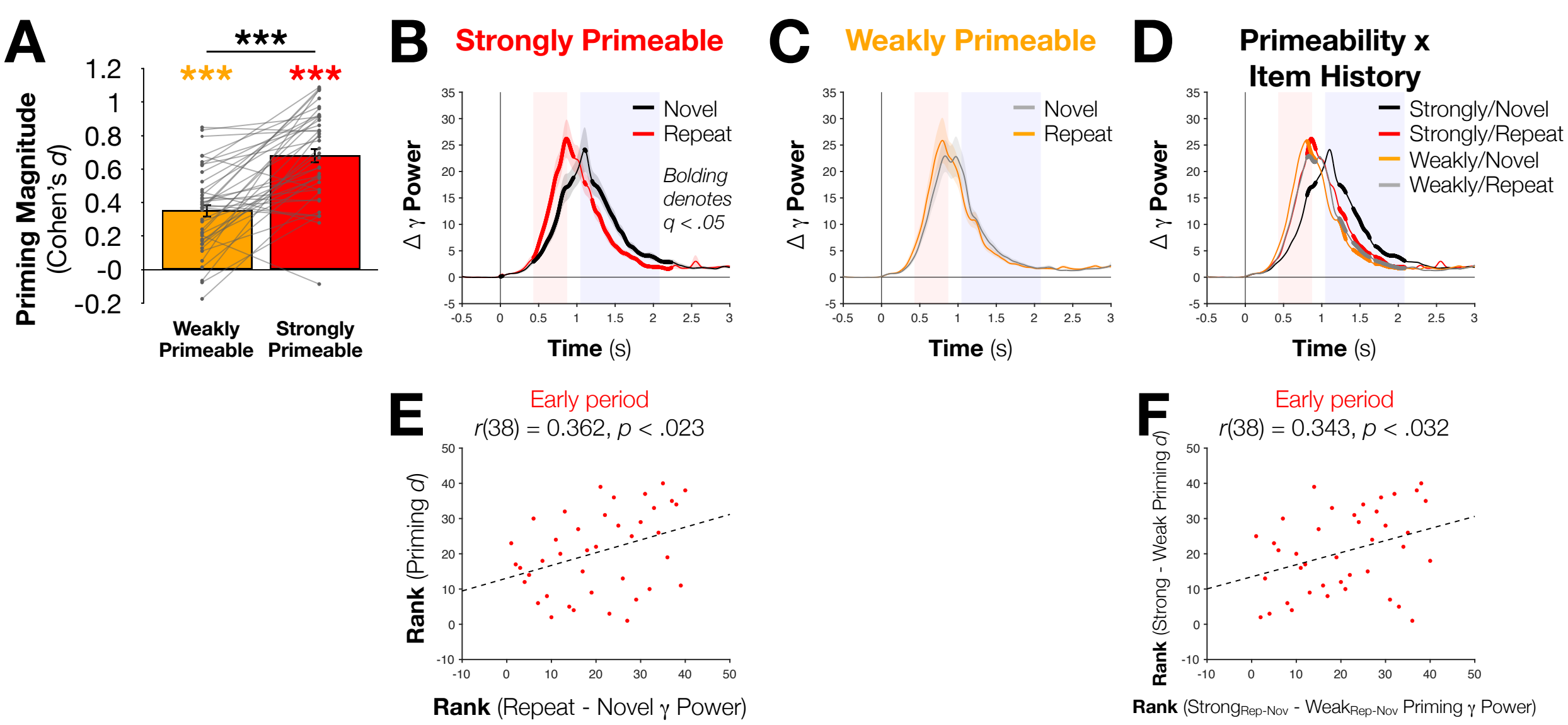

Fig. 5. Results of within-participant comparisons of priming magnitude and their association with induced gamma-band EEG power differences in the left fusiform cortex. A) Stimuli were separated into Strongly and Weakly primeable groups (100 items each) based on average primeability in an earlier study[23]. Although both levels of primeability were associated with significant repetition priming, Strongly primeable items were associated with significant greater effect sizes. B) Strongly primeable items were associated with induced gamma EEG power differences, but C) Weakly primeable items did not show significant differences. Red and blue background colors reflect periods of significant Novel and Repeat power differences corresponding to those in Figure 2C. The interaction between Novel and Repeat item history for Strongly and Weakly primeable items was quantified, with significant effects emerging in early and

late windows. E) Subsequent analyses revealed that, across participants, a significant correlation was observed between priming magnitude and induced gamma power differences for Strongly primeable items. The behavioral difference in priming magnitude between Strongly and Weakly primeable items also correlated, across participants, with the difference between Novel and Repeat items in the Strongly and Weakly primeable conditions. *** depicts p < .001. Colored asterisks reflect the results of one-sample t-tests whereas the black asterisks reflect a paired samples t-test. All t-tests were two tailed.

4. While the present findings are consistent with the Facilitation and Synchrony models, the reader may wonder whether the data bear on the Sharpening model. On first pass, the data appear to be neutral with respect to and thus do not provide evidence for or against an additional role of sharpening in RS. Of course, the authors' inferences may differ from this read; brief treatment of the implications for Sharpening can be added to the Discussion.

We agree with the Reviewer that the present data do not provide evidence for or against Sharpening, although some members of our research team have tested the Sharpening account twice with fMRI using related paradigms (Gotts et al., 2015, Neuropsychologia; Gotts et al., 2021, Comms Biol). In our view, the current data, and especially the EEG component, do not provide the necessary information about firing rate distributions and how these may differ across novel and repeat items. We now discuss our findings with respect to the Sharpening and Predictive Coding models in our discussion, and we reproduce the new text below.

Thus far, our discussion has emphasized the support of the current results for the Synchrony and Facilitation models. This should not, however, be taken to imply that the EEG/fMRI data presented here should be viewed as evidence against the Sharpening or Predictive Coding models. Rather, the current data, and especially the EEG component, cannot provide information about firing rate distribution differences between novel and repeat items that we view as critical for a direct test of the Sharpening account. On the other hand, tests of predictive coding require estimation of directional connectivity, and volumetric spread of the EEG signal renders strong tests of this model difficult in the current dataset. Future work, focusing directly on Sharpening or Predictive Coding, would complement that presented here.

Reviewer #2 (Remarks to the Author):

I co-reviewed this manuscript with one of the reviewers who provided the listed reports. This is part of the Communications Biology initiative to facilitate training in peer review and to provide appropriate recognition for Early Career Researchers who co-review manuscripts.

We appreciate R2's contributions to the review process and hope they found their experience useful from a training perspective.

Reviewer #3 (Remarks to the Author):

The study of repetition processing with multimodal EEG/fMRI is interesting and the results

presented are worth publishing to clarify open questions in the literature. The work is robust and the results presented are sound. However, there are some minor points that need correction as I pointed here:

Key technical question - the authors report a left/right distinction but I doubt it because it can be due to how the EEG setup is constructed. For the 256 channel setup how many amplifiers are connecting it? If more than one or with split references, and if they collect distinct channels, might happen that the left/right is due to some difference on the amplifiers recording them (imagine the channels of the right are for one and the left for the other amplifier) this can justify the left/right results. Please make it clear and solid in the methods.

The lateralization of regions of interest was motivated by prior work examining repetition suppression and repetition priming that exclusively used fMRI (Gotts et al., 2021) and was therefore replicated in our current fMRI data (Fig 1). EEG was not, therefore, the original basis for any left/right distinctions made in this manuscript. However, we agree that the use of multiple amplifiers or split references could potentially have been problematic. In our experiments, all electrode channels were connected to a single Net Amps 400 amplifier. Furthermore, as we reported in our Methods, all were recorded using a Cz reference. We have modified our methods to now read as follows to make it clearer that we used a single amplifier for all channels (changes bolded):

> *"EEG signals **from all channels** were digitized at a 1000 Hz sampling rate with a **single** Net Amps 400 amplifier and recorded onto a MacBook Pro laptop (Apple Inc., Cupertino, CA) running EGI NetStation."*

fMRI-EEG: please revise the full manuscript to 'EEG/fMRI'.

We have revised our terminology as requested.

Abstract:
Changes in oscillatory power and correlations need more objective measures. Please include statistical details in the abstract, such as effect sizes, to strengthen the interpretation.

As suggested, we now offer more information, as well as effect sizes, in the abstract.

Novelty and Context:
The study of repetition processing using multimodal approaches is interesting; however, it is unclear how this work goes beyond the current state of the art, as similar multimodal studies already exist. Please provide a clear statement of novelty and contextualize it (e.g., is the simultaneous acquisition the key innovation?).

Concisely, we believe that the primary novel contribution—a better understanding of the mechanisms that link repetition suppression to priming—comes from the combination of tools and approaches we have made use of in this study. Studies using electrophysiological methods and fMRI have proceeded largely in parallel, even when these are being employed by the same researchers in the same lab group (i.e., Race et al., 2009, JoCN employed fMRI but not EEG,

whereas Race et al., 2010, Cereb Cortex utilized EEG but not fMRI). This does not imply that EEG and fMRI findings have not been simultaneously considered in reviews (indeed, some of us have done this ourselves in earlier work, e.g., Gotts et al., 2012, Cognit Neurosci), but rather we mention this to highlight the opportunity provided by a multimodal investigation that uses both approaches simultaneously. As we discuss in our introduction, each approach has its limitations, but making headway on the question of RS and priming appears, by now, to require solutions that enable a high degree of functional-anatomic precision and rapid temporal sampling.

Furthermore, our use of spoken responses considerably increases the effect size of priming, which combines with our large N = 40 sample to provide significantly more power than is typically available to study RS and priming. Although the application of facial electrode regression was not novel in this experiment, we feel that the current use case provides a concrete example of how flexible this approach is. Indeed, we continue to observe frontal effects despite their proximity to the electrodes being used for nuisance regression.

Following a comment from Reviewer #1, we have added a novel analysis that looks at the strength of within-subject priming effects, and this makes even better use of the properties of our dataset. We now demonstrate that the same basic pattern is observable within participants that we previously reported at the across-participant level.

On the novelty point, we would also like to highlight the findings in the left frontal region. In the gamma frequency band, we observe correlations among all three of our primary measures (behavioral priming, fMRI RS, and differences in EEG power). This allowed us to perform a partial correlation analysis that showed that fMRI RS does not account for unique variance in priming, but EEG power does. This novel finding would not have been possible without simultaneous recordings of fMRI and EEG signals.

Figures:
Figure 1, Panel G: Remove the trend line since no correlation is reported there.

We appreciate the Reviewer's suggestion to ensure clarity. However, after testing its removal, we felt that it was visually clearer to have the bar remain so that the lack of fitness along the line can be better appreciated. As before, we also include the correlation magnitude and corresponding *p*-value above the plot so that all information is clear to readers.

Figure 2, Panel B: Add a colorbar label for clarity.

Thank you for catching this omission. We have corrected both Fig 2B and also Fig 4B to now include color bar labels.

Figure 2 vs. Text (Line 160): There is an inconsistency—Figure 2C shows gamma-related results, but the text describes also alpha and other bands, which are not plotted. Please either add to the figure or revise the paragraph to match the figure content. Ensure consistency between figure and text. This paragraph about many frequency bands results that are not on the described figure.

We apologize for presenting data in a confusing manner. We now reference Supplementary Table 1 and Supplementary Figures 1-2 directly after mentioning the time windows, as this is where all the information is summarized. We now also clearly state that Fig. 2C represents a visualization of gamma band effects, specifically. The modified section of text is reproduced below (changes bolded):

> *These followed a stereotypical pattern, with an early increase in induced power for the Repeat relative to the Novel condition around 300-500 ms post-stimulus (earliest onsets detected in the gamma frequency range at 280 ms, 431 ms, 519 ms, and 385 ms in the left frontal, left fusiform, dACC/pre-SMA and right fusiform regions, respectively) through approximately 800-900 ms post-stimulus (latest offsets in the alpha frequency range at 888 ms, 870 ms, 892 ms, and 917 ms in the left frontal, left fusiform, dACC/pre-SMA and right fusiform regions, respectively;* ***Supplementary Figs. 1-2; Supplementary Table 1****; see bolded segments backed by light red background in Fig. 2C* ***for visualization effects in the gamma band, specifically****). This was followed by a more rapid decrease in induced power for the Repeat condition relative to the Novel condition around 1100-1200 ms post-stimulus (earliest onsets detected in the beta frequency range at 1171 ms, 1150 ms, 1081 ms, and 1112 ms in the left frontal, left fusiform, dACC/pre-SMA and right fusiform regions, respectively) through approximately 2100-2300 ms post-stimulus (latest offsets in the theta frequency range at 1888 ms, 2381 ms, 2138 ms, and 2609 ms in the left frontal, left fusiform, dACC/pre-SMA and right fusiform regions, respectively;* ***Supplementary Figs. 1-2; Supplementary Table 1;*** *see bolded segments backed by light blue background in Fig. 2C* ***for visualization effects in the gamma band, specifically****).*

Textual Issues:
Line 133 (Subtitle): The sentence is confusing; please revise for clarity.

We thank the Reviewer for pointing out that our language was difficult to parse. We have modified the section title to now read as follows:

> ***EEG oscillatory power rises and falls earlier with stimulus repetition and does so in a manner correlated with the magnitude of behavioral priming***

Technical Detail:
Is the sampling rate (SR) of EEG acquisition sufficient to reduce MRI-related artifacts? Please clarify or justify.

This is an excellent question relating to a critical part of our EEG preprocessing. The MR artifact has a frequency of about 9.8 Hz (during EPI scanning, slices are acquired every 0.102 seconds). At our sampling rate of 1000 Hz, this is well below our Nyquist limit of 500 Hz. However, our approach to removing MR-related artifact was an artifact subtraction that used a windowed averaging, which comes as a standard processing step in our NetStation software. This approach would allow us to remove MR artifacts even with temporal aliasing. We have updated our Methods text to clarify the removal of MR artifact from our EEG data. Updated text is as follows (changes bolded):

*EEG data were initially processed **to remove MR-induced gradient artifacts and Ballistocardiogram (BCG) artifacts using standard NetStation functions**. First, MR-induced gradient artifacts were removed from the EEG using an average artifact subtraction based on a sliding window of 10 TR pulse events. **BCG** artifacts were then removed **in a subsequent step**. For the participants for whom EKG sensors were not available…"*

Reviewer #4 (Remarks to the Author):

This fMRI-EEG study (n=40) by Gilmore and colleagues aims to explain why repeated exposure to stimuli leads to improved behavioural performance but reduced neural activity. They found that the peak of induced power was shifted earlier in time in response to repeat vs novel stimuli. The manuscript is clearly written, the methods are mostly sound, and the conclusions are mostly justified by the data. I have two major comments that I think the authors should address.

Spatial specificity: the pattern of results looks the same across ROIs. Given the poor spatial resolution afforded by EEG source reconstruction, the sources of the EEG effects may not be spatially specific. As a control, the authors could look at the brain regions that showed repetition enhancement in the fMRI data. Presumably the same effect should not be observed there. Alternatively, the authors could repeat the power analyses but on the sensor-level data, and show the topography of the effect.

This is an excellent point and we agree that it is important to rule out that we are simply capturing a "global" effect. We first considered a comparison of RE to RS regions, as suggested by the Reviewer. However, given the smoothness of the EEG data, the relatively small size of RE regions as compared to RS regions, and the proximity of RE to RS effects, the results of this approach are necessarily difficult to interpret.

Instead, we have chosen to use a source estimation approach to visualize, on the cortical surface, the topography of repetition-related differences. We think that this issue makes more sense to evaluate in source space, as sensor space (in theory) will tend to reflect the spatial blurring of the underlying sources. We averaged the Repeat > Novel effects across the time steps that range from the earlier to latest periods of FDR-corrected significance across our 4 ROIs, and thresholded the map using the same values as in the supplementary movies we included in our initial submission. As with the movies, we also include outlines of our 4 ROIs. We observed appreciable differences in the strength of Repeat-Novel differences across the cortex; a result inconsistent with the "global" hypothesis. The strongest effects emerge in the left temporal lobe (overlapping with the left fusiform ROI) and in the insula bilaterally, with more limited effects observed overlapping with the left frontal and right fusiform ROIs. Due to the spatial spreading of the signals in EEG, though, significant power differences are observed over most surface nodes.

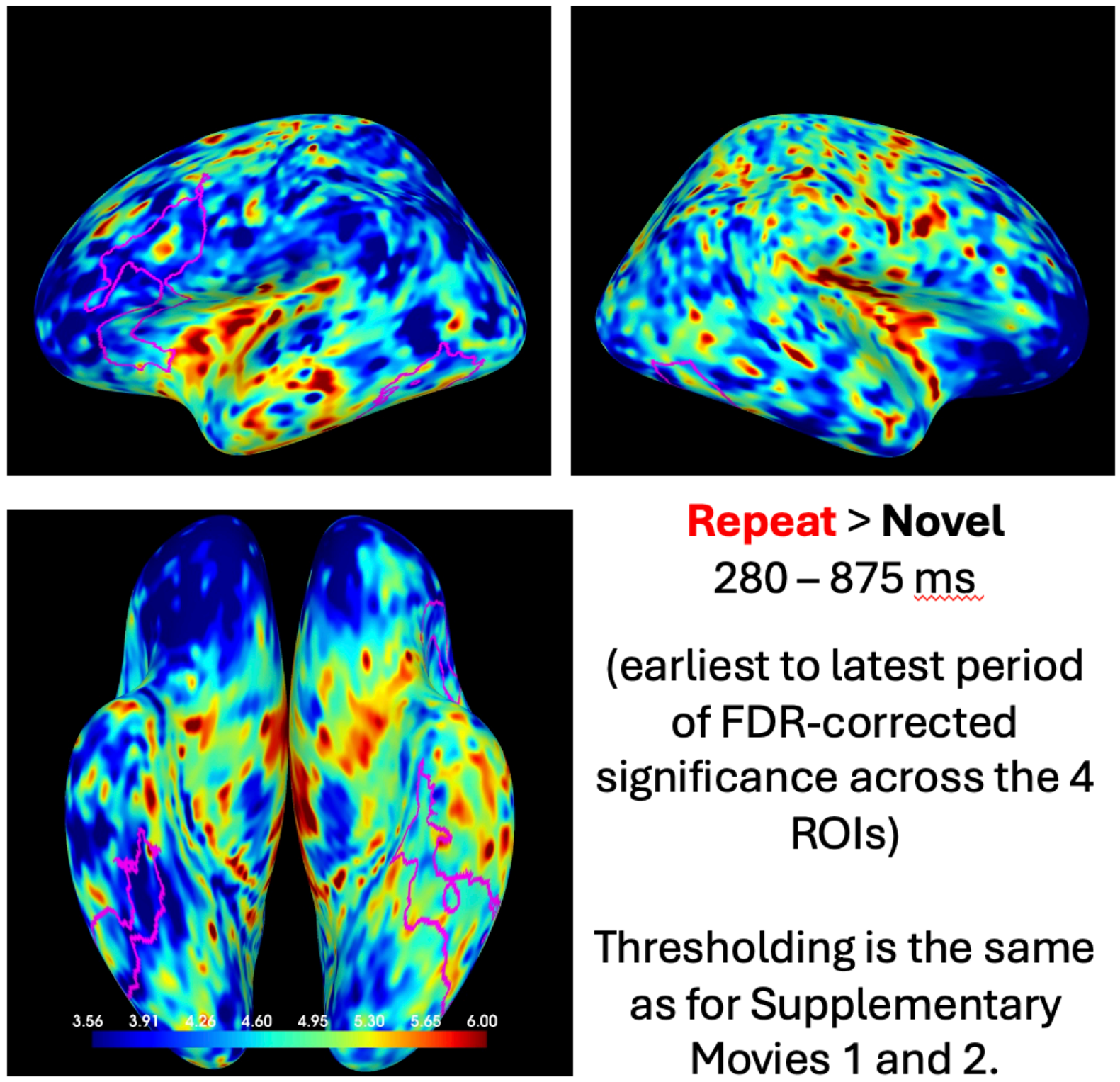

As this analysis recapitulates information that is already available in the included supplementary movies (and, indeed, is more limited, because those can be set to different times), we have not made additional modifications to the manuscript regarding this point. We nonetheless thank the reviewer for encouraging us to pause and reassess the specificity of our spatial effects.

We would also like to draw attention to the previous analyses of the sensor-level contributions to the sources (Supplementary Figure 5, which provides a map of sorts of which sensors contribute most strongly to each source-space ROI). These maps give some sense as to the spatial resolution of the EEG data at the sensor-level.

Frequency specificity: the pattern looks the same across frequencies. This could be explained by a change in the slope or intercept of the 1/f background of the power spectrum. The authors could either show that 1/f parameters were not different between conditions, or perform the analyses on 1/f-corrected data.

The reviewer is correct to point out that differences in the power spectrum during baseline periods could produce spurious differences in our comparisons between Novel and Repeat item power that could result in broadband differences. To address this, we computed the average power spectrum during the baseline period (-0.5 s to 0 s) for each trial, for each condition, in each ROI. Differences here would have impacted trial-wise normalization and could have distorted our findings. However, as can be appreciated, there is no significant difference in any frequency in any ROI. The lack of differences during this time also follows from our counterbalancing approach, which ensured that there was no means of predicting the condition in

trial N+1 based on the condition of trial N. Given the importance of this analysis to our findings, we also include the plot below as Supplementary Figure 19.

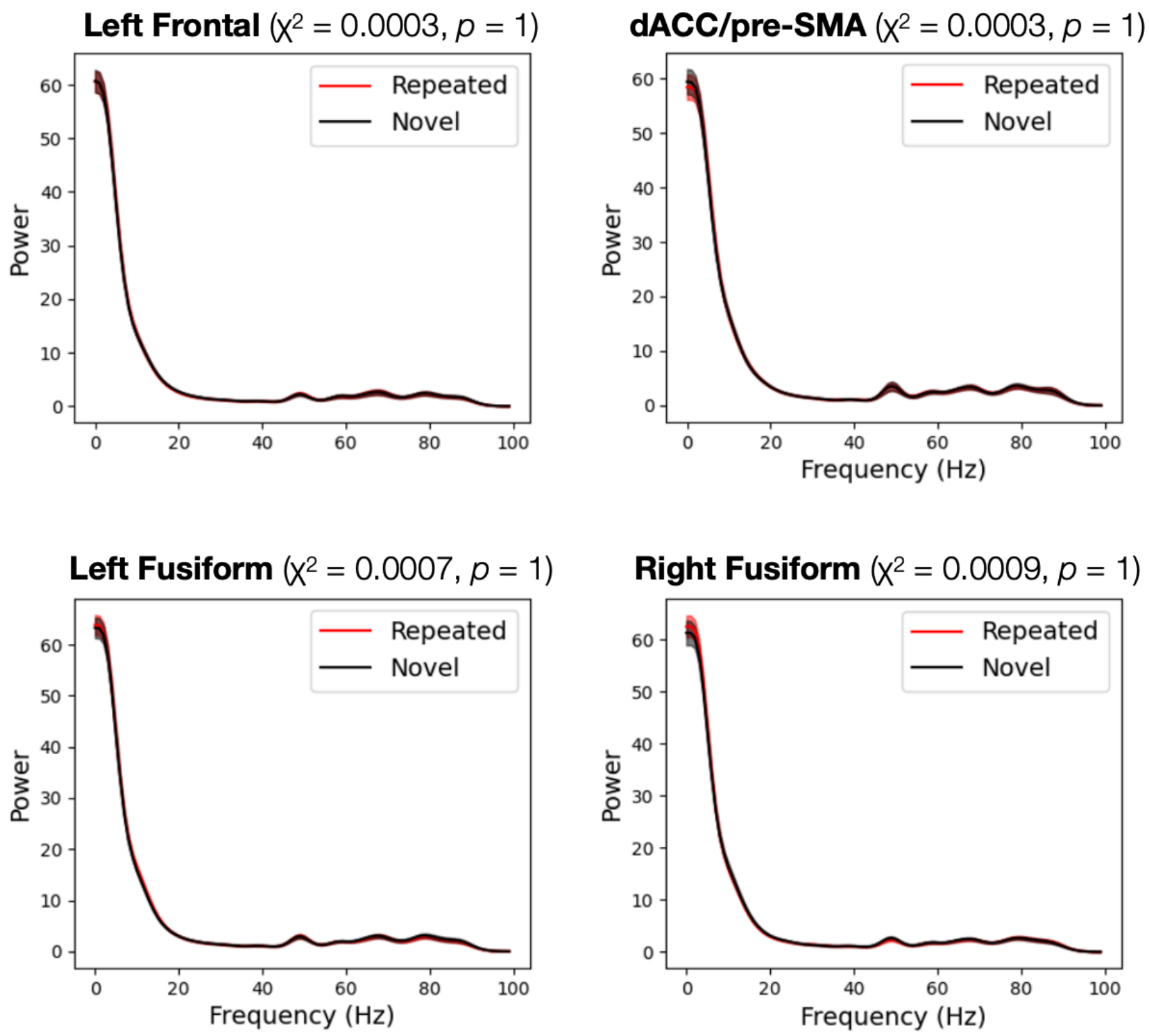

Supplementary Figure 19. Comparisons of baseline period power spectra for Novel (black) and Repeat (red) items identified no differences in any ROI. No differences in power were observed in any frequency in any ROI.

RESPONSE TO REVIEWERS' COMMENTS:

Reviewer #1 (Remarks to the Author):

The authors have adequately addressed the points raised during initial review. Critically, the additions of the within-participant effects of priming and raw scatterplots facilitate interpretation of strengths of their observed effects. The revisions with respect to support for different models and a priori hypotheses also strengthen the manuscript.

We thank the Reviewer for their clear assessment of our modifications, as well as their thoughtful engagement with our initial submission.

Reviewer #2 (Remarks to the Author):

I co-reviewed this manuscript with one of the reviewers who provided the listed reports. This is part of the Communications Biology initiative to facilitate training in peer review and to provide appropriate recognition for Early Career Researchers who co-review manuscripts.

We again thank this Reviewer for assisting with the review process.

Reviewer #3 (Remarks to the Author):

The authors edited the manuscript according to my concerns and I can recommend the publication at the current stage. Just a minor point, please remove the trend lines of the plots where these are not significant.

We have removed non-significant correlation trend lines, as requested. This has impacted Fig. 1G and the two middle plots in Fig. 4E. Trend lines are now only retained in cases where the correlations were significant at $p < .05$ or, in one case, where it approached significance ($p = .057$, Fig. 4B, left) in the Covert dataset in a way that replicated a significant effect in the Overt dataset.

Reviewer #4 (Remarks to the Author):

I am somewhat satisfied with the authors' response to my first comment about spatial specificity, but I think they misunderstood my second comment on frequency specificity.

The condition effects reported in the study included a wide range of frequencies. Therefore this might be better explained as a difference in aperiodic properties of the spectrum. This has nothing to do with baseline differences.

In the case of both comments, I am not necessarily asking the authors to conduct additional analyses, but I think they should at the very least acknowledge the issues in the manuscript.

We apologize for misunderstanding one of the Reviewer’s concerns regarding our prior submission. As they suggest, we now include the following verbiage after discussing limitations

of our work with respect to the Sharpening and Predictive Coding models on p. 17. New text is bolded at the bottom of the paragraph:

> *Thus far, our discussion has emphasized the support of the current results for the Synchrony and Facilitation models. This should not, however, be taken to imply that the EEG/fMRI data presented here should be viewed as evidence against the Sharpening or Predictive Coding models. Rather, the current data, and especially the EEG component, cannot provide information about firing rate distribution differences between novel and repeat items that we view as critical for a direct test of the Sharpening account. On the other hand, tests of predictive coding require estimation of directional connectivity, and volumetric spread of the EEG signal renders strong tests of this model difficult in the current dataset. Future work, focusing directly on Sharpening or Predictive Coding, would complement that presented here.* ***In addition, although the power differences reported here are consistent with predictions of the Synchrony model, differences in aperiodic properties of the power spectrum across task conditions could, in principle, also contribute to such observations. Future work that is optimized for assessing cross-regional coupling while separately modeling oscillatory and aperiodic activity[38-40] would directly address this possibility.***

Newly added references:

38. Wen, H. & Liu, Z. Separating fractal and oscillatory components in the power spectrum of neurophysiological signal. *Brain Topogr* **29**, 13–26 (2016).

39. Donoghue, T. *et al.* Parameterizing neural power spectra into periodic and aperiodic components. *Nat Neurosci* **23**, 1655–1665 (2020).

40. Gerster, M. *et al.* Separating Neural Oscillations from Aperiodic 1/f Activity: Challenges and Recommendations. *Neuroinform* **20**, 991–1012 (2022).
