# Supplementary material for: Repetition-related reductions in neural activity support improved behavior through increases in oscillatory power
Source: Commun Biol. 2026 Jul 21;9:1321. doi: 10.1038/s42003-026-10668-0 (PMC13652815; doi:10.1038/s42003-026-10668-0)
Supplement: Supplementary file 2 [file 42003_2026_10668_MOESM2_ESM.pdf]

Supplementary figures and tables for:

Repetition-related reductions in neural activity support improved behavior through increases in oscillatory power

Authors:
Adrian W. Gilmore, Leonardo Claudino, Cassandra M. Levesque, Anna M. Agron, Peter J. Molfese, Vinai Roopchansingh, Michael D. Rugg, Stephen J. Gotts, Alex Martin

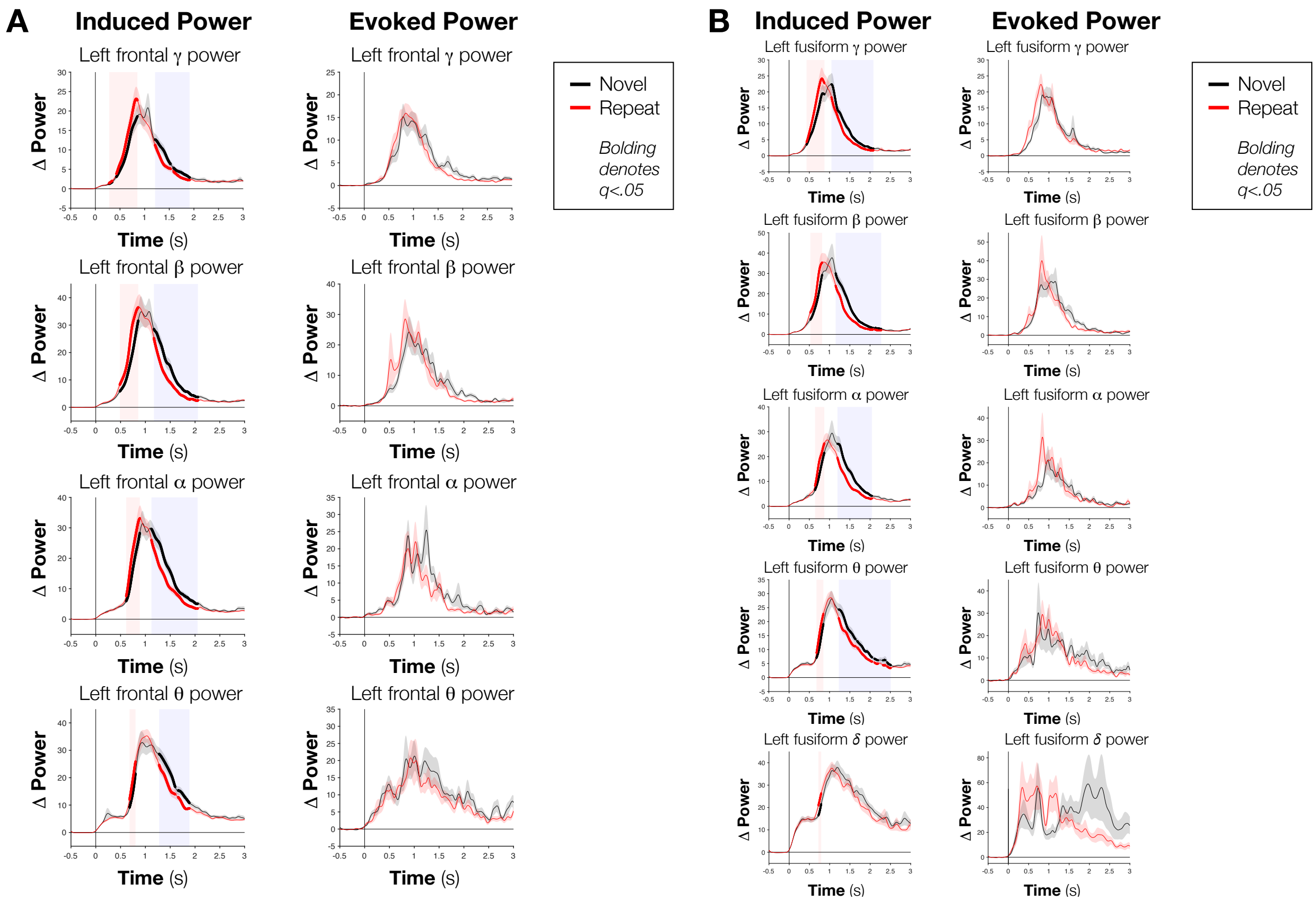

Supplementary Fig. 1. Effects of repeated object identification on induced versus evoked power for the fMRI-defined A) left frontal and B) left fusiform regions. Evoked power corresponds to power estimates after first averaging across trials in the time domain per condition, whereas induced power is calculated per trial after first subtracting the trial-averaged evoked response and then averaging across trials. FDR-corrected periods are shown with bolded lines, with red lines representing change in power relative to the baseline period for the Repeat condition and black lines representing the Novel condition.

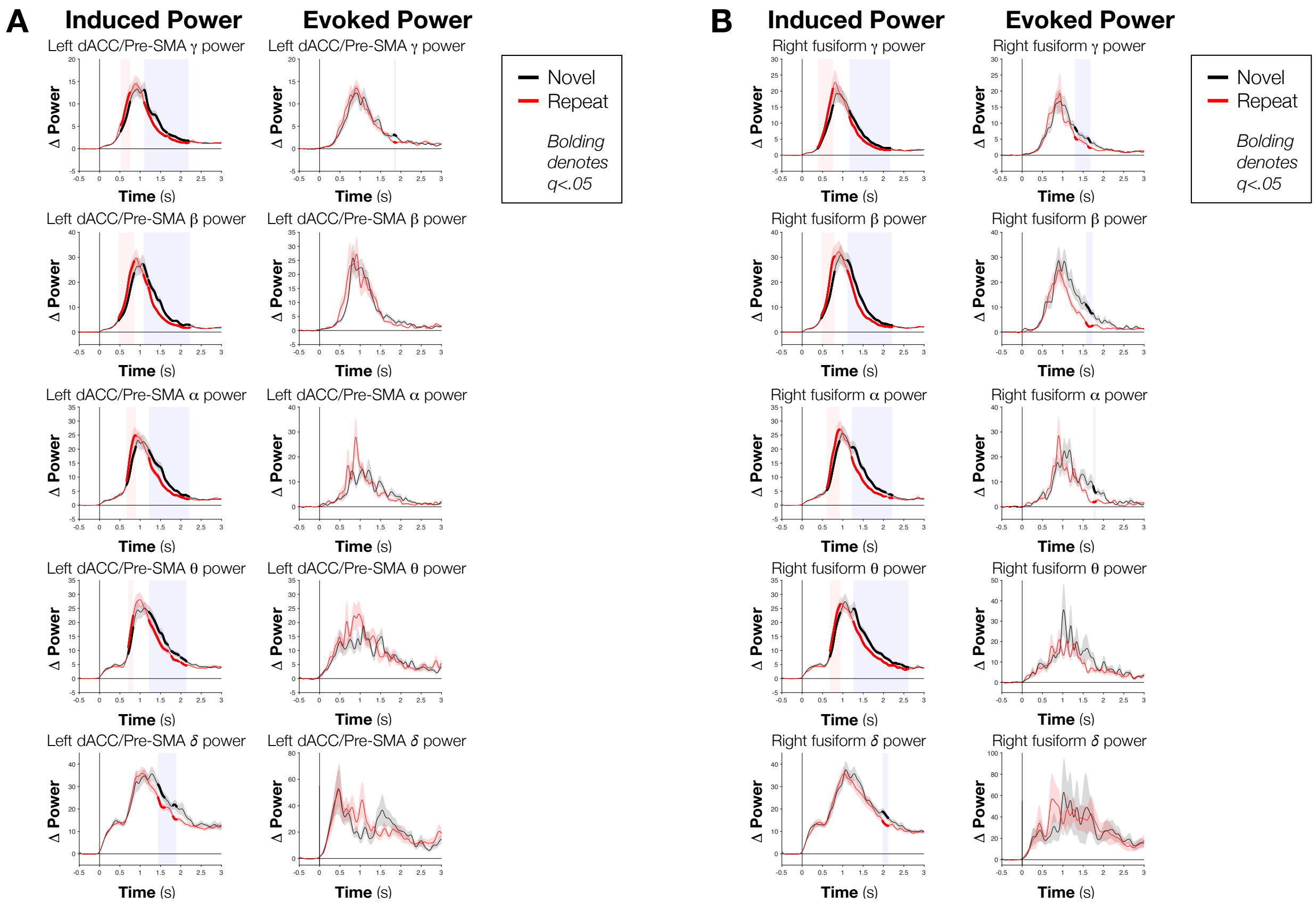

Supplementary Fig. 2. Effects of repeated object identification on induced versus evoked power for the fMRI-defined A) dorsal anterior cingulate/pre-supplementary motor area (dACC/Pre-SMA) and B) right fusiform regions. Evoked power corresponds to power estimates after first averaging across trials in the time domain per condition, whereas induced power is calculated per trial after first subtracting the trial-averaged evoked response and then averaging across trials. FDR-corrected periods are shown with bolded lines, with red lines representing change in power relative to the baseline period for the Repeat condition and black lines representing the Novel condition.

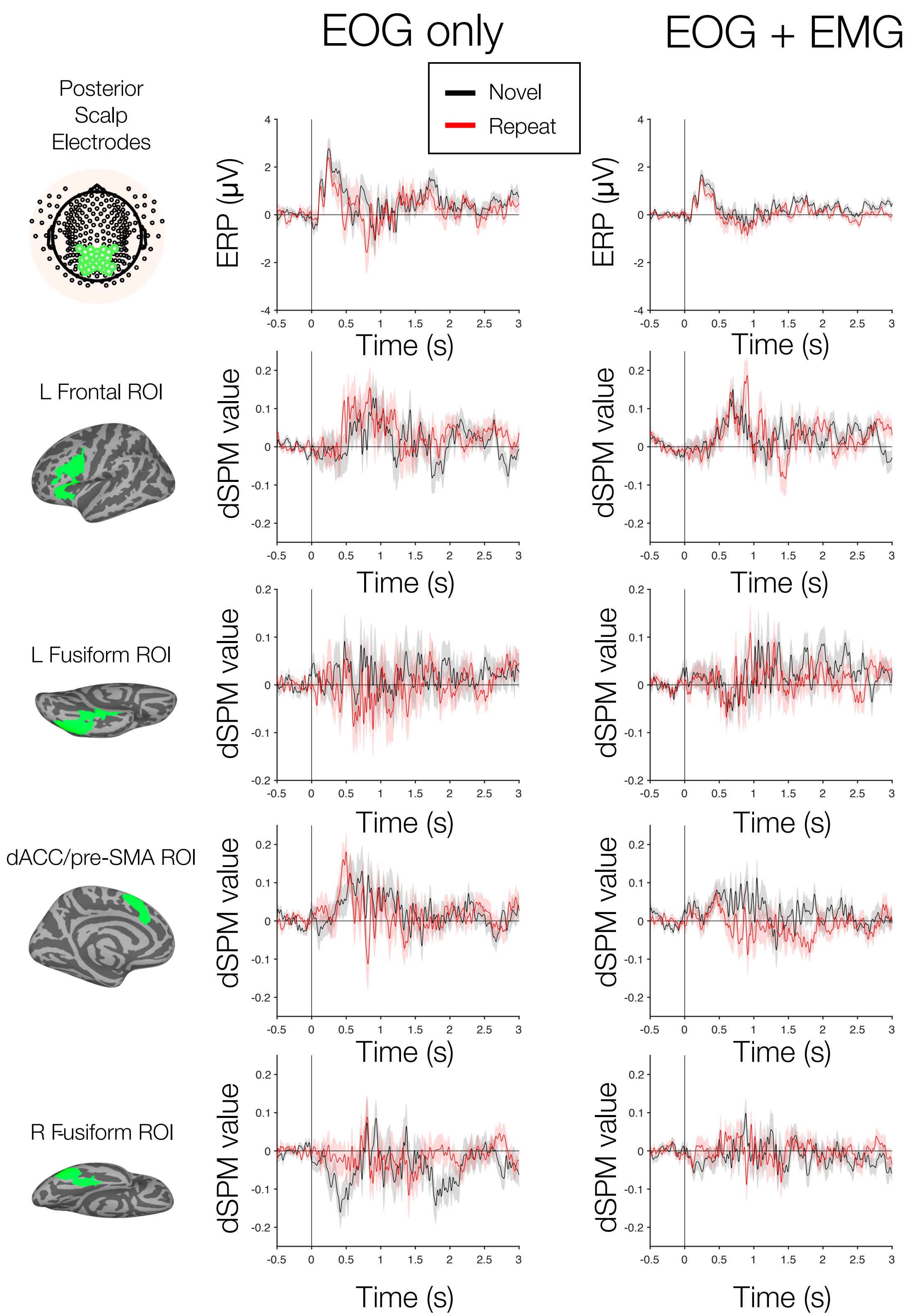

Supplementary Fig. 3. Event related potentials for EOG only (pipeline 1) versus EOG plus speech EMG pre-processing (pipeline 2). Novel and Repeat condition ERPs (black and red lines,

respectively) are shown in the top row for the selection of posterior scalp electrodes (graphic on left), then for the source estimated time series of the fMRI-defined regions (left frontal, left fusiform, left dACC/pre-SMA, and right fusiform regions). The amplitudes of the ERPs are reduced near the time of the average response time (~ .9 seconds) and .5 seconds before for the EOG+EMG pre-processing relative to the EOG only pre-processing. The estimated ERPs are unitless for the fMRI-defined regions due to the normalization by noise covariance periods.

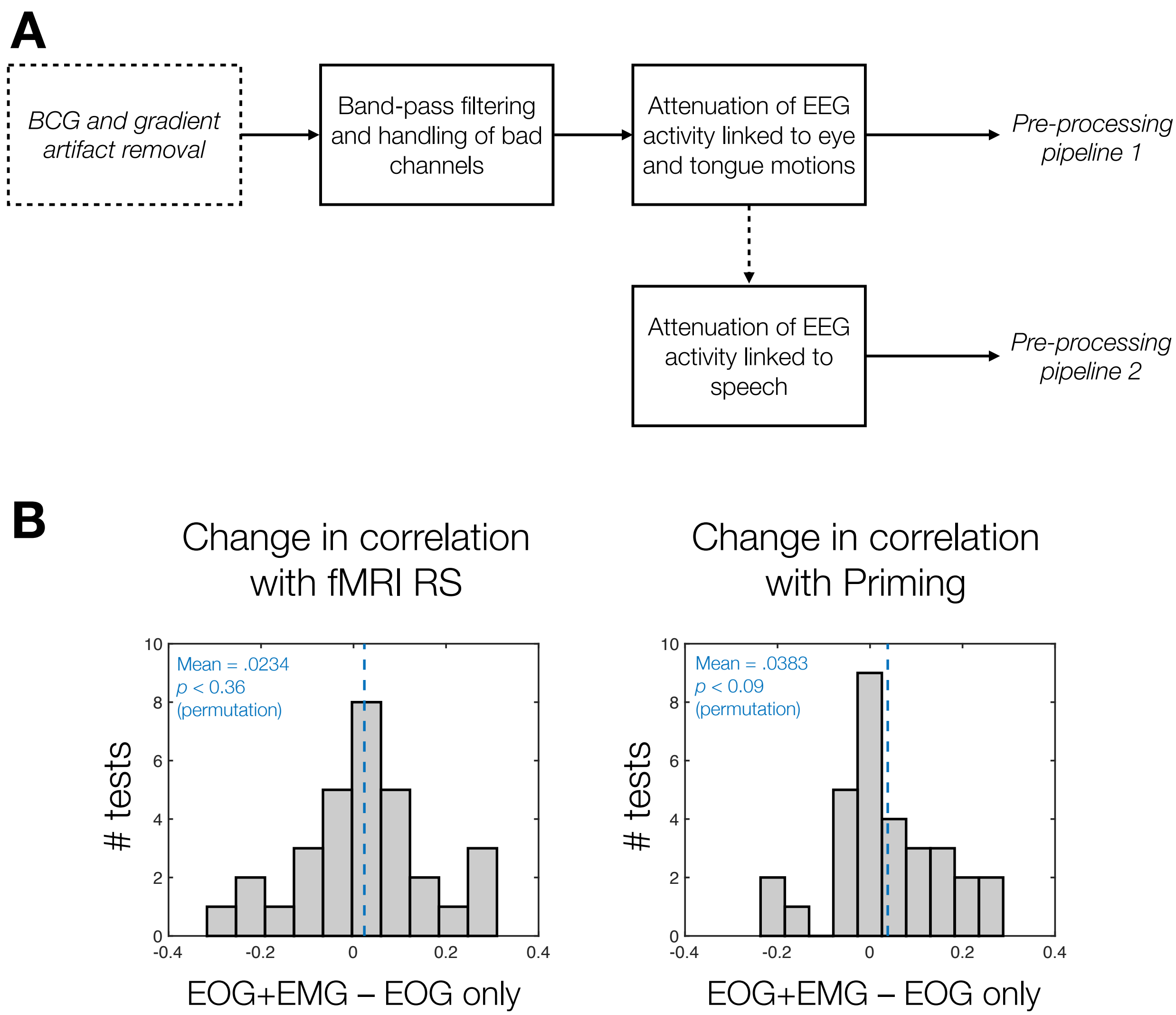

Supplementary Fig. 4. Schematic of different pre-processing pipeline and how these impact correlations of induced power differences (Repeat - Novel) for the early and late periods with fMRI RS and priming. A) Pipeline 1 ("EOG only") regressed signals from electrodes near the eyes and upper jaw to remove EOG and tongue EMG signals, whereas Pipeline 2 ("EOG+EMG") additionally removed signals from the scalp electrodes based on ICAs calculated near the time of the speech response (-0.2 to +0.2 s relative to the RT). See Materials and Methods: EEG Preprocessing for further details. B) Correlations of induced power differences (Repeat - Novel) for the early and late periods with fMRI RS and priming show little change or slightly larger correlations in the EOG+EMG preprocessing (pipeline 2) relative to the EOG only preprocessing (pipeline 1). Shown are frequency histograms of the change in correlation between pipelines across all tests, with mean change values near zero for correlations with fMRI RS and marginally positive for correlations with priming ($p < .09$, permutation test). Negative correlations were first rectified to positive values using absolute value for the purposes of these calculations (e.g. positive values in the histogram reflect a stronger correlation – either originally positive or negative – in the EOG+EMG relative to the EOG only preprocessing). Permutation

testing (10,000 iterations) was conducted by randomly flipping the signs of the correlation differences and recalculating the mean differences.

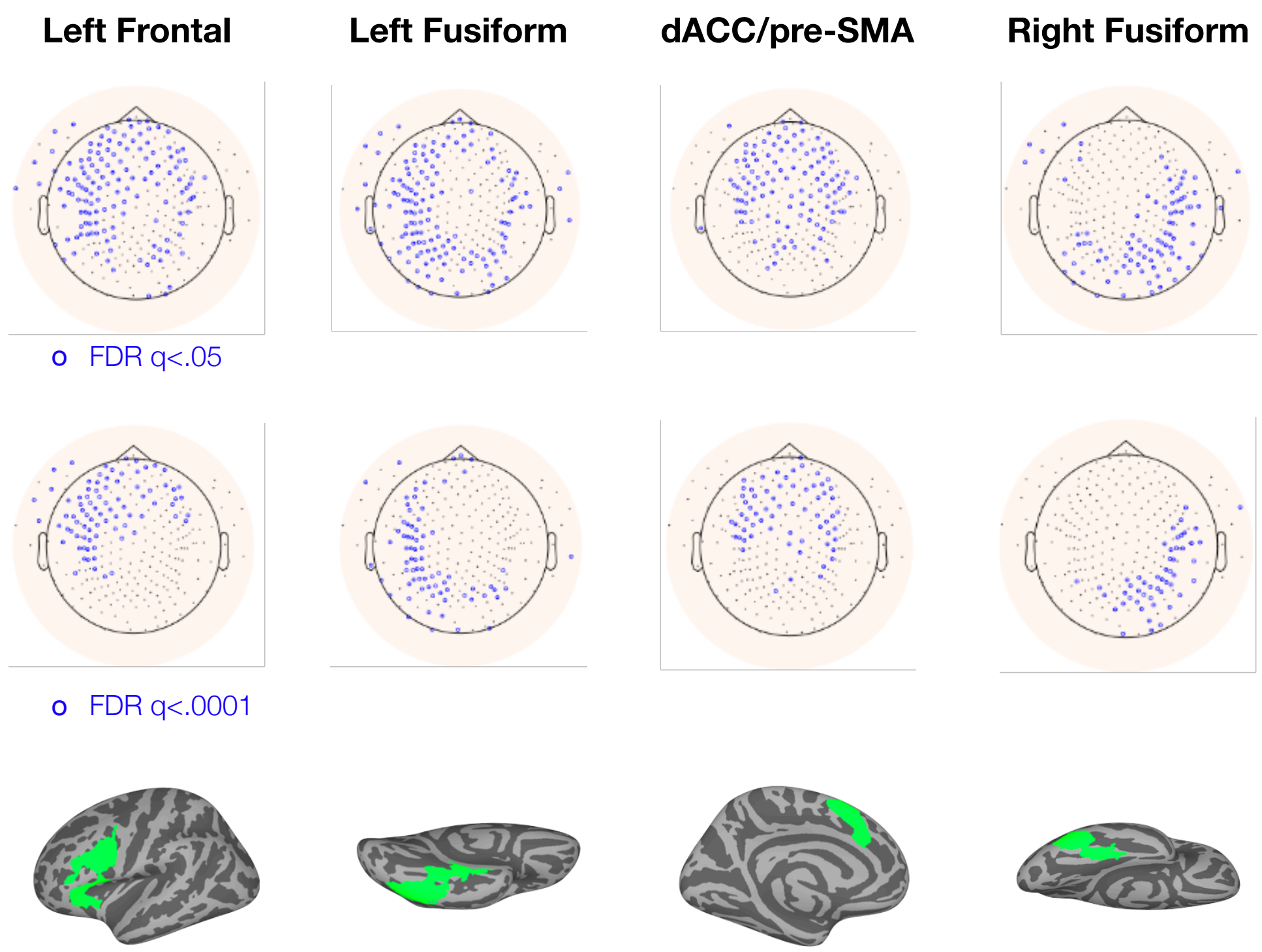

Supplementary Fig. 5. Mapping source regions to their most heavily weighted scalp electrodes. Shown are two FDR-corrected significance levels: FDR $q < .05$ (minimum thresholding for significance) and $q < .0001$ (more stringent thresholding). Some degree of spatial selectivity is observed under both thresholds, although even at the more stringent threshold ($q < .0001$) there is overlap of the identified scalp electrodes across regions – highlighting the more limited spatial resolution of EEG. Electrodes were identified by taking the electrode weights associated with each source vertex in the source estimation process (see Materials and Methods), normalizing them by the standard deviation across those weights, and then finding the mean weight with each surface electrode for all the vertices belonging to a given fMRI-defined region. One-sample *t*-tests across participants were then carried out for each scalp electrode per fMRI-defined region. Multiple comparisons were corrected by FDR. ERPs and induced power differences for these electrode selections are shown in Supplementary Figs. 6-9. While induced power differences appear similar across frequencies and fMRI-defined regions, the ERPs for these electrode selections have distinctive shapes, consistent with a moderate degree of spatial selectivity of the scalp EEG signals.

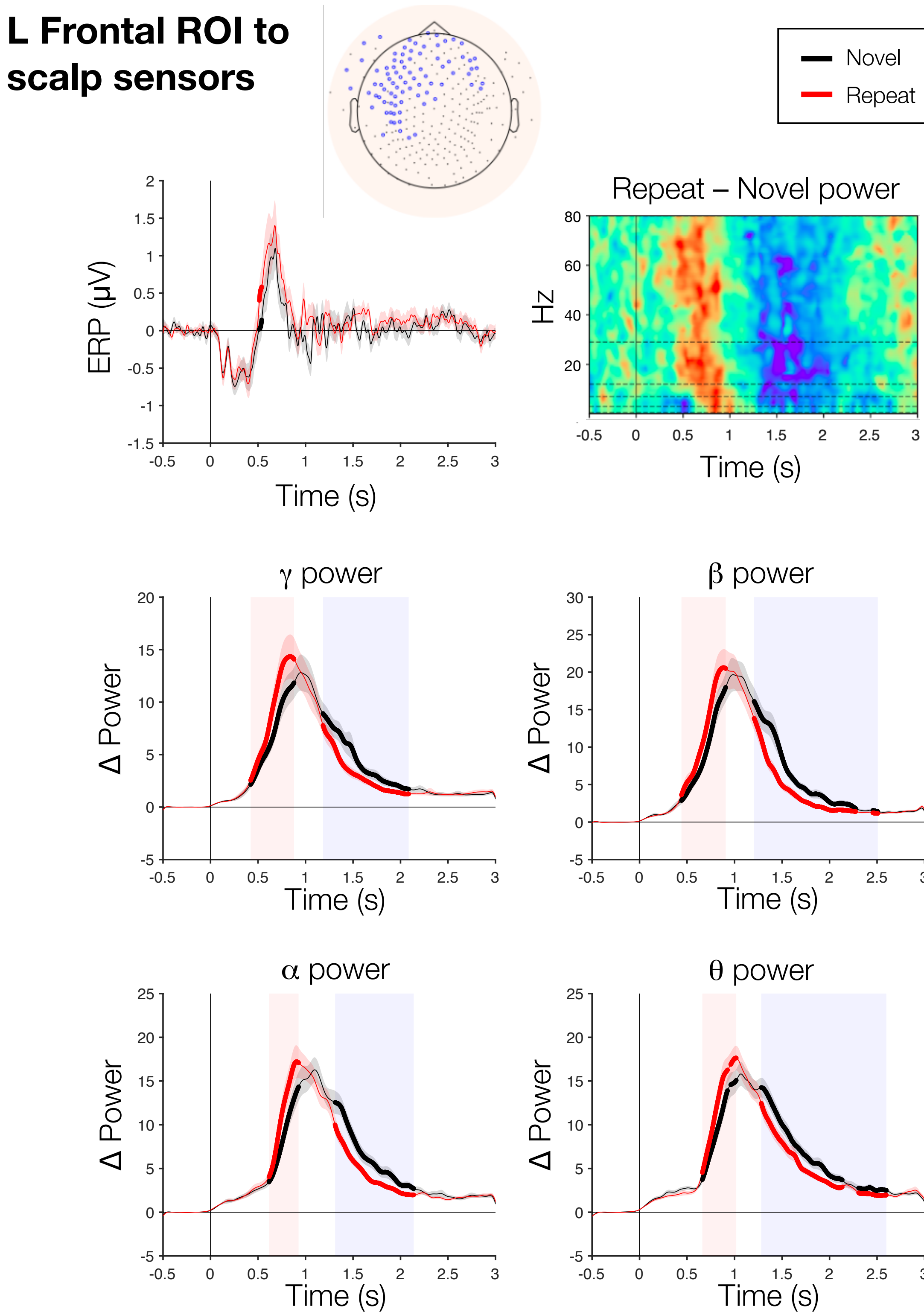

Supplementary Fig. 6. Companion scalp electrode analyses for the fMRI-defined left frontal region (FDR $q < .0001$ from Supplementary Fig. 5). Average ERPs for the defined collection of electrodes (see inset at top) for the Novel (black) and Repeat (red) conditions. Repeat-Novel time

by frequency induced power differences are shown in the upper right (paired *t*-tests across participants), highlighting the same “early” (Repeat > Novel) and “late” (Novel > Repeat) periods as in the source-estimated results in Fig. 2. Banded power analyses (Novel vs. Repeat) are then shown below for the gamma (30-80 Hz), beta (13-29 Hz), alpha (8-12 Hz) and theta (4-7 Hz) frequency bands, with FDR-corrected periods of significance indicated with bolded lines. While no FDR-corrected periods were detected in the ERPs, robust induced power differences are observed across frequencies.

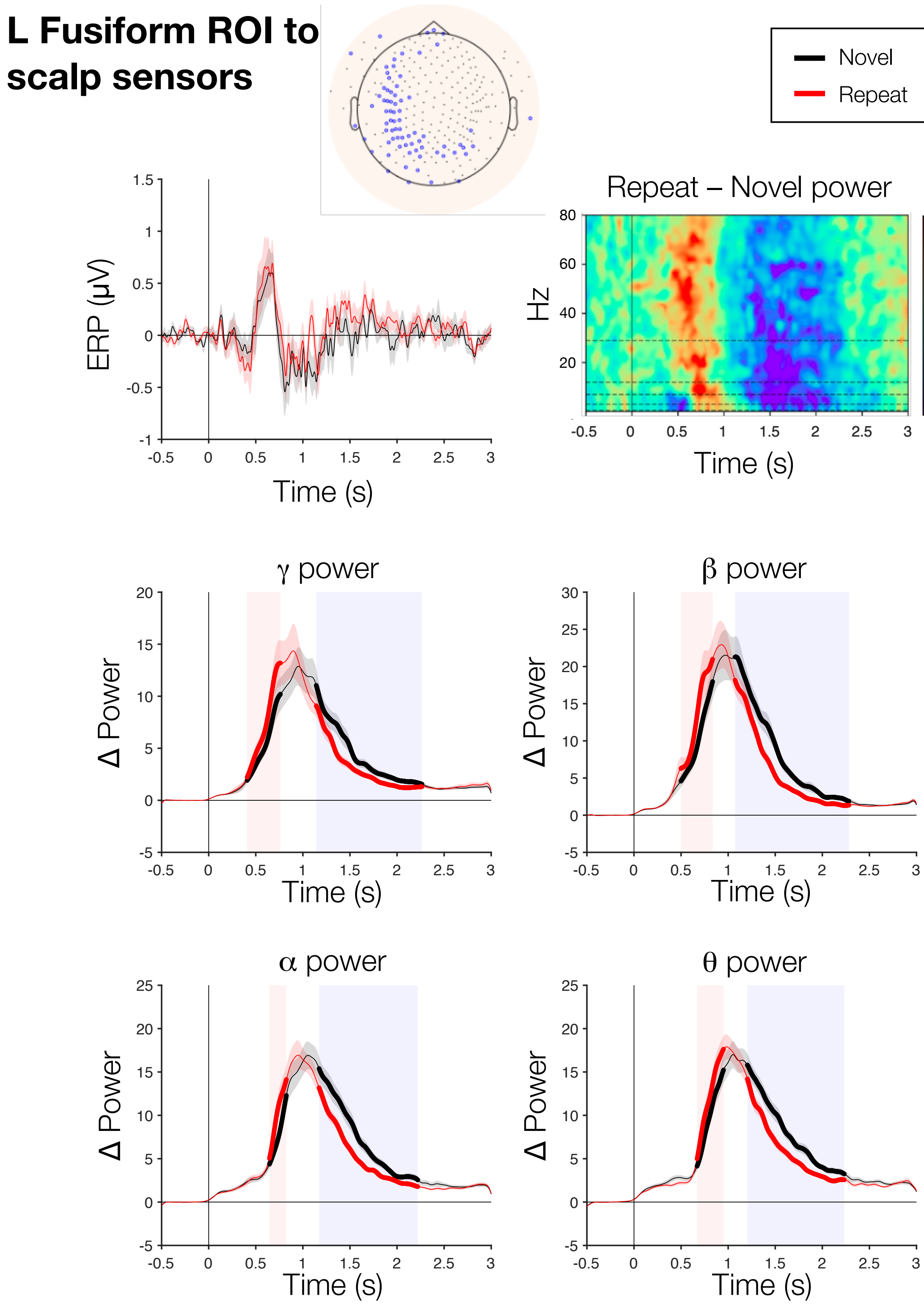

Supplementary Fig. 7. Companion scalp electrode analyses for the fMRI-defined left fusiform region (FDR $q < .0001$ from Supplementary Fig. 5). Average ERPs for the defined collection of electrodes (see inset at top) for the Novel (black) and Repeat (red) conditions. Repeat-Novel time

by frequency induced power differences are shown in the upper right (paired *t*-tests across participants), highlighting the same “early” (Repeat>Novel) and “late” (Novel>Repeat) periods as in the source-estimated results in Fig. 2. Banded power analyses (Novel vs Repeat) are then shown below for the gamma (30-80 Hz), beta (13-29 Hz), alpha (8-12 Hz) and theta (4-7 Hz) frequency bands, with FDR-corrected periods of significance indicated with bolded lines. While no FDR-corrected periods were detected in the ERPs, robust induced power differences are observed across frequencies.

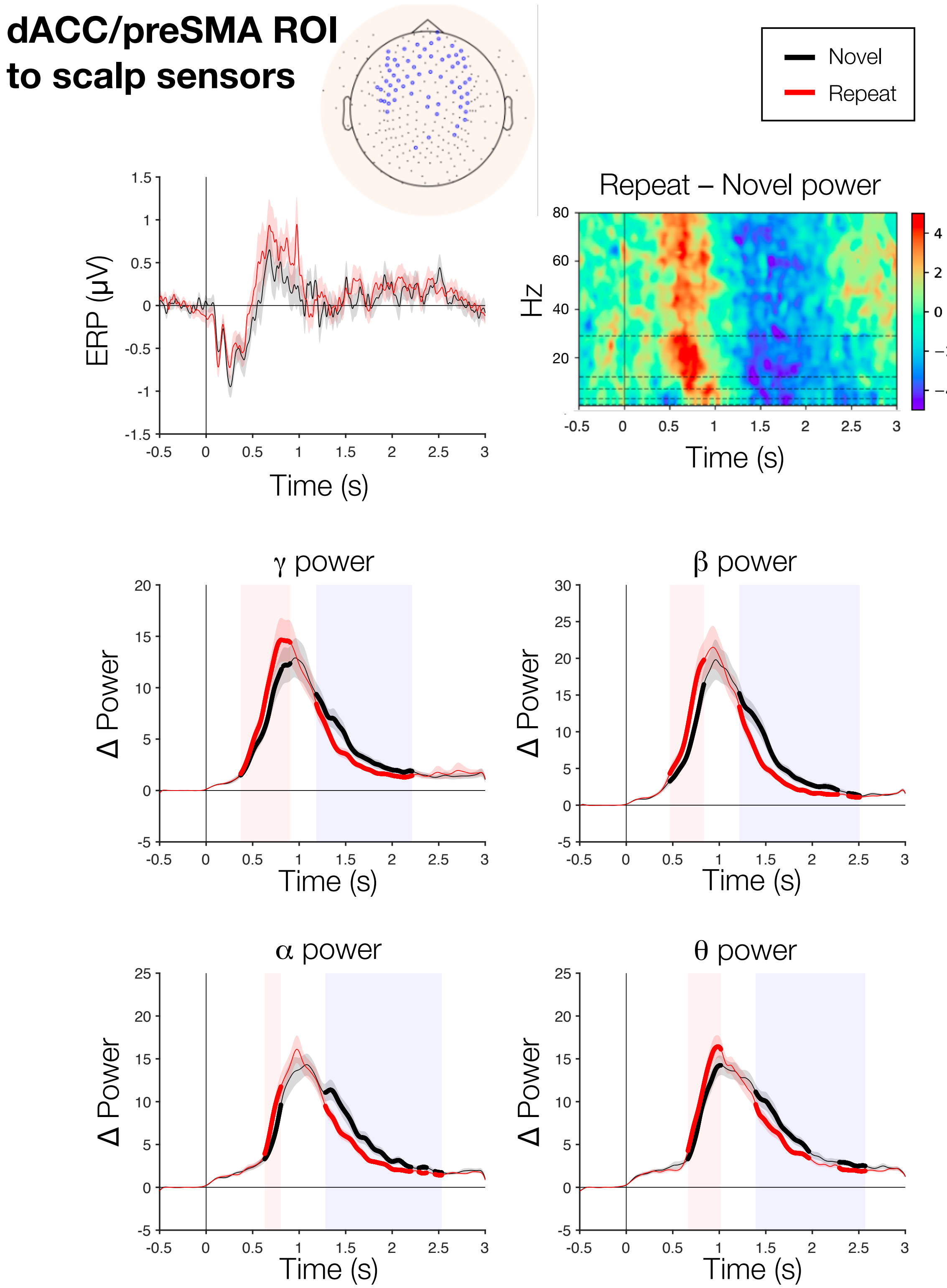

Supplementary Fig. 8. Companion scalp electrode analyses for the fMRI-defined left dACC/pre-SMA region (FDR $q < .0001$ from Supplementary Fig. 5). Average ERPs for the defined collection of electrodes (see inset at top) for the Novel (black) and Repeat (red)

conditions. Repeat-Novel time by frequency induced power differences are shown in the upper right (paired *t*-tests across participants), highlighting the same “early” (Repeat>Novel) and “late” (Novel>Repeat) periods as in the source-estimated results in Fig. 2. Banded power analyses (Novel vs Repeat) are then shown below for the gamma (30-80 Hz), beta (13-29 Hz), alpha (8-12 Hz) and theta (4-7 Hz) frequency bands, with FDR-corrected periods of significance indicated with bolded lines. While no FDR-corrected periods were detected in the ERPs, robust induced power differences are observed across frequencies.

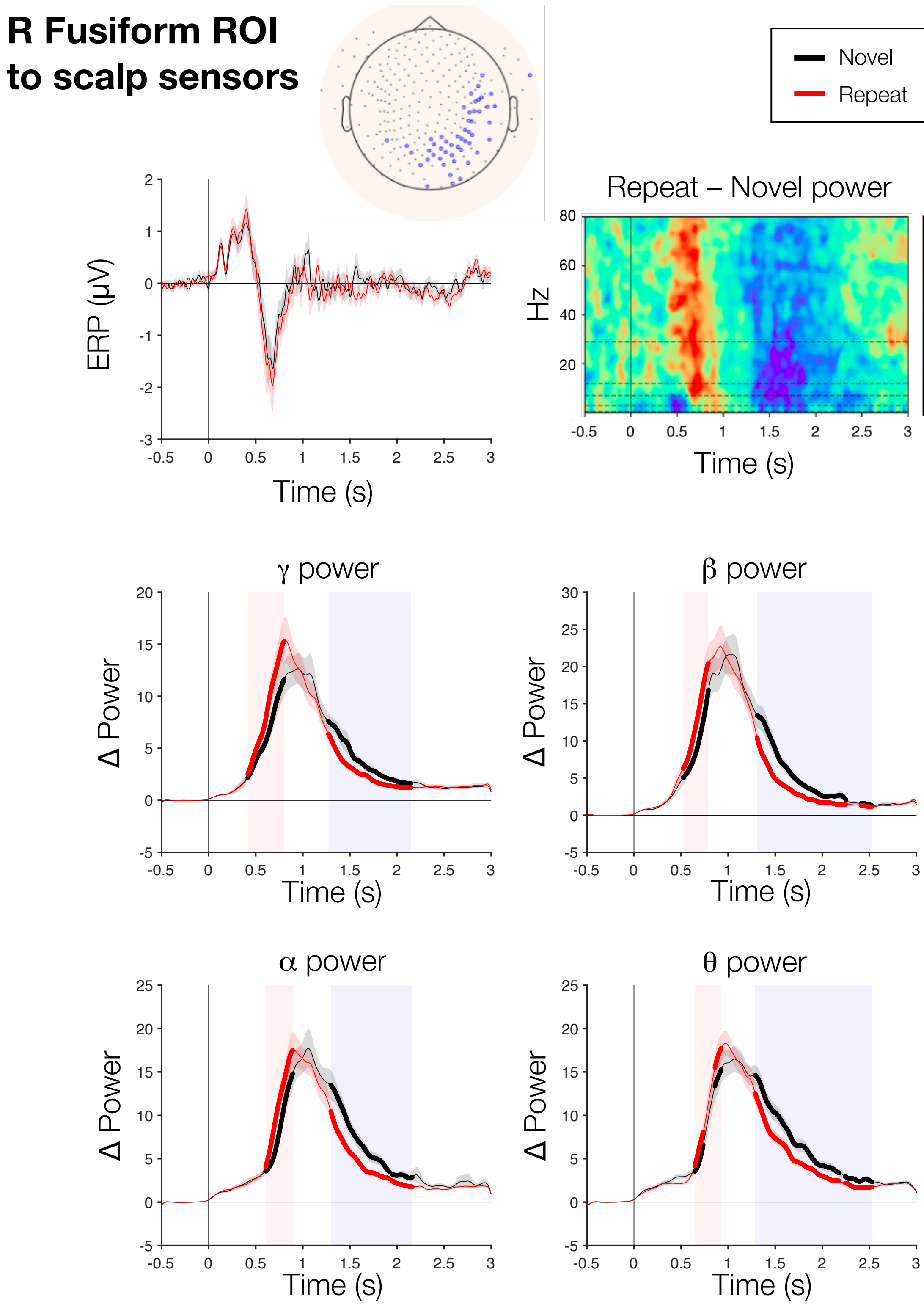

Supplementary Fig. 9. Companion scalp electrode analyses for the fMRI-defined right fusiform region (FDR *q* <.0001 from Supplementary Fig. 5). Average ERPs for the defined collection of electrodes (see inset at top) for the Novel (black) and Repeat (red) conditions. Repeat-Novel time

by frequency induced power differences are shown in the upper right (paired *t*-tests across participants), highlighting the same “early” (Repeat>Novel) and “late” (Novel>Repeat) periods as in the source-estimated results in Fig. 2. Banded power analyses (Novel vs Repeat) are then shown below for the gamma (30-80 Hz), beta (13-29 Hz), alpha (8-12 Hz) and theta (4-7 Hz) frequency bands, with FDR-corrected periods of significance indicated with bolded lines. While no FDR-corrected periods were detected in the ERPs, robust induced power differences are observed across frequencies.

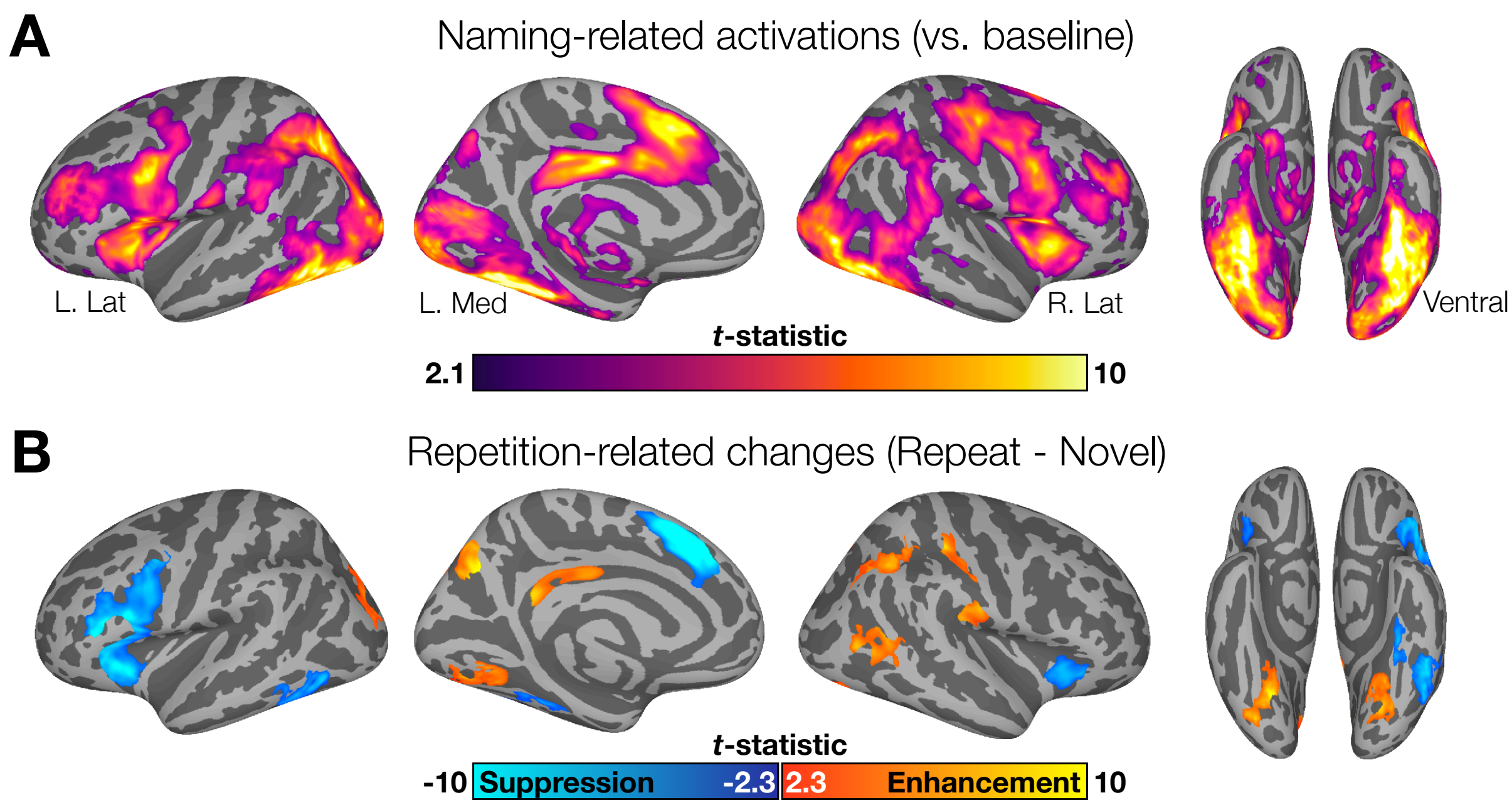

Supplementary Fig. 10. Vertexwise results of Naming-related activations and Repetition effects in the covert naming experiment. (A) fMRI identified significant activations in multiple regions across the cortex, consistent with those observed in the overt naming task. (B) Within Naming-activated regions, significant repetition suppression was noted in frontal and fusiform cortex and were generally left-lateralized. Effects are somewhat attenuated when compared to those in the Overt data, potentially due a combination of the covert nature of the task and the smaller sample size. For display purposes, the map of repetition effects is set to $q < .05$, inclusively masked by regions identified in the earlier Naming-related activation map, while maintaining the same cluster size minimum.

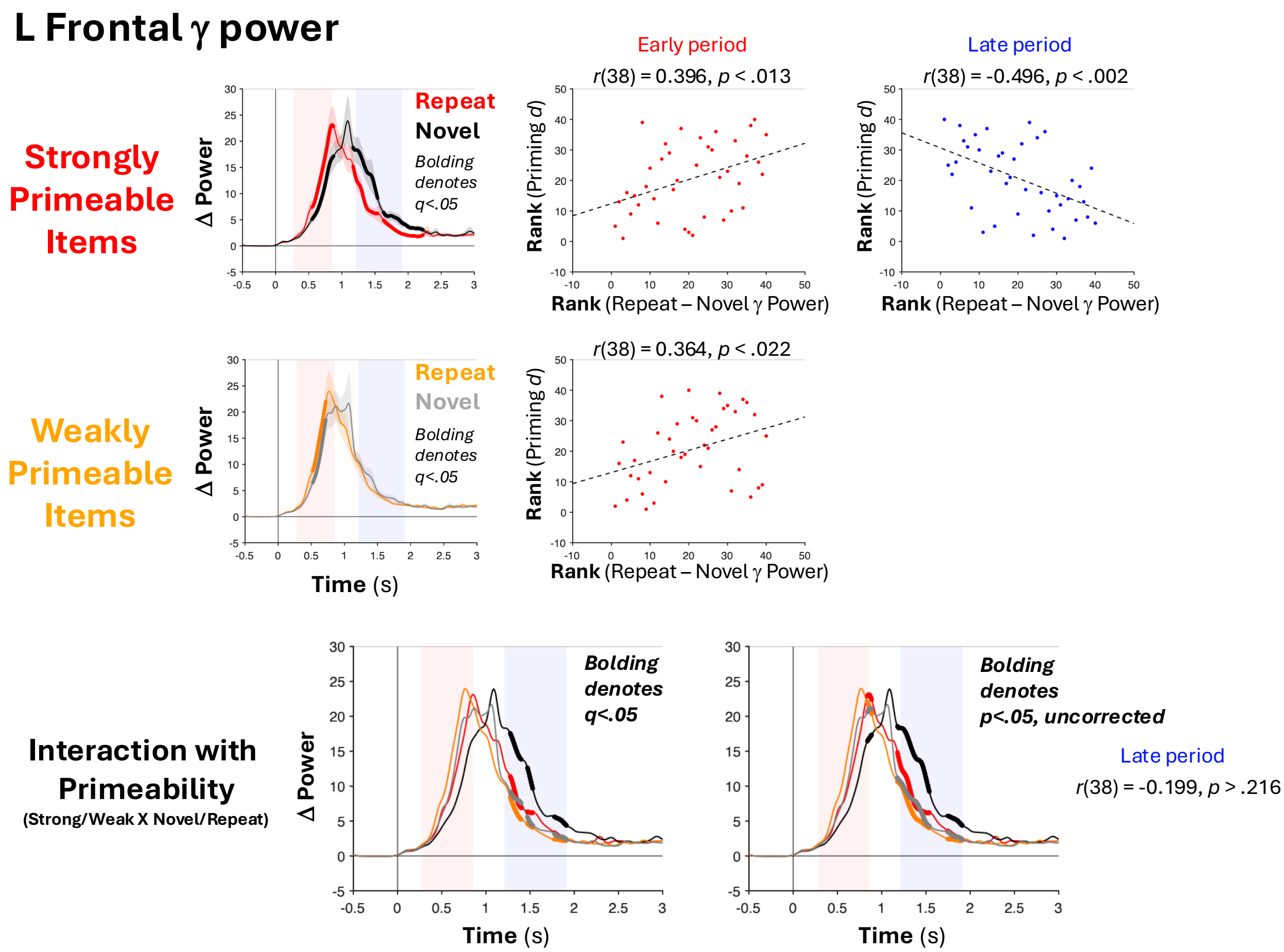

Supplementary Fig. 11. Left frontal ROI gamma power for novel and repeat stimuli, plotted separately for Strongly primeable (top row) and Weakly primeable (middle row) items. Results of a test for interactions between primeability (strong/weak) and novel/repeat status are displayed in the bottom row. Bolded lines reflect FDR-corrected differences (top 2 rows) or interactions (bottom row). When appropriate, correlations (Spearman) between behavioral priming and differences in EEG power during “early” and “late” periods are plotted; correlation results are otherwise noted but not displayed for periods of significance. Light background colors reflect periods of significant difference that were identified in earlier analyses that did not separate novel and repeat trials by primeability.

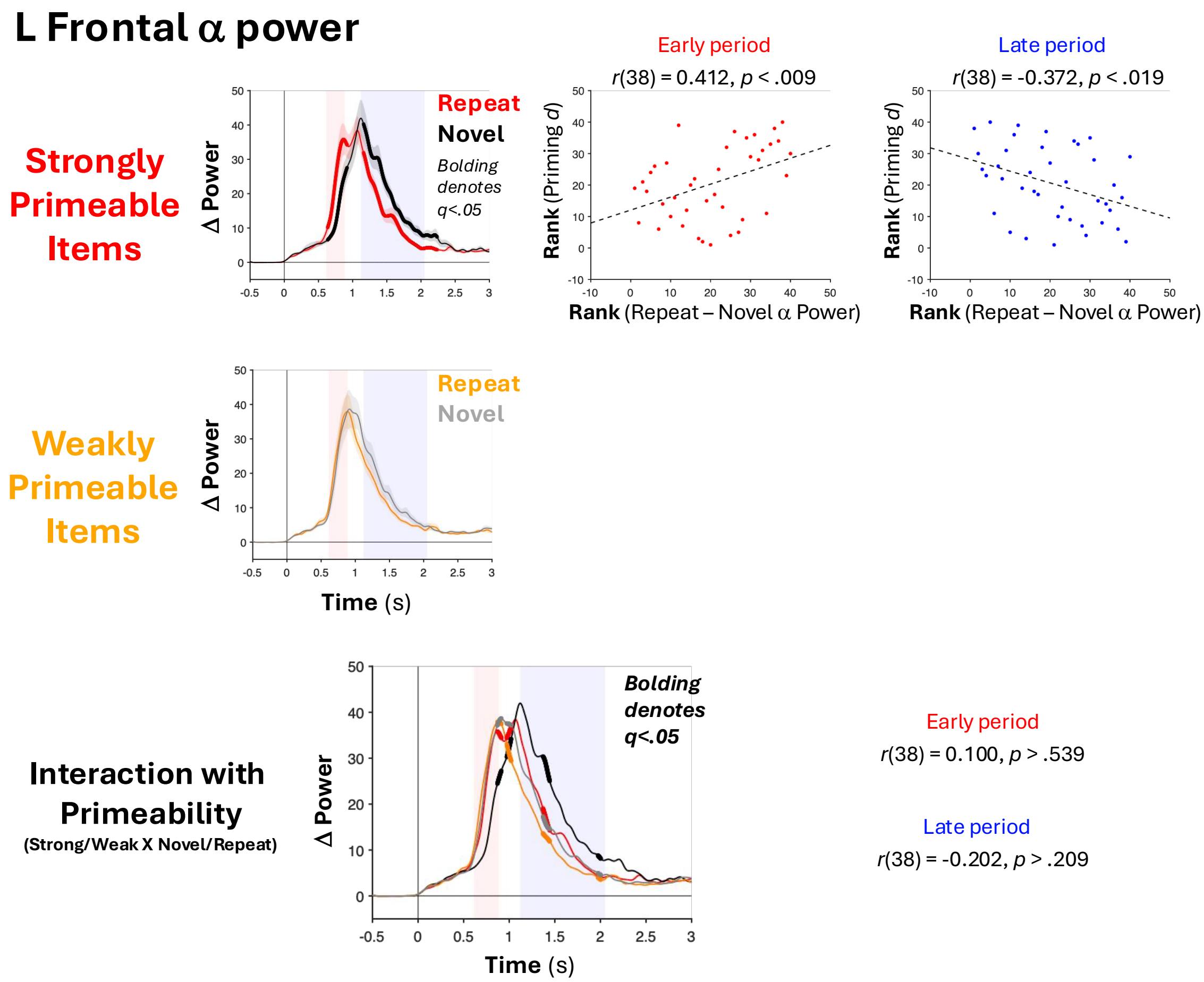

Supplementary Fig. 12. Left frontal ROI alpha power for novel and repeat stimuli, plotted separately for Strongly primeable (top row) and Weakly primeable (middle row) items. Results of a test for interactions between primeability (strong/weak) and novel/repeat status are displayed in the bottom row. Bolded lines reflect FDR-corrected differences (top 2 rows) or interactions (bottom row). When significant, correlations (Spearman) between behavioral priming and differences in EEG power during "early" and "late" periods are plotted; correlation results are otherwise noted but not displayed for periods of significance. Light background colors reflect periods of significant difference that were identified in earlier analyses that did not separate novel and repeat trials by primeability.

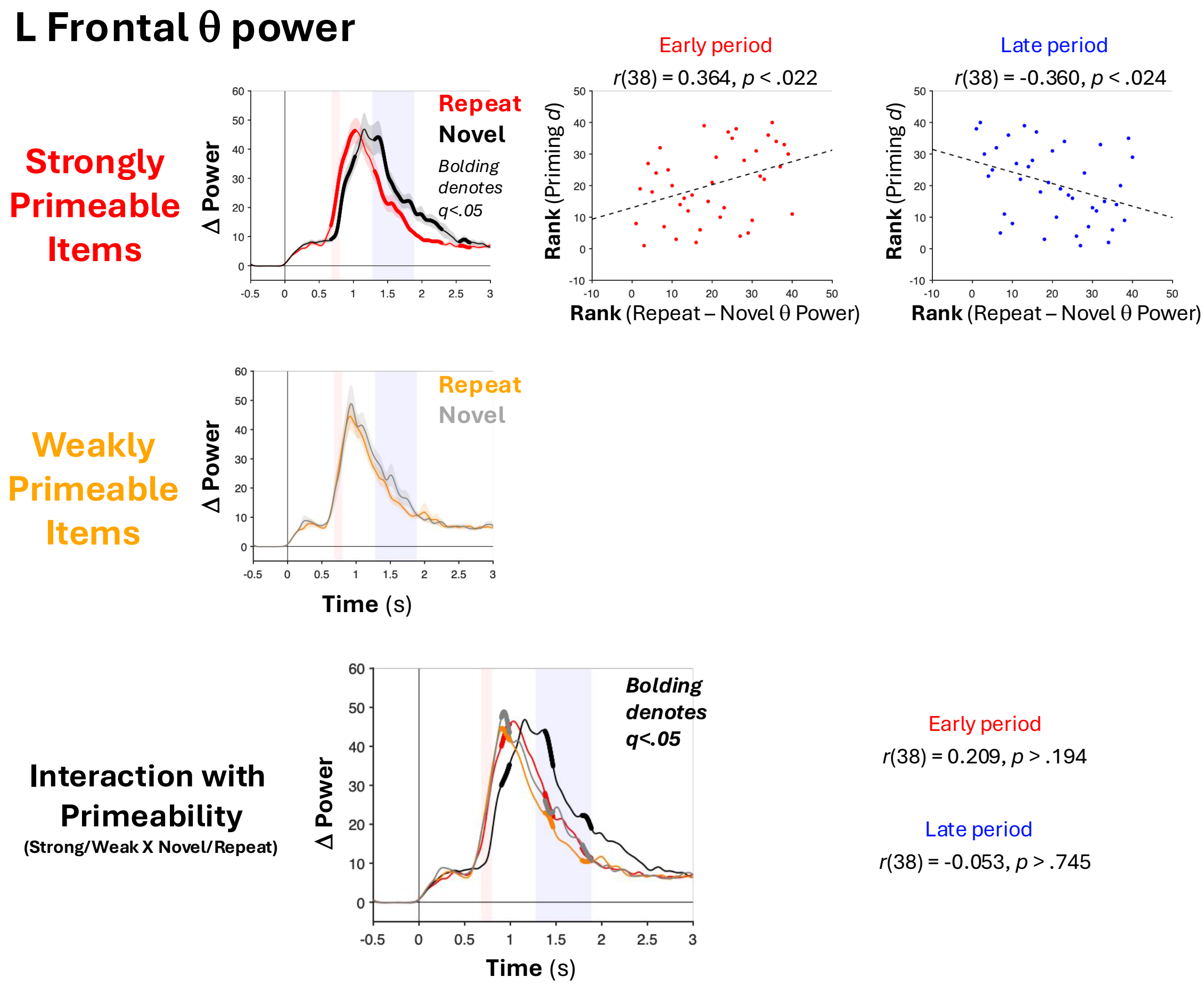

Supplementary Fig. 13. Left frontal ROI theta power for novel and repeat stimuli, plotted separately for Strongly primeable (top row) and Weakly primeable (middle row) items. Results of a test for interactions between primeability (strong/weak) and novel/repeat status are displayed in the bottom row. Bolded lines reflect FDR-corrected differences (top 2 rows) or interactions (bottom row). When significant, correlations (Spearman) between behavioral priming and differences in EEG power during "early" and "late" periods are plotted; correlation results are otherwise noted but not displayed for periods of significance. Light background colors reflect periods of significant difference that were identified in earlier analyses that did not separate novel and repeat trials by primeability.

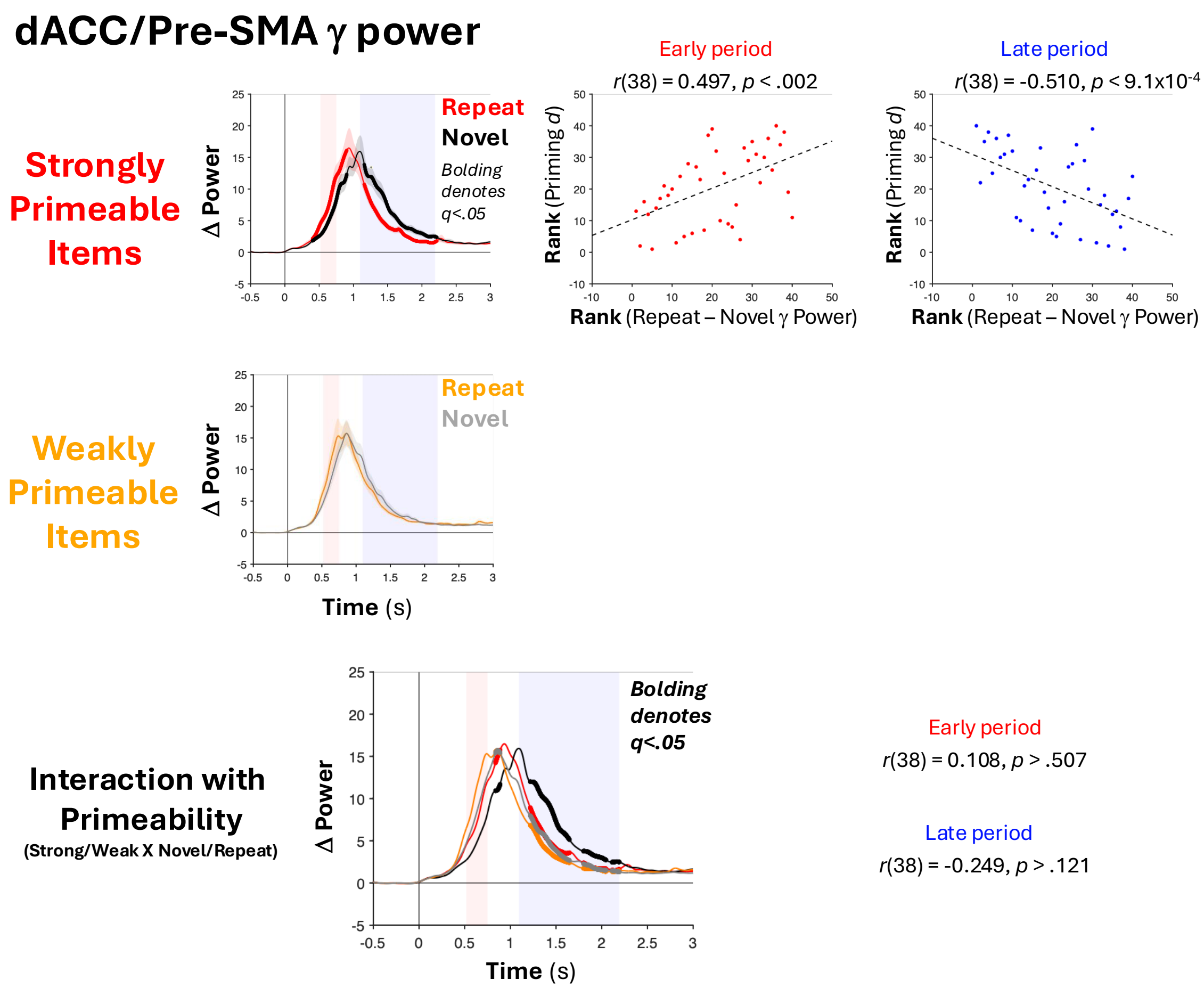

Supplementary Fig. 14. dACC/Pre-SMA ROI gamma power for novel and repeat stimuli, plotted separately for Strongly primeable (top row) and Weakly primeable (middle row) items. Results of a test for interactions between primeability (strong/weak) and novel/repeat status are displayed in the bottom row. Bolded lines reflect FDR-corrected differences (top 2 rows) or interactions (bottom row). When significant, correlations (Spearman) between behavioral priming and differences in EEG power during "early" and "late" periods are plotted; correlation results are otherwise noted but not displayed for periods of significance. Light background colors reflect periods of significant difference that were identified in earlier analyses that did not separate novel and repeat trials by primeability.

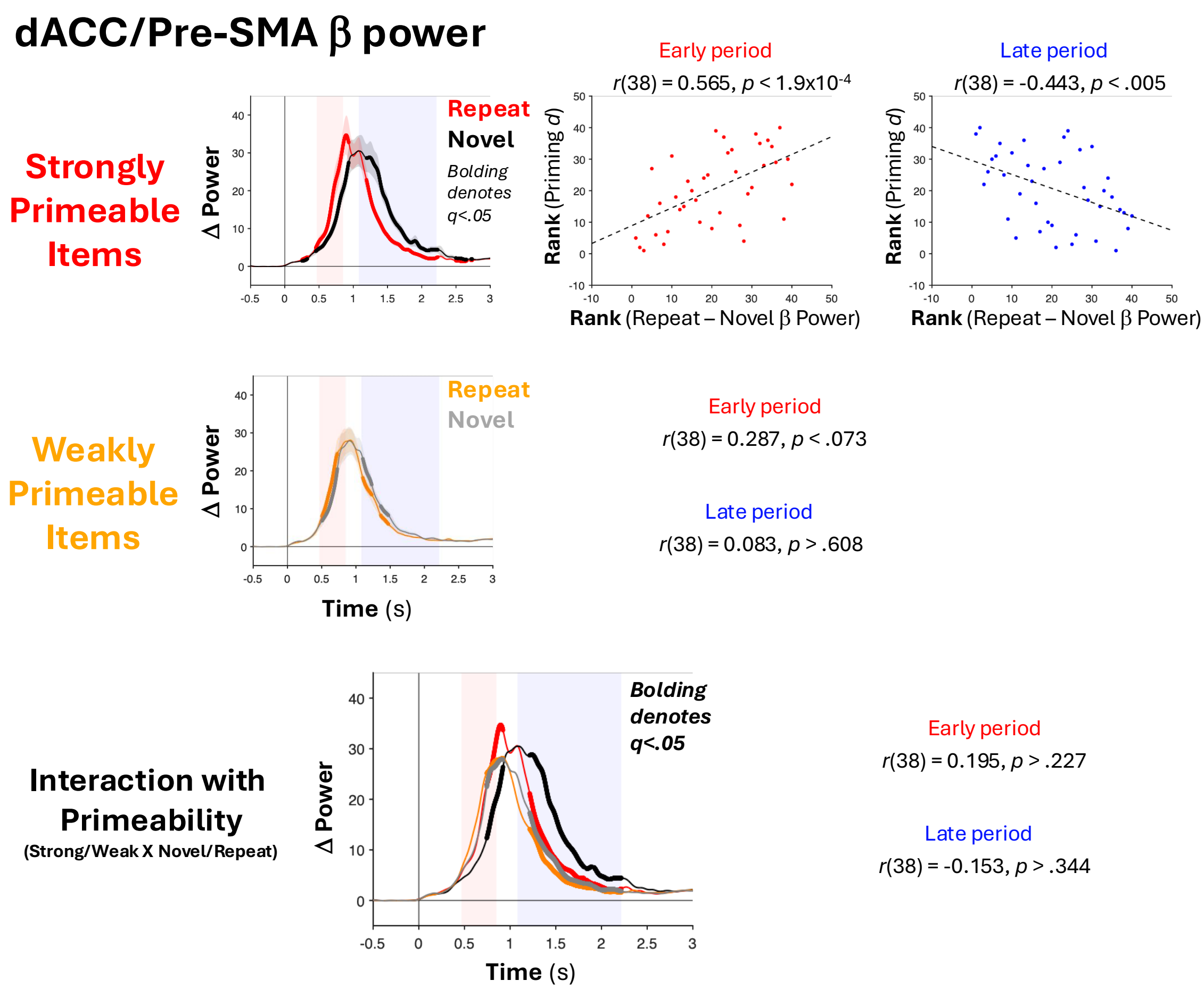

Supplementary Fig. 15. dACC/Pre-SMA ROI beta power for novel and repeat stimuli, plotted separately for Strongly primeable (top row) and Weakly primeable (middle row) items. Results of a test for interactions between primeability (strong/weak) and novel/repeat status are displayed in the bottom row. Bolded lines reflect FDR-corrected differences (top 2 rows) or interactions (bottom row). When significant, correlations (Spearman) between behavioral priming and differences in EEG power during "early" and "late" periods are plotted; correlation results are otherwise noted but not displayed for periods of significance. Light background colors reflect periods of significant difference that were identified in earlier analyses that did not separate novel and repeat trials by primeability.

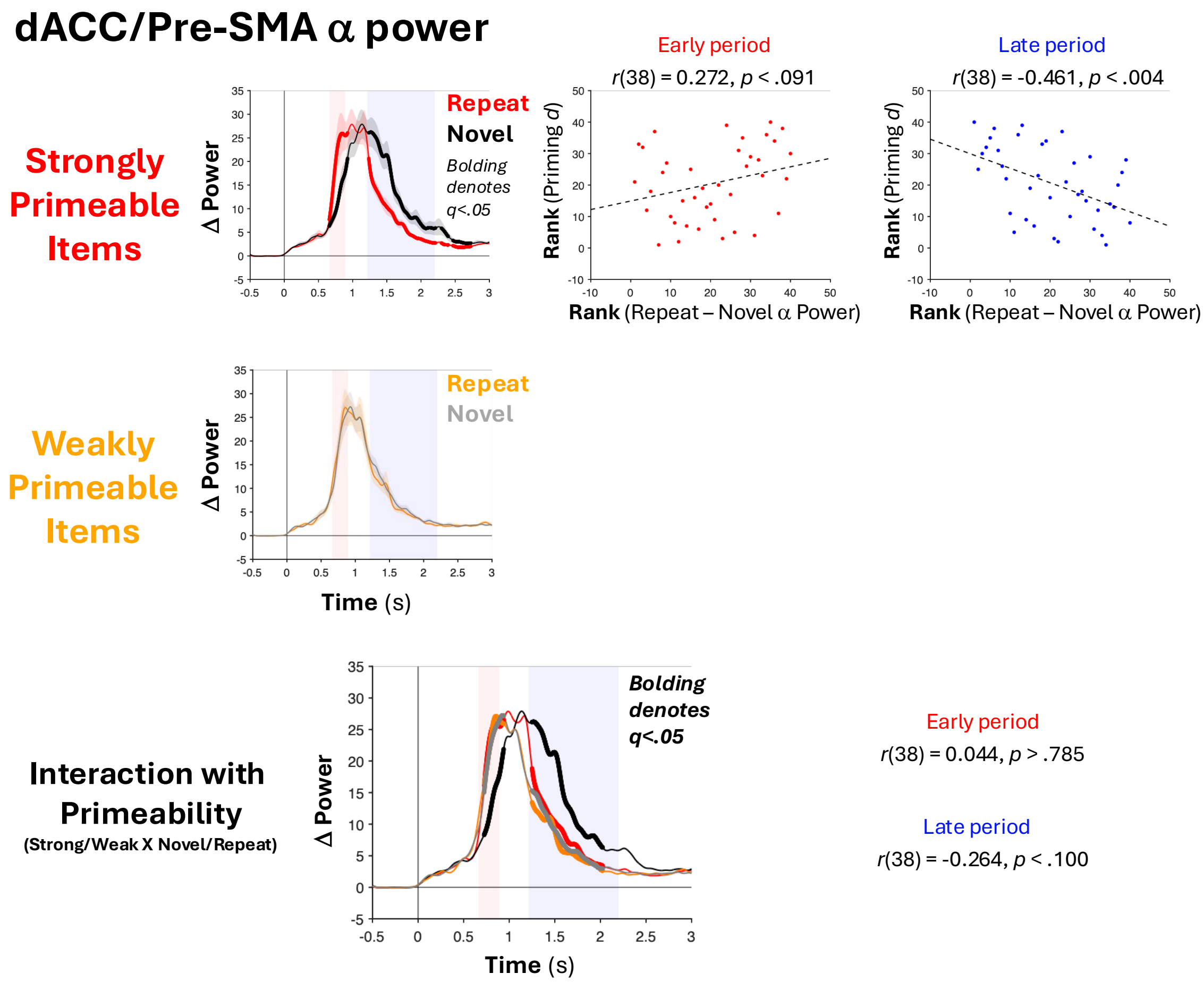

Supplementary Fig. 16. dACC/Pre-SMA ROI alpha power for novel and repeat stimuli, plotted separately for Strongly primeable (top row) and Weakly primeable (middle row) items. Results of a test for interactions between primeability (strong/weak) and novel/repeat status are displayed in the bottom row. Bolded lines reflect FDR-corrected differences (top 2 rows) or interactions (bottom row). When significant, correlations (Spearman) between behavioral priming and differences in EEG power during "early" and "late" periods are plotted; correlation results are otherwise noted but not displayed for periods of significance. Light background colors reflect periods of significant difference that were identified in earlier analyses that did not separate novel and repeat trials by primeability.

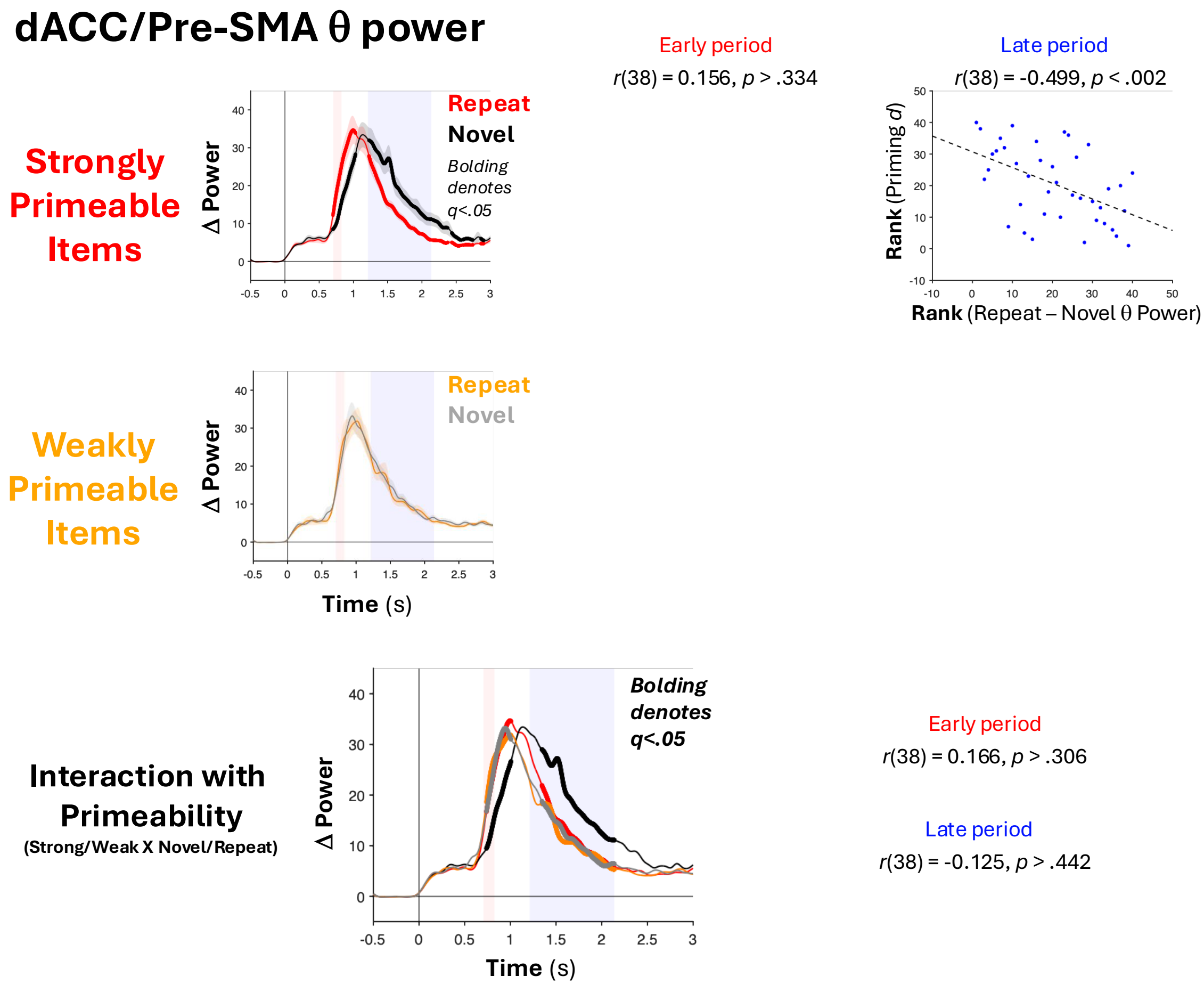

Supplementary Fig. 17. dACC/Pre-SMA ROI theta power for novel and repeat stimuli, plotted separately for Strongly primeable (top row) and Weakly primeable (middle row) items. Results of a test for interactions between primeability (strong/weak) and novel/repeat status are displayed in the bottom row. Bolded lines reflect FDR-corrected differences (top 2 rows) or interactions (bottom row). When significant, correlations (Spearman) between behavioral priming and differences in EEG power during "early" and "late" periods are plotted; correlation results are otherwise noted but not displayed for periods of significance. Light background colors reflect periods of significant difference that were identified in earlier analyses that did not separate novel and repeat trials by primeability.

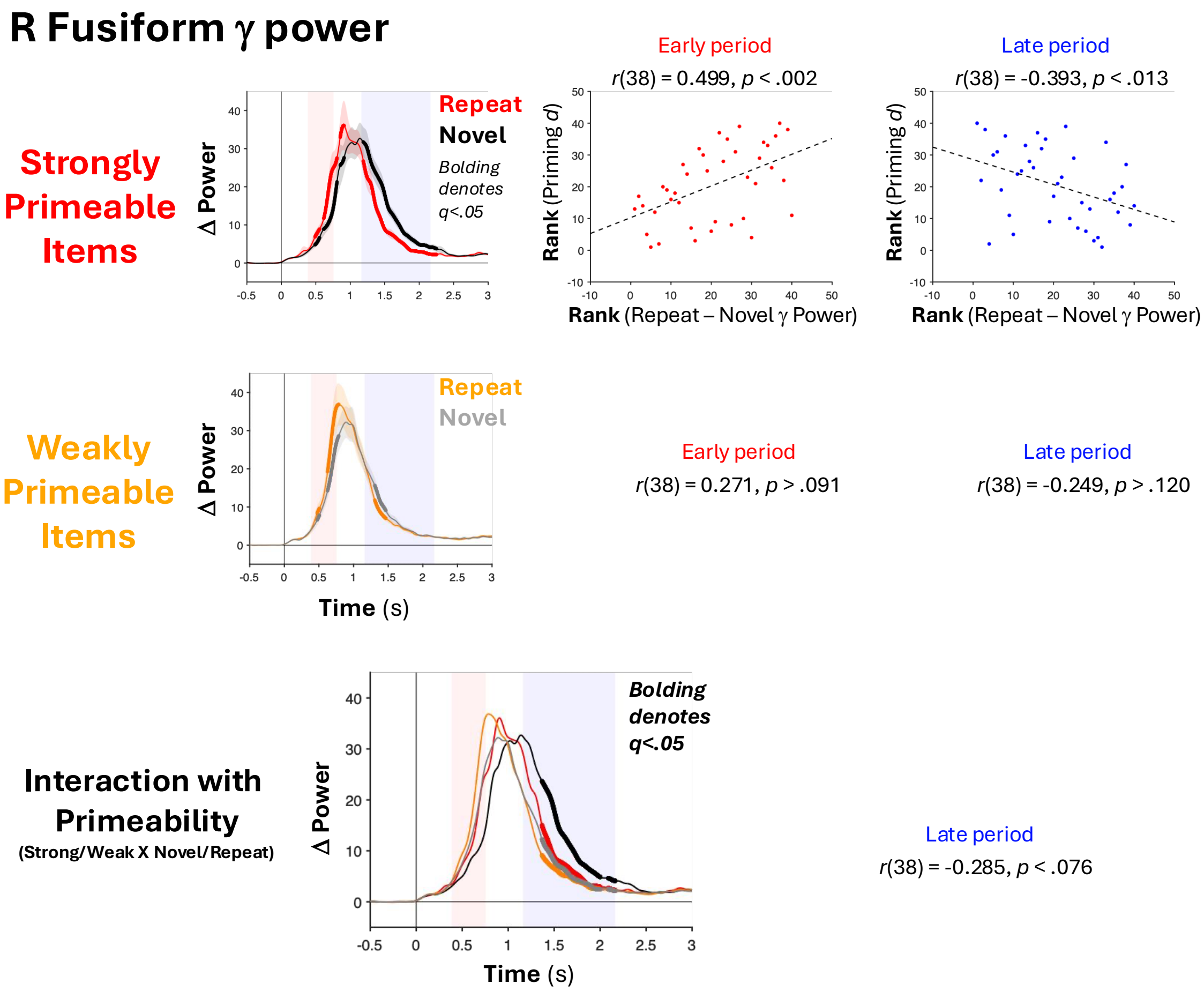

Supplementary Fig. 18. Right fusiform ROI gamma power for novel and repeat stimuli, plotted separately for Strongly primeable (top row) and Weakly primeable (middle row) items. Results of a test for interactions between primeability (strong/weak) and novel/repeat status are displayed in the bottom row. Bolded lines reflect FDR-corrected differences (top 2 rows) or interactions (bottom row). When significant, correlations (Spearman) between behavioral priming and differences in EEG power during “early” and “late” periods are plotted; correlation results are otherwise noted but not displayed for periods of significance. Light background colors reflect periods of significant difference that were identified in earlier analyses that did not separate novel and repeat trials by primeability.

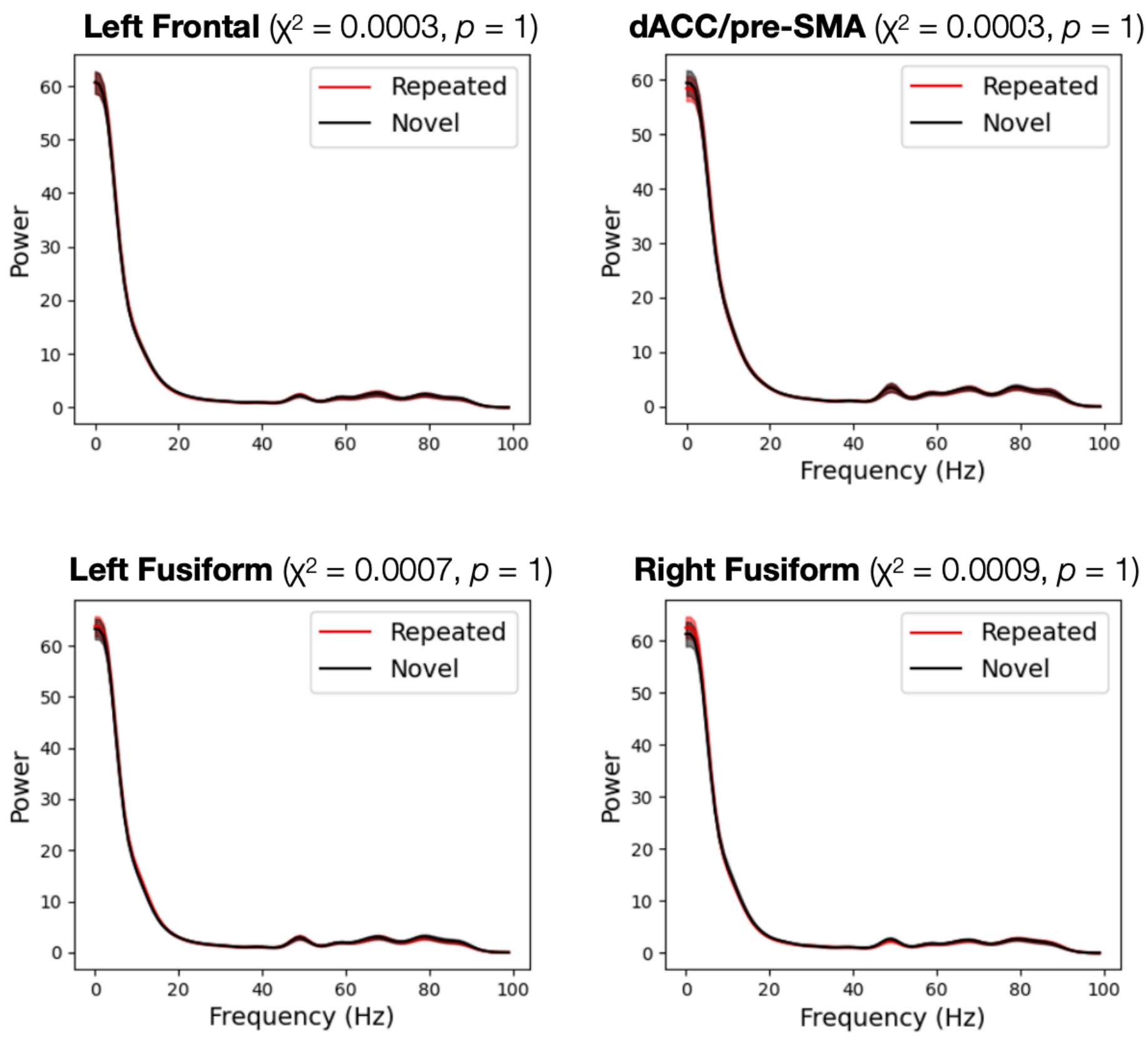

Supplementary Fig. 19. Comparisons of baseline period power spectra for Novel (black) and Repeat (red) items identified no differences in any ROI. No differences in power were observed in any frequency in any ROI.

L Frontal ("early" period)

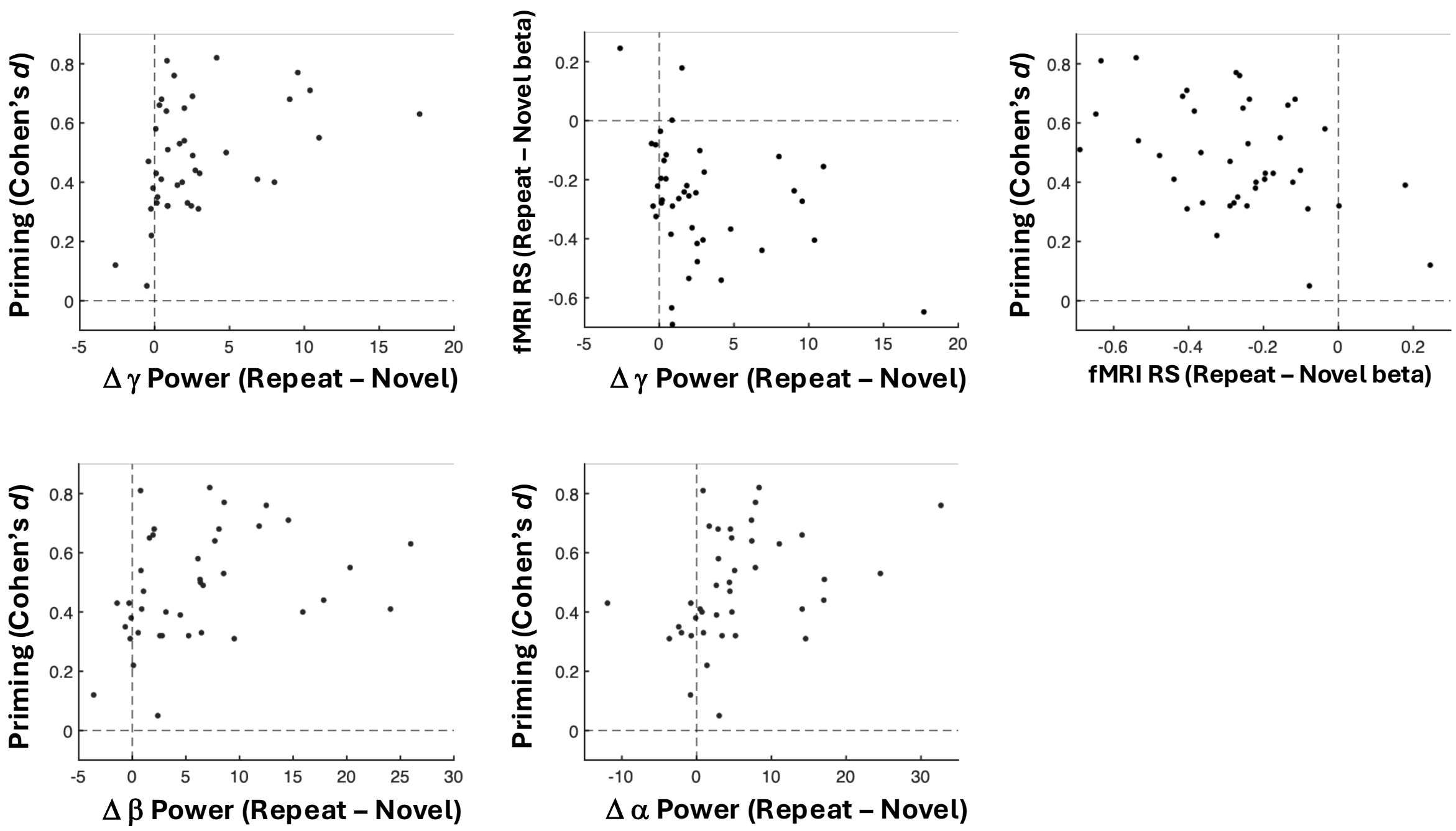

Supplementary Fig. 20. Scatterplots of raw power differences, fMRI RS differences, and priming effect sizes for the left frontal region during the early period. Results are shown for all frequency bands and variables of interest with significant correlations (Spearman) during this period.

Supplementary Table 1. Time periods of significant difference in Novel and Repeat power for each frequency and region

ROIs	Frequencies	"Early" Window (Repeat > Novel Power) Periods of FDR-corrected significance	"Late" Window (Novel > Repeat Power) Periods of FDR-corrected significance
Left Frontal	Gamma (30-80 Hz)	280 - 341 ms 427 - 854 ms	1212 - 1525 ms 1587 - 1913 ms
	Beta (13-29 Hz)	485 - 856 ms	1171 - 2060 ms
	Alpha (8-12 Hz)	613 - 888 ms	1122 - 2053 ms
	Theta (4-7 Hz)	680 - 801 ms	1276 - 1600 ms 1660 - 1888 ms
Left Fusiform	Gamma (30-80 Hz)	431 - 875 ms	1045 - 2083 ms
	Beta (13-29 Hz)	524 - 817 ms	1150 - 2073 ms 2126 - 2275 ms
	Alpha (8-12 Hz)	640 - 870 ms	1193 - 2047 ms
	Theta (4-7 Hz)	677 - 848 ms	1228 - 1642 ms 1684 - 2047 ms 2111 - 2198 ms 2318 - 2381 ms
	Delta (1-3 Hz)	698 - 844 ms	N/A
dACC/pre-SMA	Gamma (30-80 Hz)	519 - 751 ms	1096 - 1656 ms 1710 - 2192 ms
	Beta (13-29 Hz)	467 - 851 ms	1081 - 1178 ms 1203 - 2217 ms
	Alpha (8-12 Hz)	663 - 892 ms	1214 - 2201 ms
	Theta (4-7 Hz)	706 - 827 ms	1213 - 1678 ms 1771 - 1945 ms 2007 - 2138 ms
	Delta (1-3 Hz)	N/A	1438 - 1599 ms 1807 - 1894 ms
Right Fusiform	Gamma (30-80 Hz)	385 - 757 ms	1162 - 2165 ms
	Beta (13-29 Hz)	471 - 797 ms	1112 - 2209 ms
	Alpha (8-12 Hz)	607 - 917 ms	1219 - 2053 ms 2141 - 2219 ms
	Theta (4-7 Hz)	683 - 940 ms	1260 - 2403 ms 2456 - 2609 ms
	Delta (1-3 Hz)	N/A	1975 - 2114 ms

Supplementary Table 2. Correlations between EEG power differences and fMRI RS or behavioral priming by region, time period, and frequency.

			"Early" Window (Repeat > Novel Power)		"Late" Window (Novel > Repeat Power)	
			Correlation with		Correlation with	
**ROIs**	**Frequencies**		**fMRI RS**	**Priming**	**fMRI RS**	**Priming**
Left Frontal	Gamma (30-80 Hz)	Spearman *r*	-0.37	0.4236	0.2947	-0.6535
		p -value	0.0188	0.0065	0.0649	4.8362E-06
	Beta (13-29 Hz)	Spearman *r*	-0.2769	0.3992	0.2675	-0.5484
		p -value	0.0836	0.0107	0.0951	0.0002
	Alpha (8-12 Hz)	Spearman *r*	-0.2722	0.4533	0.2514	-0.5857
		p -value	0.0892	0.0033	0.1176	0.0001
	Theta (4-7 Hz)	Spearman *r*	0	0.2191	0.2263	-0.4328
		p -value	1	0.1743	0.1603	0.0053
Left Fusiform	Gamma (30-80 Hz)	Spearman *r*	0.0094	0.4106	0.349	-0.6482
		p -value	0.9542	0.0085	0.0273	6.1038E-06
	Beta (13-29 Hz)	Spearman *r*	-0.2441	0.5065	0.0233	-0.5892
		p -value	0.129	0.0009	0.8867	0.0001
	Alpha (8-12 Hz)	Spearman *r*	-0.3113	0.2388	0.1692	-0.6666
		p -value	0.0506	0.1379	0.2965	2.6488E-06
	Theta (4-7 Hz)	Spearman *r*	-0.3154	0.3031	0.2859	-0.6008
		p -value	0.0474	0.0573	0.0737	4.1567E-05
	Delta (1-3 Hz)	Spearman *r*	0.049	0.1571	N/A	N/A
		p -value	0.7641	0.333		
dACC/pre-SMA	Gamma (30-80 Hz)	Spearman *r*	-0.1096	0.4991	0.337	-0.597
		p -value	0.5009	0.001	0.0335	4.7754E-05
	Beta (13-29 Hz)	Spearman *r*	-0.0221	0.3907	0.0912	-0.6033
		p -value	0.8921	0.0127	0.5758	3.7813E-05
	Alpha (8-12 Hz)	Spearman *r*	0.0101	0.2736	0.0405	-0.4198
		p -value	0.9505	0.0876	0.8039	0.007
	Theta (4-7 Hz)	Spearman *r*	0.1814	0.0502	0.1962	-0.5622
		p -value	0.2626	0.7583	0.2249	0.0002
	Delta (1-3 Hz)	Spearman *r*	N/A	N/A	0.218	-0.391
		p -value			0.1766	0.0126
Right Fusiform	Gamma (30-80 Hz)	Spearman *r*	-0.2298	0.6191	0.1358	-0.7143
		p -value	0.1537	2.0521E-05	0.4033	2.2649E-07
	Beta (13-29 Hz)	Spearman *r*	-0.2805	0.3316	0.1484	-0.4687
		p -value	0.0796	0.0366	0.3608	0.0023
	Alpha (8-12 Hz)	Spearman *r*	-0.1895	0.2137	0.0131	-0.587
		p -value	0.2416	0.1855	0.9359	0.0001
	Theta (4-7 Hz)	Spearman *r*	-0.1133	0.2771	0.0644	-0.4934
		p -value	0.4863	0.0835	0.6932	0.0012
	Delta (1-3 Hz)	Spearman *r*	N/A	N/A	0.3304	-0.4039
		p -value			0.0373	0.0097

False Discovery Rate (*q* <.05)
p <= 0.0188

Supplementary Table 3. Differences in induced power, fMRI RS, and priming correlation values with and without ICA-based regression of the speech-associated EMG response.

			EOG + EMG Cleaning				EOG Only Cleaning			
			"Early" Window (Repeat > Novel Power)		"Late" Window (Novel > Repeat Power)		"Early" Window (Repeat > Novel Power)		"Late" Window (Novel > Repeat Power)	
			Correlation with		Correlation with		Correlation with		Correlation with	
**ROIs**	**Frequencies**		**fMRI RS**	**Priming**	**fMRI RS**	**Priming**	**fMRI RS**	**Priming**	**fMRI RS**	**Priming**
Left Frontal	Gamma (30-80 Hz)	Spearman *r*	-0.37	0.4236	0.2947	-0.6535	-0.1152	0.3606	0.2655	-0.6668
		p-value	0.0188	0.0065	0.0649	4.8362E-06	0.4791	0.0223	0.0978	2.6256E-06
	Beta (13-29 Hz)	Spearman *r*	-0.2769	0.3992	0.2675	-0.5484	-0.2677	0.3596	0.246	-0.6211
		p-value	0.0836	0.0107	0.0951	0.0002	0.0949	0.0226	0.126	0.000019
	Alpha (8-12 Hz)	Spearman *r*	-0.2722	0.4533	0.2514	-0.5857	-0.4174	0.468	0.1473	-0.5758
		p-value	0.0892	0.0033	0.1176	0.0001	0.0074	0.0023	0.3645	0.0001
	Theta (4-7 Hz)	Spearman *r*	0	0.2191	0.2263	-0.4328	-0.1957	0.2662	0.2709	-0.6695
		p-value	1	0.1743	0.1603	0.0053	0.2262	0.0969	0.0909	0.000002
Left Fusiform	Gamma (30-80 Hz)	Spearman *r*	0.0094	0.4106	0.349	-0.6482	-0.2062	0.3054	0.2914	-0.6275
		p-value	0.9542	0.0085	0.0273	6.1038E-06	0.2018	0.0553	0.0681	1.4672E-05
	Beta (13-29 Hz)	Spearman *r*	-0.2441	0.5065	0.0233	-0.5892	-0.1208	0.255	0.0171	-0.5794
		p-value	0.129	0.0009	0.8867	0.0001	0.4577	0.1123	0.9167	0.0001
	Alpha (8-12 Hz)	Spearman *r*	-0.3113	0.2388	0.1692	-0.6666	-0.3818	0.2658	0.115	-0.6152
		p-value	0.0506	0.1379	0.2965	2.6488E-06	0.0151	0.0974	0.4798	2.3961E-05
	Theta (4-7 Hz)	Spearman *r*	-0.3154	0.3031	0.2859	-0.6008	N/A	N/A	0.0165	-0.4919
		p-value	0.0474	0.0573	0.0737	4.1567E-05			0.9195	0.0013
	Delta (1-3 Hz)	Spearman *r*	0.049	0.1571	N/A	N/A	N/A	N/A	N/A	N/A
		p-value	0.7641	0.333						
dACC/pre-SMA	Gamma (30-80 Hz)	Spearman *r*	-0.1096	0.4991	0.337	-0.597	-0.2139	0.3343	0.2698	-0.6043
		p-value	0.5009	0.001	0.0335	4.7754E-05	0.1851	0.035	0.0923	3.6375E-05
	Beta (13-29 Hz)	Spearman *r*	-0.0221	0.3907	0.0912	-0.6033	-0.339	0.1908	0.0983	-0.6183
		p-value	0.8921	0.0127	0.5758	3.7813E-05	0.0324	0.2383	0.5462	2.1218E-05
	Alpha (8-12 Hz)	Spearman *r*	0.0101	0.2736	0.0405	-0.4198	0.0221	0.0576	0.0681	-0.6131
		p-value	0.9505	0.0876	0.8039	0.007	0.8921	0.7239	0.6763	2.5966E-05
	Theta (4-7 Hz)	Spearman *r*	0.1814	0.0502	0.1962	-0.5622	N/A	N/A	0.0677	-0.5957
		p-value	0.2626	0.7583	0.2249	0.0002			0.678	0.0001
	Delta (1-3 Hz)	Spearman *r*	N/A	N/A	0.218	-0.391	N/A	N/A	N/A	N/A
		p-value			0.1766	0.0126				
Right Fusiform	Gamma (30-80 Hz)	Spearman *r*	-0.2298	0.6191	0.1358	-0.7143	-0.2614	0.5648	0.0411	-0.6008
		p-value	0.1537	2.0521E-05	0.4033	2.2649E-07	0.1033	0.0001	0.8012	4.1567E-05
	Beta (13-29 Hz)	Spearman *r*	-0.2805	0.3316	0.1484	-0.4687	-0.0912	0.3206	-0.0769	-0.5006
		p-value	0.0796	0.0366	0.3608	0.0023	0.5758	0.0437	0.6371	0.001
	Alpha (8-12 Hz)	Spearman *r*	-0.1895	0.2137	0.0131	-0.587	-0.0794	0.349	-0.0951	-0.4544
		p-value	0.2416	0.1855	0.9359	0.0001	0.6264	0.0273	0.5593	0.0032
	Theta (4-7 Hz)	Spearman *r*	-0.1133	0.2771	0.0644	-0.4934	-0.0996	0.2586	-0.0191	-0.3383
		p-value	0.4863	0.0835	0.6932	0.0012	0.5408	0.1072	0.9067	0.0327
	Delta (1-3 Hz)	Spearman *r*	N/A	N/A	0.3304	-0.4039	N/A	N/A	0.0203	-0.1161
		p-value			0.0373	0.0097			0.9012	0.4756

False Discovery Rate (*q* <.05)
p <= 0.0188

False Discovery Rate (*q* <.05)
p <= 0.0151

Supplementary Table 4. Effect of partialling mean RT on correlations between power, fMRI RS, and priming.

ROIs	Frequencies		"Early" Window (Repeat > Novel Power) Correlation with fMRI RS	Priming	"Late" Window (Novel > Repeat Power) Correlation with fMRI RS	Priming
Left Frontal	Gamma (30-80 Hz)	Spearman *r*	-0.3833	0.4239	0.3003	-0.6535
		p -value	0.016	0.0072	0.0633	6.47E-06
	Beta (13-29 Hz)	Spearman *r*	-0.3143	0.405	0.2659	-0.5488
		p -value	0.0514	0.0105	0.1018	0.0003
	Alpha (8-12 Hz)	Spearman *r*	-0.3666	0.4899	0.2567	-0.5857
		p -value	0.0217	0.0015	0.1146	0.0001
	Theta (4-7 Hz)	Spearman *r*	-0.123	0.2725	0.239	-0.4334
		p -value	0.4556	0.0933	0.1428	0.0058
Left Fusiform	Gamma (30-80 Hz)	Spearman *r*	0.0077	0.4106	0.3922	-0.6778
		p -value	0.9627	0.0094	0.0135	2.14E-06
	Beta (13-29 Hz)	Spearman *r*	-0.2754	0.5234	0.0298	-0.5907
		p -value	0.0898	0.0006	0.8571	0.0001
	Alpha (8-12 Hz)	Spearman *r*	-0.383	0.262	0.1871	-0.6766
		p -value	0.0161	0.1071	0.254	2.26E-06
	Theta (4-7 Hz)	Spearman *r*	-0.3987	0.3395	0.3081	-0.6113
		p -value	0.0119	0.0345	0.0563	3.57E-05
	Delta (1-3 Hz)	Spearman *r*	0.0249	0.1642	N/A	N/A
		p -value	0.8802	0.3179		
dACC/pre-SMA	Gamma (30-80 Hz)	Spearman *r*	-0.1202	0.5033	0.3318	-0.6019
		p -value	0.466	0.0011	0.0391	0.0001
	Beta (13-29 Hz)	Spearman *r*	-0.0478	0.413	0.0931	-0.6034
		p -value	0.7724	0.009	0.573	4.78E-05
	Alpha (8-12 Hz)	Spearman *r*	-0.0106	0.2852	0.047	-0.4212
		p -value	0.949	0.0785	0.7762	0.0076
	Theta (4-7 Hz)	Spearman *r*	0.1689	0.0548	0.2053	-0.5651
		p -value	0.3041	0.7404	0.2098	0.0002
	Delta (1-3 Hz)	Spearman *r*	N/A	N/A	0.2173	-0.3911
		p -value			0.1838	0.0138
Right Fusiform	Gamma (30-80 Hz)	Spearman *r*	-0.2244	0.6263	0.1293	-0.7218
		p -value	0.1697	2.00E-05	0.4326	2.15E-07
	Beta (13-29 Hz)	Spearman *r*	-0.2773	0.3469	0.1475	-0.4687
		p -value	0.0874	0.0305	0.3702	0.0026
	Alpha (8-12 Hz)	Spearman *r*	-0.1817	0.2236	0.0227	-0.5959
		p -value	0.2682	0.1712	0.891	0.0001
	Theta (4-7 Hz)	Spearman *r*	-0.105	0.2815	0.073	-0.4989
		p -value	0.5245	0.0825	0.6587	0.0012
	Delta (1-3 Hz)	Spearman *r*	N/A	N/A	0.3272	-0.4149
		p -value			0.042	0.0086

False Discovery Rate (*q* <.05)
p <= 0.0217
