# Supplementary material for: Repetition-related reductions in neural activity support improved behavior through increases in oscillatory power
Source: Commun Biol. 2026 Jul 21;9:1321. doi: 10.1038/s42003-026-10668-0 (PMC13652815; doi:10.1038/s42003-026-10668-0)
Supplement: Supplementary file 3 [file 42003_2026_10668_MOESM3_ESM.pdf]

Description of Additional Supplementary File

Supplementary Movie 1. A lateral view of the left hemisphere showing induced power differences between Repeat and Novel conditions in the gamma band over time in the overt naming data. Warm colors reflect greater power for the Repeat condition and cool colors reflect greater power for the Novel condition.

Supplementary Movie 2. A ventral view of the brain showing induced power differences between Repeat and Novel conditions in the gamma band over time in the overt naming data. Warm colors reflect greater power for the Repeat condition and cool colors reflect greater power for the Novel condition.

Supplementary Movie 3. A lateral view of the left hemisphere showing induced power differences between Repeat and Novel conditions in the gamma band over time in the covert naming data. Warm colors reflect greater power for the Repeat condition and cool colors reflect greater power for the Novel condition.

Supplementary Movie 4. A ventral view of the brain showing induced power differences between Repeat and Novel conditions in the gamma band over time in the covert naming data. Warm colors reflect greater power for the Repeat condition and cool colors reflect greater power for the Novel condition.

Supplementary Data 1. Numerical source data for bar graphs and scatterplots in main manuscript figures.
