# Supplementary material for: Repetition-related reductions in neural activity support improved behavior through increases in oscillatory power
Source: Commun Biol. 2026 Jul 21;9:1321. doi: 10.1038/s42003-026-10668-0 (PMC13652815; doi:10.1038/s42003-026-10668-0)
Supplement: Supplementary file 4 [file 42003_2026_10668_MOESM9_ESM.pdf]

nature portfolio

Corresponding author(s): Adrian W. Gilmore; Stephen J. Gotts

Last updated by author(s): 6/25/2026

Reporting Summary

Nature Portfolio wishes to improve the reproducibility of the work that we publish. This form provides structure for consistency and transparency in reporting. For further information on Nature Portfolio policies, see our Editorial Policies and the Editorial Policy Checklist.

Statistics

For all statistical analyses, confirm that the following items are present in the figure legend, table legend, main text, or Methods section.

n/a	Confirmed	
☐	☒	The exact sample size (*n*) for each experimental group/condition, given as a discrete number and unit of measurement
☐	☒	A statement on whether measurements were taken from distinct samples or whether the same sample was measured repeatedly
☐	☒	The statistical test(s) used AND whether they are one- or two-sided<br>*Only common tests should be described solely by name; describe more complex techniques in the Methods section.*
☐	☒	A description of all covariates tested
☐	☒	A description of any assumptions or corrections, such as tests of normality and adjustment for multiple comparisons
☐	☒	A full description of the statistical parameters including central tendency (e.g. means) or other basic estimates (e.g. regression coefficient) AND variation (e.g. standard deviation) or associated estimates of uncertainty (e.g. confidence intervals)
☐	☒	For null hypothesis testing, the test statistic (e.g. F, t, r) with confidence intervals, effect sizes, degrees of freedom and P value noted<br>*Give P values as exact values whenever suitable.*
☒	☐	For Bayesian analysis, information on the choice of priors and Markov chain Monte Carlo settings
☒	☐	For hierarchical and complex designs, identification of the appropriate level for tests and full reporting of outcomes
☐	☒	Estimates of effect sizes (e.g. Cohen's *d*, Pearson's *r*), indicating how they were calculated

Our web collection on statistics for biologists contains articles on many of the points above.

Software and code

Policy information about availability of computer code

Data collection	PsychoPy2 for stimulus presentation and button press recording<br>Adobe Audition for voice recording<br>Commercial code bundled with the GE Discovery 750 scanner for fMRI data collection<br>Commercial code bundled with EGI NetStation for EEG recording
Data analysis	SPSS (29.0.2) and Matlab (2021b) for behavioral data analysis<br>AFNI (23.3.09) for fMRI data analysis<br>EGI Netstation (5.4.2) and MNE-Python (1.8) for EEG analysis<br>Matlab (2021) for all correlation analyses

For manuscripts utilizing custom algorithms or software that are central to the research but not yet described in published literature, software must be made available to editors and reviewers. We strongly encourage code deposition in a community repository (e.g. GitHub). See the Nature Portfolio guidelines for submitting code & software for further information.

Data

Policy information about availability of data

All manuscripts must include a data availability statement. This statement should provide the following information, where applicable:

- Accession codes, unique identifiers, or web links for publicly available datasets
- A description of any restrictions on data availability
- For clinical datasets or third party data, please ensure that the statement adheres to our policy

Data and code associated with this research are available through the Harvard Dataverse, https://dataverse.harvard.edu/dataverse/NamingEEGfMRI.

Research involving human participants, their data, or biological material

Policy information about studies with human participants or human data. See also policy information about sex, gender (identity/presentation), and sexual orientation and race, ethnicity and racism.

Reporting on sex and gender	Sexes were determined based on self report and responses are summarized in the Methods of the manuscript. In the overt naming sample, 18/40 participants self-reported as male and 22/40 self-reported as female. In the covert naming sample, 10/25 participants reported as male and 15/25 reported as female. Sex differences were not analyzed as prior literature has not suggested that any differences should emerge between males and females.
Reporting on race, ethnicity, or other socially relevant groupings	Participants were not categorized into distinct racial, ethnic, or other social groups.
Population characteristics	All participants were between 18-35 years old, were right-handed, had normal or corrected-to-normal vision, reported no history of psychiatric or neurologic illness, and were native speakers of English. These were not used as covariates in any analyses.
Recruitment	Participants were recruited from the National Institutes of Health and surrounding area.
Ethics oversight	Informed consent was obtained from all participants and the experiment was approved by the NIH Institutional Review Board (Protocol 93-M-0170; clinical trials number NCT00001360).

Note that full information on the approval of the study protocol must also be provided in the manuscript.

Field-specific reporting

Please select the one below that is the best fit for your research. If you are not sure, read the appropriate sections before making your selection.

☐ Life sciences ☒ Behavioural & social sciences ☐ Ecological, evolutionary & environmental sciences

For a reference copy of the document with all sections, see nature.com/documents/nr-reporting-summary-flat.pdf

Behavioural & social sciences study design

All studies must disclose on these points even when the disclosure is negative.

Study description	Quantitative experimental study
Research sample	Participants included 18-35 year old males and females from the National Institutes of Health and surrounding area.
Sampling strategy	The sampling procedure used was convenience sampling, as eligible participants elected to participate specifically in this study. Sample sizes were based on prior fMRI experiments that used the same stimuli and same basic paradigm as that employed here (Gotts et al., 2021; Gilmore et al., 2019).
Data collection	A macbook pro running PsychoPy2 was used to present stimuli and record responses during the initial (pre-scan) naming. In-scanner verbal responses were spoken into a noise-canceling, MR-compatible Optoacoustics FOMRI-III NC microphone, connected to an M-Audio FastTrack Ultra 8-R USB audio/MIDI interface. A Dell Precision M4400 laptop recorded responses using Adobe Audition CS 6. FMRI data were collected on a 3T GE Discovery MR750 scanner using a 32-channel head coil. EEG data were collected using a 256-electrode (Ag/AgCl) HydroCel GSN 220MR Geodesic Sensor Net that was connected to a Net Amps 400 amplifier and were recorded onto a Macbook Pro laptop.
Timing	The overt naming experiment was conducted between Sept, 2021 and March, 2023. The covert naming experiment was conducted between April, 2023 and May, 2024.
Data exclusions	Five overt naming participants were excluded (1 after detecting an anatomical abnormality, 1 for excessive in-scanner motion, two for equipment malfunctions, and 1 due to vision that could not be corrected using MR-compatible glasses.)

Non-participation	No participants dropped out of this study.
Randomization	Participants were randomly assigned to different counterbalances.

Reporting for specific materials, systems and methods

We require information from authors about some types of materials, experimental systems and methods used in many studies. Here, indicate whether each material, system or method listed is relevant to your study. If you are not sure if a list item applies to your research, read the appropriate section before selecting a response.

Materials & experimental systems

n/a	Involved in the study
☒	☐ Antibodies
☒	☐ Eukaryotic cell lines
☒	☐ Palaeontology and archaeology
☒	☐ Animals and other organisms
☒	☐ Clinical data
☒	☐ Dual use research of concern
☒	☐ Plants

Methods

n/a	Involved in the study
☒	☐ ChIP-seq
☒	☐ Flow cytometry
☐	☒ MRI-based neuroimaging

Plants

Seed stocks	n/a
Novel plant genotypes	n/a
Authentication	n/a

Magnetic resonance imaging

Experimental design

Design type	Event-related
Design specifications	Participants identified 100 novel and 100 repeat (previously encountered) stimuli across 4 task scans. Each scan consisted of 25 novel and 25 repeat stimuli.
Behavioral performance measures	Verbal responses were scored as accurate or inaccurate. Onset time differences between a square pulse that marked each trial's onset in the audio recording, and onset and the voice response in each trial, were used to calculate voice RTs

Acquisition

Imaging type(s)	Functional and structural scans were collected
Field strength	3T
Sequence & imaging parameters	Functional images were acquired using a BOLD-contrast sensitive multi-echo echo-planar sequence (Array Spatial Sensitivity Encoding Technique or ASSET acceleration factor = 2; TEs = 13.5, 34.2, 54.8, and 75.5 ms; TR = 2150 ms, flip angle = 75°; 72 × 72 matrix, in-plane resolution = 3.0 mm). Whole brain EPI volumes (MR frames) consisted of 42 interleaved, 3.0 mm-thick oblique axial slices, aligned manually to the AC-PC axis.<br><br>Structural MPRAGE images were also collected for each participant (TE = 3.47 ms, TR = 2.53s, TI = 900 ms, flip angle = 7°, 172 slices with 1×1×1mm voxels).
Area of acquisition	Whole-brain volumes were collected.

Diffusion MRI ☐ Used ☒ Not used

Preprocessing

Preprocessing software	Freesurfer (7.2) was used to initially process anatomical data, which were then processed in AFNI. Specific steps included skull-stripped using @SSwarper, and standard SUMA std.141 surface meshes for each participant were generated using @SUMA_Make_Spec_FS,<br><br>The first 4 MR frames of each EPI scan run were excluded to allow for T1 equilibration. Blip-up/blip-down distortion correction was carried out using AFNI's unWarpEPI.py script for each run using the scan run with an inverted phase encoding direction. EPI data were realigned to the first volume of each scan run via rigid body transformation and subsequently registered to the anatomical image. Motion was calculated using the second echo of each run and motion estimates were retained for use as nuisance regressors during subsequent general linear model (GLM) analysis. Functional data then underwent multi-echo ICA analysis using tedana.py software. Denoised timeseries data were mapped to each participant's std.141 surface mesh and a geodesic blur along the unfolded cortical surface was applied to achieve a FWHM of 8 mm. Data were then normalized and rescaled to percent signal change.
Normalization	std.141 surface mesh and a geodesic blur along the unfolded cortical surface was applied to achieve a FWHM of 8 mm. Data were then normalized and rescaled to percent signal change.
Normalization template	All data were sampled to the std.141 surface mesh.
Noise and artifact removal	Six motion parameters were included as regressors during GLM creation. In addition, prior to GLM creation, data were denoised using multi-echo ICA via tedana.
Volume censoring	Single TRs were censored if frame-to-frame motion exceeded a Euclidian Norm of 0.3 mm or if ≥ 10% of voxels in that TR were flagged as outliers by AFNI's 3dToutcount. This resulted in an average of 6±2% of TRs being censored for Overt Naming participants and 5±1% for the Covert Naming participants.

Statistical modeling & inference

Model type and settings	Mass univariate analysis was conducted. Novel and Repeat items responses were estimated using TENT functions (meaning no specific response shape was selected a priori).
Effect(s) tested	For the purposes of statistical testing, response magnitudes were estimated for Novel and Repeat conditions by averaging the 3rd and 4th time points of the TENT function (reflecting the expected peak of the BOLD response, 4.3 to 8.6 s following stimulus onset). These were then contrasted against implicit baseline and against each other at the group level across the entire surface mesh.

Specify type of analysis: ☒ Whole brain ☐ ROI-based ☐ Both

Statistic type for inference<br>(See Eklund et al. 2016)	Vertex-wise correction for multiple comparisons was accomplished using the SurfClust function in AFNI, which uses the smoothness of the data to generate null surface maps and empirically defines a minimum cluster size to achieve a given whole-brain correction at a given significance level. A vertex-wise threshold of $p < .001$ and a minimum cluster extent of 195 vertices achieved a corrected $p < .05$.
Correction	Cluster-based and FDR corrections were applied in different analyses.

Models & analysis

n/a	Involved in the study
☒	☐ Functional and/or effective connectivity
☒	☐ Graph analysis
☒	☐ Multivariate modeling or predictive analysis
